# Quantitative flow cytometry enables end-to-end optimization of cross-platform extracellular vesicle studies

**DOI:** 10.1016/j.crmeth.2023.100664

**Published:** 2023-12-18

**Authors:** Sean Cook, Vera A. Tang, Joanne Lannigan, Jennifer C. Jones, Joshua A. Welsh

**Affiliations:** 1 Laboratory of Pathology, Translational Nanobiology Section, Centre for Cancer Research, National Institute of Health, National Institutes of Health, Bethesda, MD, USA; 2 Faculty of Medicine, Department of Biochemistry, Microbiology, and Immunology, University of Ottawa, Flow Cytometry and Virometry Core Facility, Ottawa, ON K1H 8M5, Canada; 3 Flow Cytometry Support Services, Alexandria, VA, USA

**Keywords:** calibration, extracellular particles, extracellular vesicles, exosomes, FCM_PASS_ software, quantitative flow cytometry, optimization pipeline, viruses

## Abstract

Flow cytometry (FCM) is a common method for characterizing extracellular particles (EPs), including viruses and extracellular vesicles (EVs). Frameworks such as MIFlowCyt-EV exist to provide reporting guidelines for metadata, controls, and data reporting. However, tools to optimize FCM for EP analysis in a systematic and quantitative way are lacking. Here, we demonstrate a cohesive set of methods and software tools that optimize FCM settings and facilitate cross-platform comparisons for EP studies. We introduce an automated small-particle optimization (SPOT) pipeline to optimize FCM fluorescence and light scatter detector settings for EP analysis and leverage quantitative FCM (qFCM) as a tool to further enable FCM optimization of fluorophore panel selection, laser power, pulse statistics, and window extensions. Finally, we demonstrate the value of qFCM to facilitate standardized cross-platform comparisons, irrespective of instrument configuration, settings, and sensitivity, in a cross-platform standardization study utilizing a commercially available EV reference material.

## Highlights

• The SPOT pipeline optimizes quantitative flow cytometry of extracellular particles
• SPOT facilitates standardized cross-platform comparisons for qFCM
• SPOT enables optimization of fluorophore selection and laser and detector settings

## Motivation

Small-particle flow cytometry is increasingly being utilized on instruments primarily developed for cellular analysis. To date, there has been a lack of instrument agnostic methods to characterize and optimize flow cytometer performance for small-particle applications.

## Graphical abstract

**Figure:**
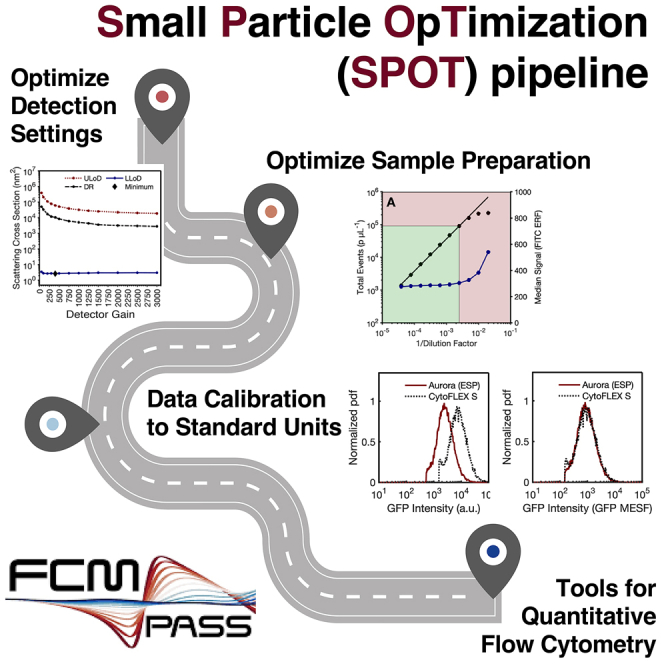

## Teaser

Cook et al. demonstrate the utility of flow cytometer detector setting optimization and calibration for small-particle analysis and cross-platform comparisons.

## Introduction

Flow cytometers were first developed in the 1960s for fluorescence-based detection of cells.^1^ Today, their design is still primarily focused on cellular phenotyping but with increased throughput and multi-dimensionality.^2^^,^^3^ As the use of flow cytometry (FCM) as a technique has become common place in research institutions, alternative applications have been explored. Today, FCM is increasingly being utilized to characterize sub-micron particles in the form of extracellular particles (EPs), which include extracellular vesicles (EVs) and viruses.^4^^,^^5^^,^^6^^,^^7^^,^^8^ These particles are orders of magnitude smaller and dimmer than most FCM equipment was originally intended to characterize.

As utilization of FCM for EP analysis has increased so too has the awareness of its limitations and the lack of reproducibility and validity of published data.^9^^,^^10^^,^^11^ In 2020, the MIFlowCyt-EV framework was published as a position paper delineating minimal reporting standards for EV FCM.^12^ This work was a product of a 5-year collaboration of international researchers from the International Society of Extracellular Vesicles (ISEV), the International Society for Advancement of Cytometry (ISAC), and the International Society for Thrombosis and Haemostasis (ISTH), which formed an intersocietal EV FCM working group in 2015. This reporting framework outlines key metadata, controls, calibration, and data reporting fields that should be completed when undertaking small-particle measurements using FCM. While reporting criteria have been established, there is an unmet need for FCM tools to facilitate increased reproducibility and validity of EPs that will in turn aid in the reliability and correct interpretation of published data.

**Figure 1:**
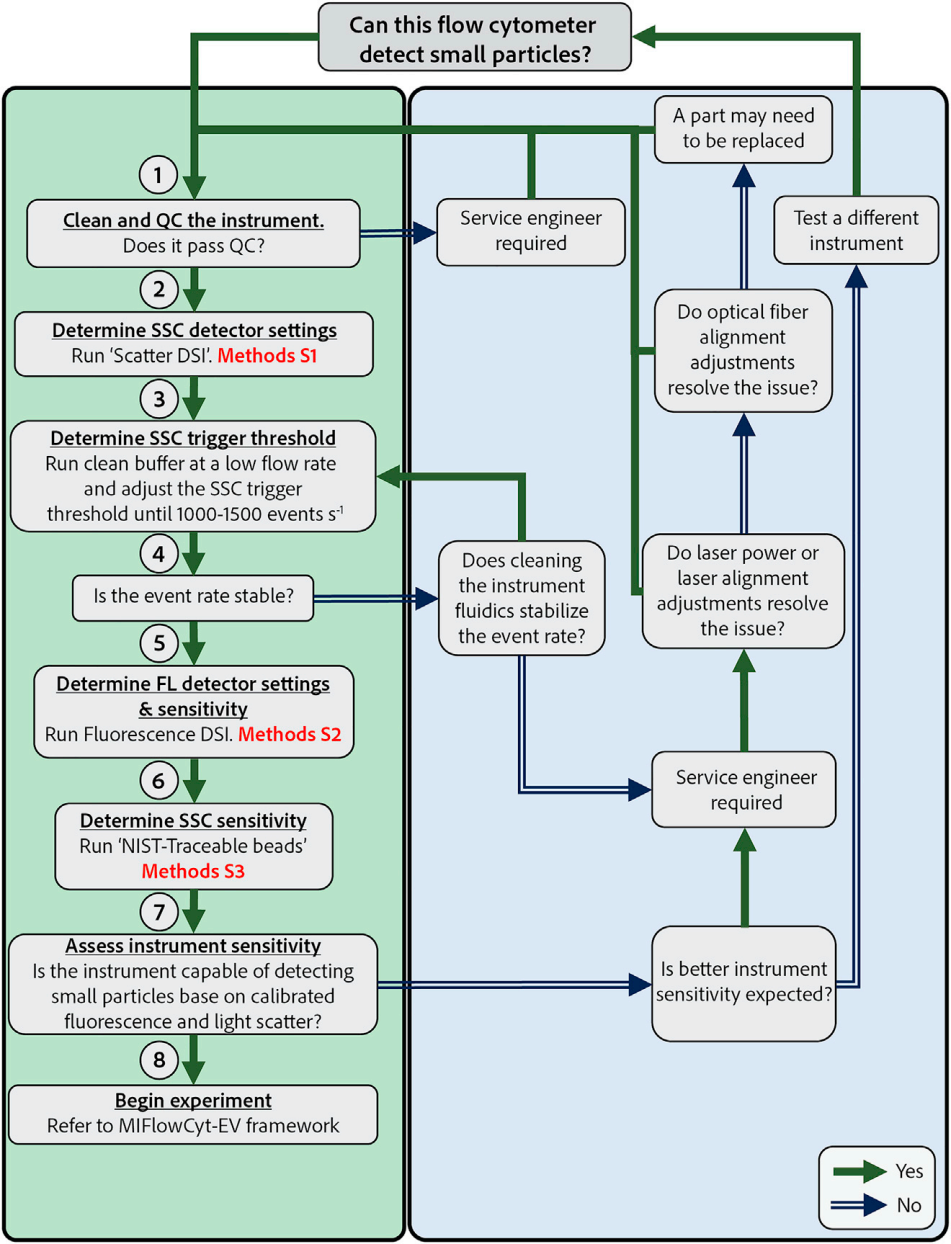
Decision tree for assessing the suitability of a flow cytometer for small-particle analysis

Robust detection of EPs using FCM requires systematic and rigorous optimization of flow cytometer configuration and settings. Furthermore, the characterization of EPs themselves requires quantitative metrics to enable longitudinal utility and intrainstrument comparisons. In order to address these outstanding requirements in the field, we developed a small-particle optimization (SPOT) pipeline utilizing quantitative FCM (qFCM; Figure 1).

In the small-particle FCM field, the term “calibration” has been interpreted in multiple ways. Commonly, simply analyzing beads and drawing gates between the populations has been marketed and published in the small-particle field as "calibration" and has been the product of early standardization initiatives.^13^^,^^14^ Here, we introduce qFCM as a term for the conversion of arbitrary units (a.u.) to standard units in order to avoid confusion with existing literature and reagent implementations. qFCM is essential for EP FCM, owing to commercially available equipment being unable to detect the full distribution of EVs from complex biofluids such as plasma and cerebrospinal fluid. This limitation results in different equipment detecting different portions of the same population, and without quantitating the data in standard units, comparison between platforms become infeasible, limiting the utility of data. Here, we not only demonstrate the ability of qFCM to characterize instrument performance and enable optimization of the instrument, but we also demonstrate that qFCM facilitates cross-platform comparisons irrespective of instrument platform, configuration, and settings.

All flow cytometers have daily quality control measures that track longitudinal performance. However, these measures are sub-optimal for small-particle analysis, as they have been developed for cellular analysis, which has different optimization considerations and requirements. Protocols currently developed for optimization of the cellular detector settings commonly derive the “minimum detector settings.”^15^ These settings are derived to minimize electronic noise while maximizing dynamic range. Furthermore, these settings are based on the acquisition of large, bright beads that have hundreds of thousands of copies of a given fluorophore on their surface. Utilizing qFCM, we developed the SPOT pipeline to automate the derivation of optimal small-particle fluorescence and light scatter detection settings in order to maximize the sensitivity of the FCM platform and derive instrument sensitivity in quantitative units. We go on to validate the performance of these derived settings using beads with commercially available EV reference materials.

## Results

### qFCM enables cross-platform comparisons

To quantitatively characterize the performance of an instrument, a standard metric for the parameters measured must be identified. The light detected in FCM in the form of scatter (SSC) and fluorescence (FL) is reported in a.u. and does not allow for direct comparisons between instruments. Due to the broad range in cellular epitope abundance, detector optimization methods to date have focused on the minimal setting to reduce the effects of electronic noise on the detection of cells.^15^ These methods use a bright bead population(s) and identify the detector setting by using the inflection point of the detector setting versus the coefficient of variation.

**Figure 2:**
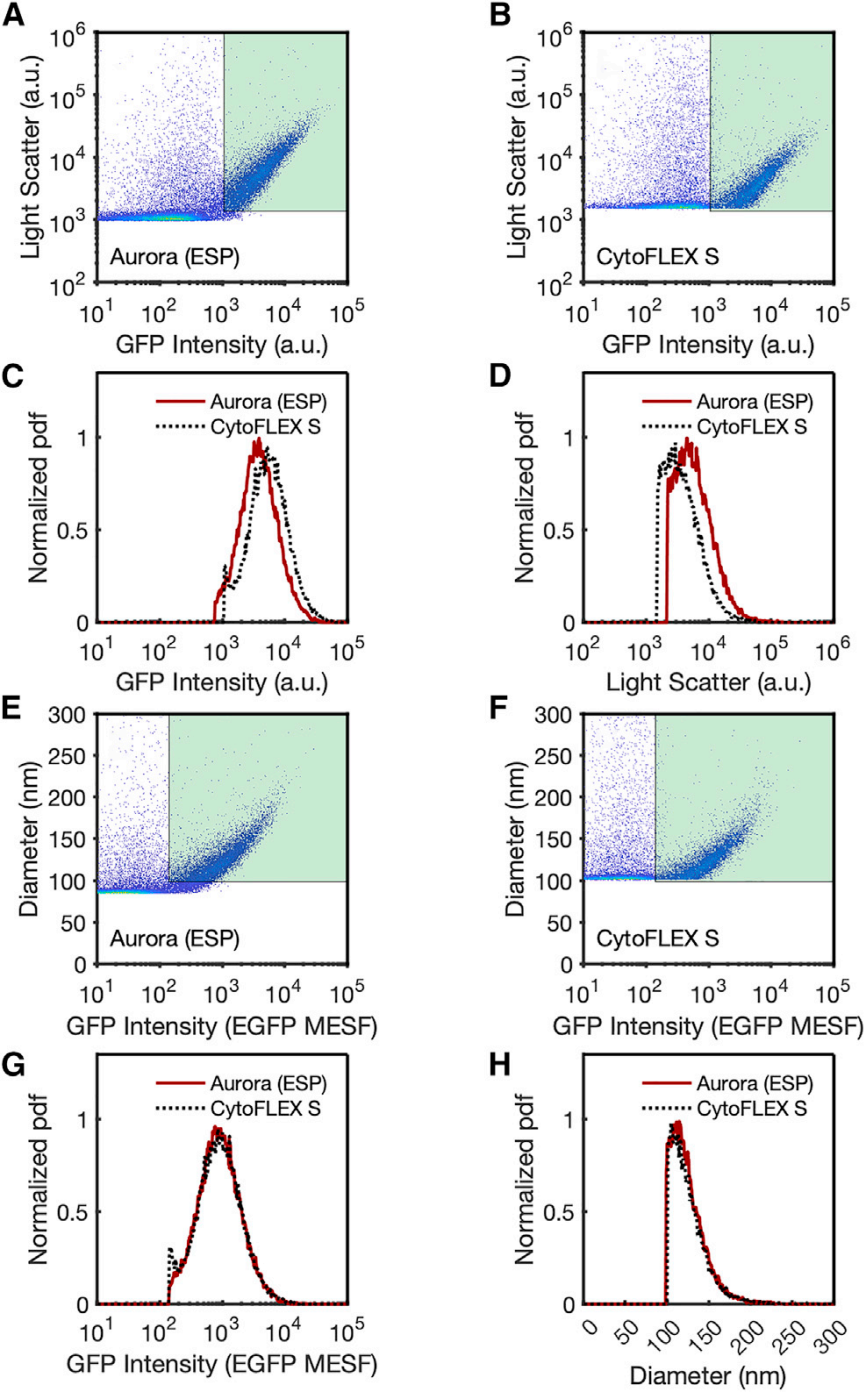
Fluorescence and light scatter calibration allow EV intrainstrument comparisons (A) rEVs acquired on the Aurora platform plotted in arbitrary units. (B) rEVs acquired on the CytoFLEX platform plotted in arbitrary units. The green region denotes the gated area of the CytoFLEX platform in arbitrary units. (C) Comparison of GFP intensity in arbitrary units from gated (green region in plot A-B) Aurora (solid red line) and CytoFLEX S (dotted black line) data. (D) Comparison of SSC intensity in arbitrary units from gated (green region in plot A-B) Aurora (solid red line) and CytoFLEX S (dotted black line) data. (E) rEVs acquired from the Aurora platform plotted in calibrated units. (F) rEVs acquired from the CytoFLEX platform plotted in calibrated units. The green region denotes the gated area of the CytoFLEX platform in calibrated units. (G) Comparison of GFP intensity in calibrated units from gated (green region in plot E-F) Aurora (solid red line) and CytoFLEX S (dotted black line) data as normalized probability distribution functions (pdfs). (H) Comparison of SSC intensity in calibrated units from gated (green region in plot A-B) Aurora (solid red line) and CytoFLEX S (dotted black line) data.

Since the signal intensities associated with cells are well within the range of detection for commercial flow cytometers, the quantitative characterization of the limits of detection for these instruments is not prioritized for the majority of FCM users. Using qFCM to derive limits of detection has been necessitated by the fact that current commercially available flow cytometers lack the sensitivity to detect the full distribution of EVs from complex biofluids such as plasma and cerebrospinal fluid. In this application, it becomes important for the limit of detection to be quantitatively defined and optimized for each instrument to allow for the greatest detection of the EV population being characterized. Therefore, utilizing qFCM, rather than a.u. FCM, is necessary for comparisons to be made between platforms. To illustrate this, the analysis of recombinant EVs (rEVs) expressing EGFP, a commercially available reference material, is shown on two flow cytometers as reported in a.u. (Figures 2A and 2B). A gate using the limits of detection from the CytoFLEX cytometer in a.u. was applied to the data of the same sample collected on the Aurora platform. When comparing the gated data, shown in Figures 2C and 2D, there is poor concordance between the populations from the same rEV sample analyzed by the CytoFLEX and Aurora on both fluorescence and light scatter. The uncalibrated fluorescence intensities on the CytoFLEX and Aurora were 5.4 × 10^3^ and 3.8 × 10^3^ a.u., and the uncalibrated light scatter intensities were 3.5 × 10^3^ and 5.4 × 10^3^ a.u., respectively. Upon calibrating the EGFP intensity from a.u. to molecules of equivalent soluble fluorophore (MESF) and the light scatter intensity to units of diameter in nanometers and then gating the populations (Figures 2E and 2F), concordance is greatly improved between platforms. Calibrated data between the CytoFLEX and Aurora platforms had median fluorescence intensities of 882 and 875 EGFP MESF (Figure 2G), and diameters of 120.8 and 120.3 nm (Figure 2H), respectively. These data demonstrate that irrespective of instrument platform, sensitivity, and settings, data can be compared when qFCM is utilized, whereas direct comparisons between a.u. FCM data cannot be made.

### Identifying flow cytometer limits of detection and optimizing detector settings

**Figure 3:**
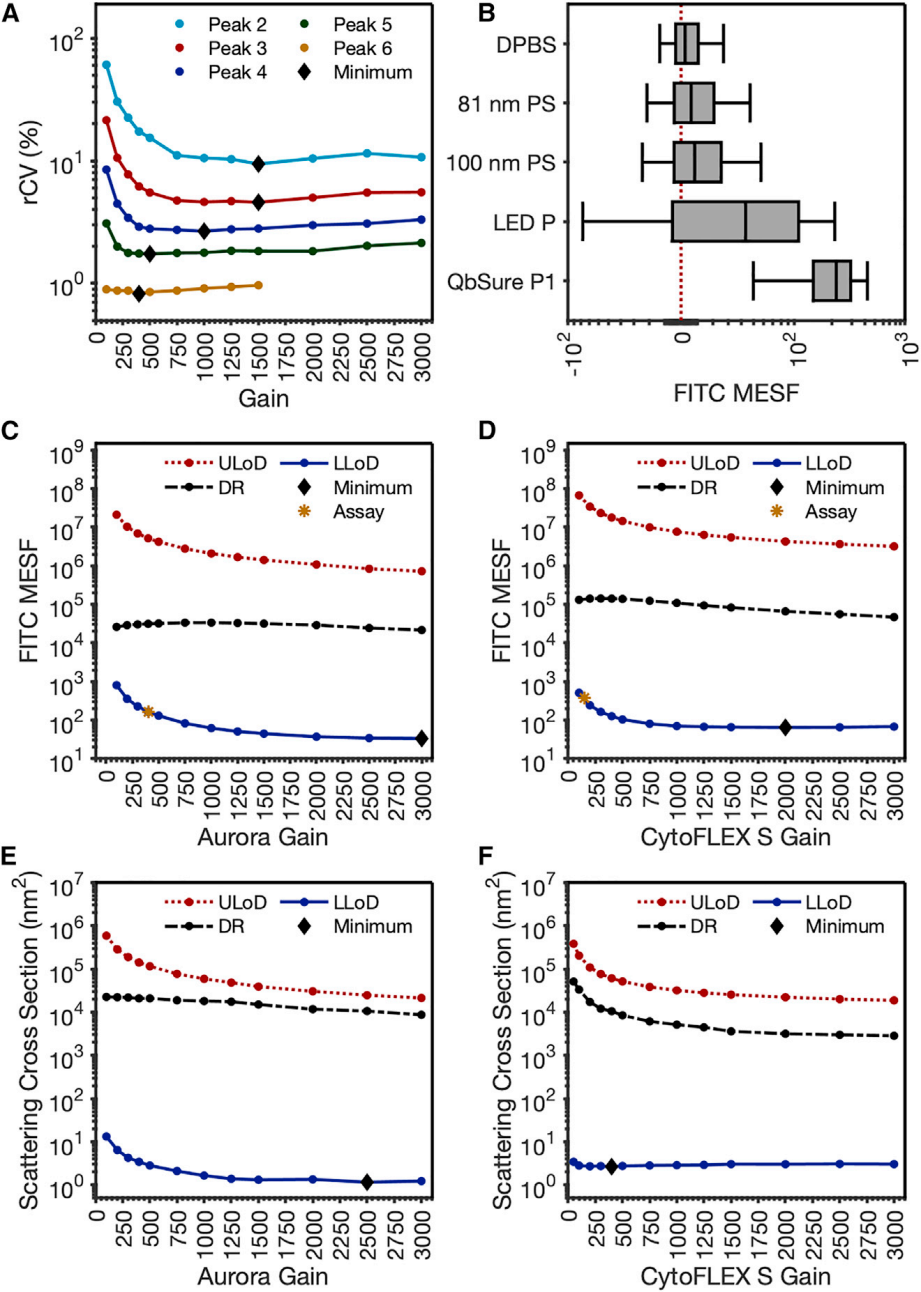
Development of a small-particle detector setting optimization methodology (A) Percentage of CV of QbSure rainbow multi-peak bead populations across a range of detector settings on the Aurora. The minimum percentage of CV per population is denoted by a black diamond. (B) Comparison of different methods for quantification of the lower limit of detection (LLoD) on the Aurora platform. Red dotted line indicates 0. (C and D) Aurora (C) and CytoFLEX (D) LLoD, upper LoD (ULoD), and dynamic range (DR) default instrument settings (Assay) of their FITC channel as a function of gain setting using FCM_PASS_ fluorescent detector optimization protocol. (E and F) Aurora (E) and CytoFLEX (F) scatter LLoD, ULoD, and DR as a function of gain setting using FCM_PASS_ light scatter detector optimization protocol.

When data are acquired on a flow cytometer, they are scaled on a.u. axes. While the number of photons reaching the detector can be constant, altering the detector settings amplifies the photons that have reached the detector and transposes the data up and down this arbitrary axis. To date, optimizing and maintaining detector settings for cellular analysis has typically been achieved by assessing the coefficient of variation (CV) in a reference bead population and identifying the detector setting at which the bead fluorescence variation plateaus (Figure 3A).^15^ Due to cells having orders of magnitude more epitopes for labeling than EPs, dynamic range is a concern that must be balanced. Full separation of positive versus negatively stained cells can therefore be achieved using detector settings with reduced sensitivity or by titrating antibodies, resulting in dimmer stained populations. The requirements for small, dim particles are not the same for those of cells. Due to many EP derivations having a log-normal distribution with a modal point ≲100 nm and having <50 copies of any given epitope, detector sensitivity must be maximized due to the majority of EPs being undetectable. The use of minimum CV for a bright bead population is not sufficient for identifying optimal settings for dim signals, as the detector settings at which the minimum CV is reached are dependent on how bright the signal is (Figure 3A). This is demonstrated with a set of 6-peak rainbow beads, where the minimum CV for the brightest population is reached at a gain of 400, while the second dimmest population reaches a minimum CV at a gain of 1,500.

The criteria for optimizing detector settings for EPs are different than those for cells. With EPs, full separation of positively and negatively stained populations is often unachievable due to sensitivity limitations. With limitations in sensitivity and the lower abundance of epitopes, dynamic range is less often a concern when optimizing detector settings for relatively small signals. Therefore, the most sensitive detector settings on photon multiplier tubes (PMTs) and avalanche photodiode (APD)-based flow cytometers tend to have sufficient sensitivity to detect a portion of EPs. Most critically, the derivation of optimal detector settings for small particles relies on having the ability to determine an instruments lower limit of detection (LoD). Commonly in spectrometric assays, this is derived by having a “blank” control. Typically, FCM reference materials are in the form of beads due to low cost, stability, and ease of use, and have tunable properties such as size, fluorescence, and refractive index (RI).

Multi-peak rainbow beads (QbSure, ∼3 μm), polystyrene nanoparticles (81, 100 nm), an LED pulser, and instrument opto-electronic noise were compared to understand their fluorescence intensity and distribution (Figure 3B). When compared, instrument opto-electronic noise had the lowest 95^th^ percentile intensity of 27 fluorescein isothiocyanate (FITC) MESF, with the dimmest rainbow peak bead having the highest 95^th^ percentile intensity at 421 MESF (statistics summarized in Table S1). All distributions when tested for normality had significantly nonnormal distribution with various levels of skewness (Table S1). From this, we have demonstrated that triggering on opto-electronic noise using Dulbecco's phosphate buffered saline (DPBS) as a sample is the most reliable method of determining the instrument’s lower LoD.

By normalizing the opto-electronic noise as a reference population across detector setting incrementation (DSI) either using qFCM or normalizing to a bright fluorescent particle that is detectable at all gains, it is possible to identify the settings at which the instrument has the greatest sensitivity for fluorescence (Figures 3C and 3D**)** and light scatter (Figures 3E and 3F). It is notable that maximal sensitivity on the Aurora and CytoFLEX platforms for fluorescence and light scattering was not always achieved simply by increasing the detectors to their maximum settings. This analysis procedure, along with automated bead cross-calibration, was built into an automated peak gating and analysis function within FCM_PASS_ software to allow ergonomic assessment of optimal instrument detector settings and sensitivities in calibrated units. Currently, commercial flow cytometers only optimize fluorescent detector settings for fluorescence. Here, we show that compared to default cellular settings, we achieved a 4.91-fold increase in sensitivity on the Aurora platform and a 5.80-fold increase on the CytoFLEX platform using the SPOT pipeline.

### Validating optimized detector settings on EV detection

**Figure 4:**
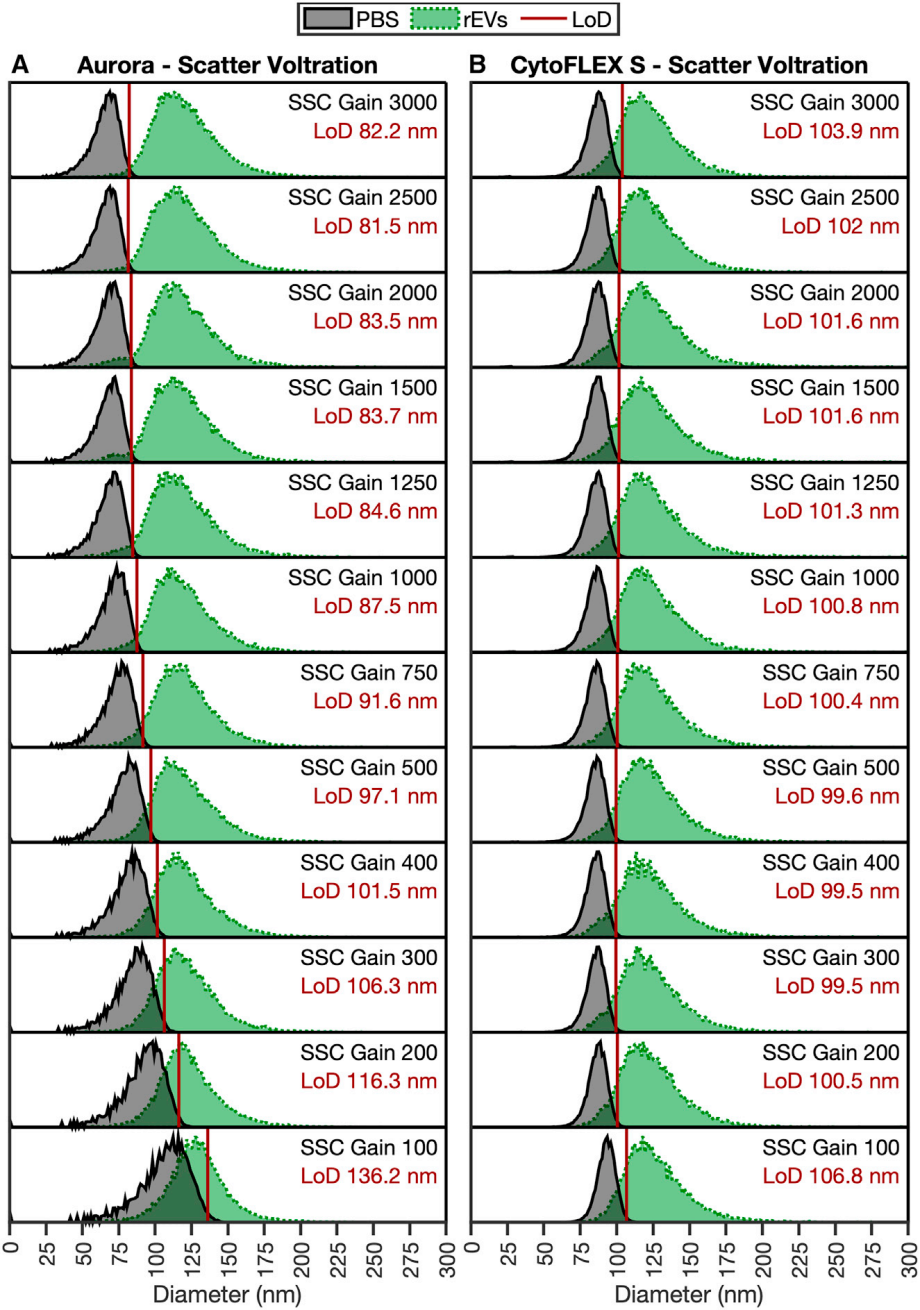
405 nm light scatter detector setting incrementation Probability density functions of buffer (gray) and rEVs (green) collected on the (A) Aurora and (B) CytoFLEX S at increasing detector settings. Probability density functions are normalized to 1 at the modal point of each distribution. The LLoD (solid red line) is specified in nm in the top right of the distribution plot and is calculated as the 99^th^ percentile of the buffer control. rEVs data were acquired at ∼5 × 10^6^ particles mL^−1^ diluted in PBS. The optimal gain occurs at the lowest LoD. rEV diameter assumes a core-shell structure with a shell thickness of 5 nm, an RI of 1.486, and a core RI of 1.42.

**Figure 5:**
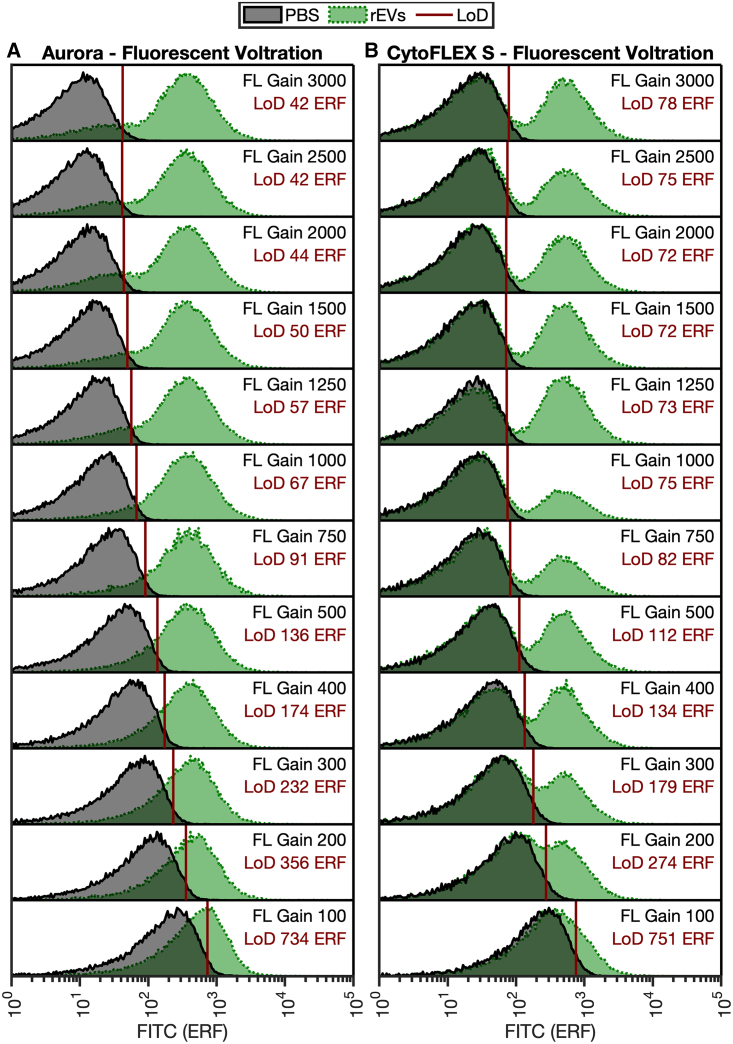
Fluorescence detector setting incrementation Probability density functions of buffer (gray) and rEVs (green) collected on the (A) Aurora and (B) CytoFLEX S at increasing detector settings. Probability density functions are normalized to 1 at the modal point of each distribution. The LLoD (solid red line) is specified in FITC ERF in the top right of the distribution plot and is calculated as the 99^th^ percentile of the buffer control. rEVs data were acquired at ∼5 × 10^6^ particles mL^−1^ diluted in PBS. The optimal gain occurs at the lowest LoD.

To validate the utility of the SPOT pipeline for biological particles, detector setting incrementation (DSI) using reference EVs was acquired across light scatter (Figure 4) and fluorescent (Figure 5) detector settings on two different instrument platforms. Despite both cytometers using APD detectors, there are notable differences in the results. The change in sensitivity was larger between settings on the Aurora (Figure 4A) than the CytoFLEX S (Figure 4B), with a total change in sensitivity from least to most sensitive being 136.2 to 81.5 nm, whereas the CytoFLEX S had a total range of 106.8 to 99.5 nm. Notably, in both cases, the most sensitive settings light scatter detectors did not have the highest detector gain. The light scatter detector settings derived as optimal using the SPOT pipeline are in agreement with the EV validation in Figures 3E and 3F, whereby peak sensitivity for the Aurora platform was obtained at a detector gain setting of 2,500 and the CytoFLEX S at 400.

In the case of fluorescence detector settings, sensitivity increased, due to the lower LoD decreasing from 734 to 42 FITC equivalent reference fluorophore (ERF), with peak sensitivity (lowest LoD) at a setting of 2,500. On the CytoFLEX S, sensitivity increased from 754 to 72 ERF. The derived detector settings using the SPOT pipeline and validation with EVs are in agreement. As indicated by bead-based detector setting analysis (Figure 3D), the highest gain on the CytoFLEX S does depreciate the sensitivity of the signal from peak sensitivity by ∼10%. These results indicate that the use of opto-electronic noise and the developed SPOT pipeline are capable of identifying optimal detector settings for small-particle analysis without the use of biological samples (Figures 3C–3F). The derivation of these settings is multi-factorial, and at a minimum, the SPOT pipeline should be repeated when any changes are made to the system’s electronics, fluidics, or optics, i.e., after a preventative maintenance visit. Periodic implementation is, however, recommended to ensure flow cytometer performance consistency over time.

### Optimized instrument configuration on EV detection

**Figure 6:**
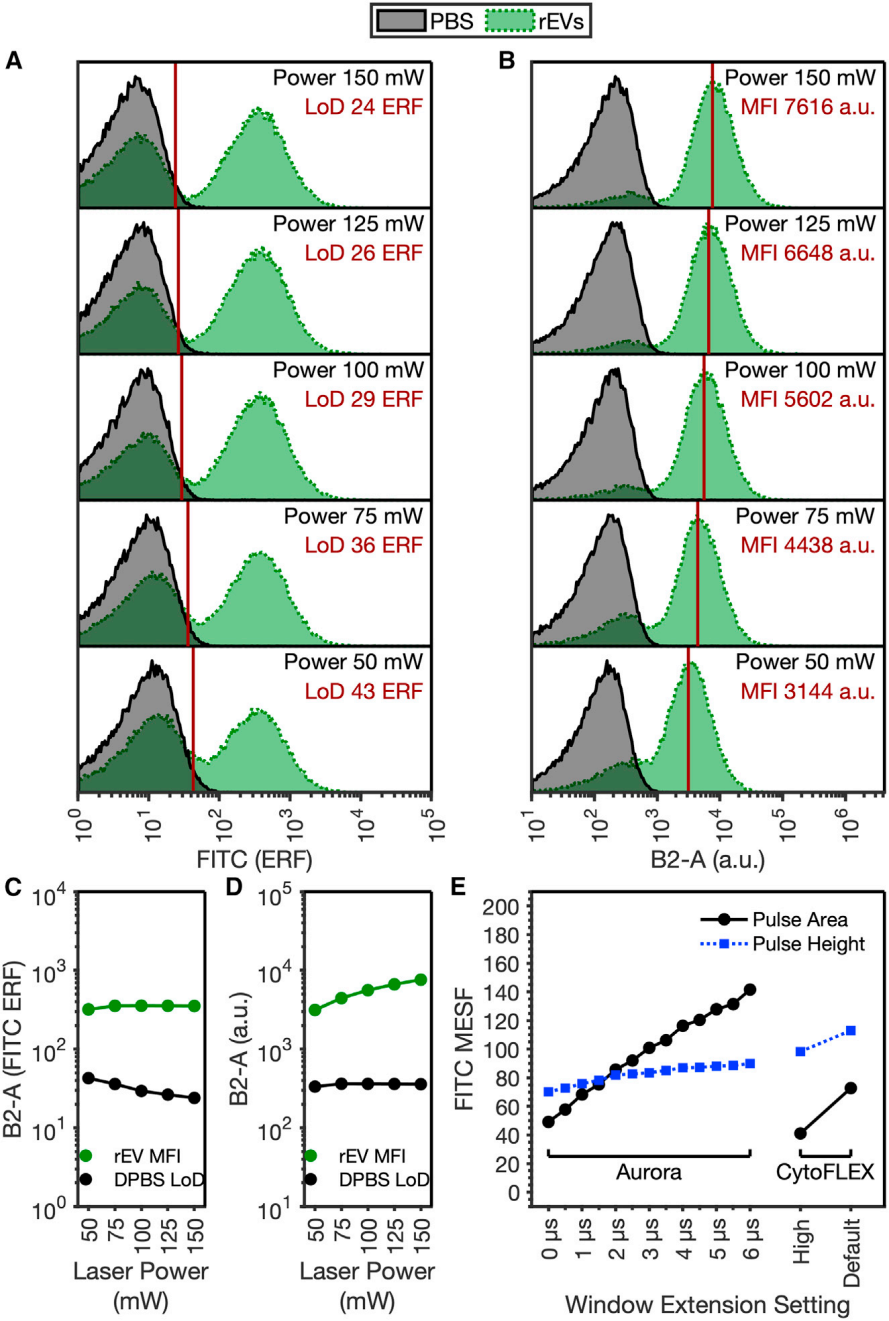
Laser power and window extension optimization (A and B) Probability density function overlays of PBS and rEV populations at varying laser powers on the Aurora platform in (A) FITC ERF units and (B) arbitrary units. (C and D) Summary statistics of PBS and rEV populations at varying laser powers on the Aurora platform in (C) FITC ERF units and (D) arbitrary units. (E) LLoD of the area (black dots) and height statistics (blue dots) on Aurora and CytoFLEX S platforms with varying window extension settings. Window extension settings were altered in 1-μs increments on the Aurora from 0 to 6 μs, while the CytoFLEX S platform was compared using “default” and “high acquisition mode,” as manual adjustments to window extensions are not supported.

**Figure 7:**
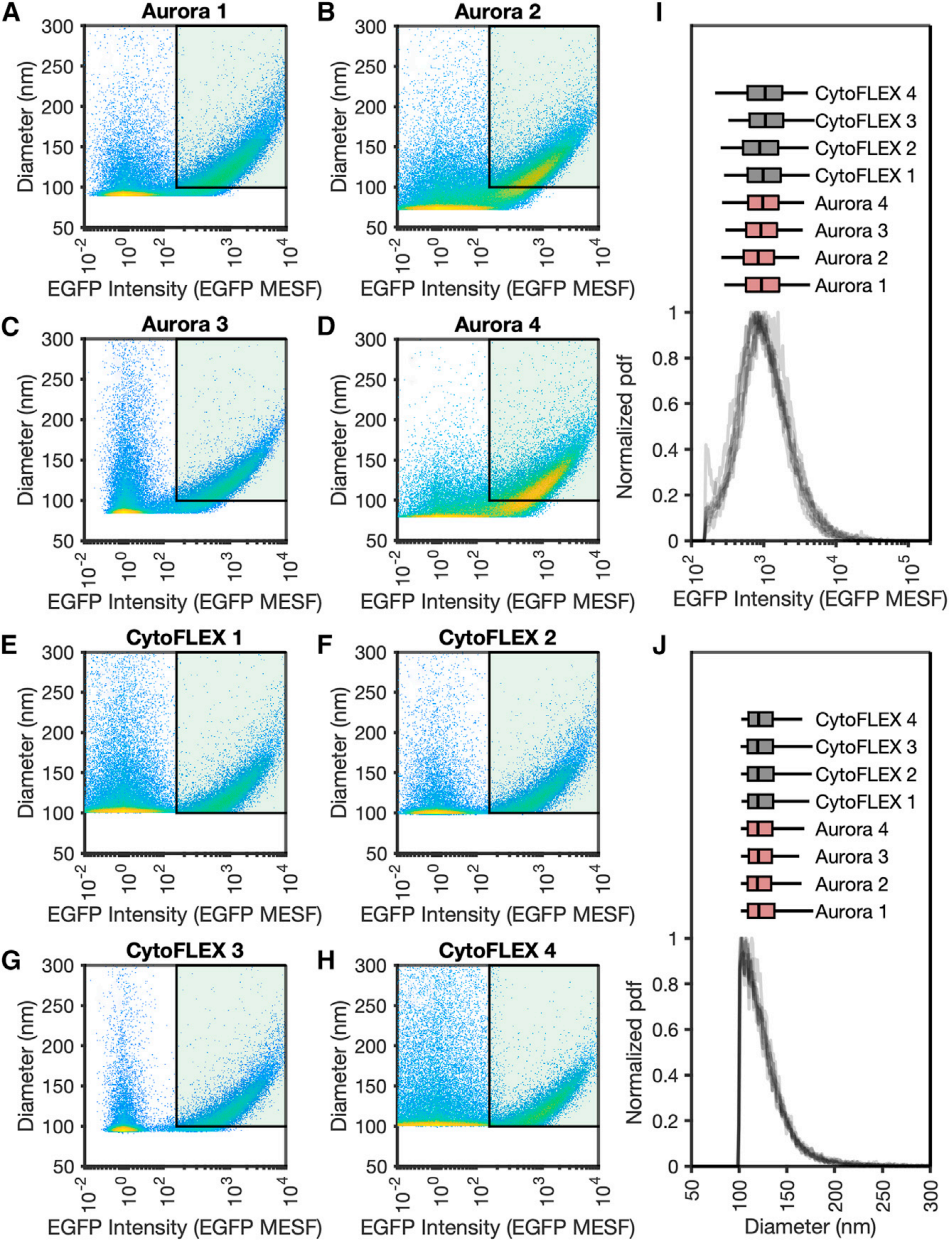
Cross-platform standardization (A–H) rEVs were acquired at FCM_PASS_ DSI derived settings on four Aurora (A–D) and four CytoFLEX S (E–H) cytometers. A rEV gate (green box) was drawn that was based on the least sensitive EGFP MESF and diameter detectable by the cohort of instruments. (I and J) Gated rEVs were overlaid for EGFP intensity (I) and diameter (J). Boxplots using the 5^th^, 25^th^, 50^th^, 75^th^, and 95^th^ percentiles of each distribution were plotted for each individual cytometer above the distributions.

Beyond detector settings, further modifications can be made to potentially increase the detection sensitivity of flow cytometers for signal quantification. These include the laser powers and window extensions. The effect of laser power on rEV detection was measured by increasing the power by 25 mW from 50 to 150 mW using optimal detector settings derived from the FCM_PASS_ DSI protocol (Figures 6A–6D). When calibrated units are utilized, the sensitivity increased from 43 to 24 ERF (Figure 6A). When visualized using a.u. (Figure 6B), the small gain in sensitivity from 100 to 150 mW is difficult to visualize, as both the noise and rEV populations are increasing with laser power. Figure 6C shows that, while the median fluorescence intensity of the rEV population is maintained, the signal-to-noise ratio continues to increase, signified by the linear reduction in the DPBS LoD population. Furthermore, when observing the median rEV statistics and the DPBS LoD (Figure 7D), the increase in rEV signal forms a hyperbolic response that is beginning to plateau, while the LoD linearly increases. This indicates that further increases in laser power will result in a diminished signal-to-noise ratio, as, eventually, the EGFP will saturate and be unable to emit anymore photons. Laser power versus fluorescence intensity can also be viewed on a linear plot in Figure S1.

Electronic acquisition settings, as well as detector and laser settings, can impact the sensitivity of the instrument. Most flow cytometers by default have a window extension (WE) of ∼5 μs. The WE is designed for cellular analysis and can increase the sensitivity for events that are larger than the laser beam height. The laser beam height on Aurora and CytoFLEX S platforms is ∼5 μm and is therefore far larger than most biological EPs. By decreasing the WE from 6 to 0 μs, sensitivity increased based on the fluorescence area statistic on the Aurora by 2.9-fold from 142 to 49 FITC ERF for rEVs. When using the fluorescence height statistic, the change in lower LoD (LLoD) was not as pronounced, decreasing from 90 to 70 FITC ERF, a 1.3-fold increase in sensitivity. Similarly, switching from “default” to “high acquisition mode” on the CytoFLEX S increased fluorescence area sensitivity 1.8-fold from 73 to 41 FITC MESF, while the height sensitivity increased from 113 to 98 FITC ERF (Figure 6E). In the case of both cytometers, when WE settings were optimized for small-particle detection, the fluorescence area statistic resulted in a higher sensitivity value than the height statistic.

### qFCM enables concordance in cross-platform interlaboratory small-particle studies

To investigate the utility of the FCM_PASS_ DSI protocol developed and the reproducibility of biological data acquired when optical signals were calibrated, a small cross-platform study was undertaken with four Aurora (Figures 7A–7D) and four CytoFLEX S (Figures 7E–7H) platforms. In all cases, when instruments were optimized for small-particle analysis, the EGFP-tagged rEV population was detectable. These instruments ranged in fluorescence sensitivity from 29 to 155 EGFP MESF and light scatter sensitivity from 72 to 100 nm for EGFP-tagged rEVs (modeled with a shell thickness of 5 nm RI of 1.486 at 405 nm and a core RI of 1.42 at 405 nm). To investigate the concordance of data in a fair comparison, the rEV gate was drawn using the sensitivity of the least sensitive instruments. Events inside a gate with a diameter of 100–300 nm and an EGFP MESF intensity of 155 to 2 × 10^5^ were compared by fluorescence (Figure 7I) and light scatter (Figure 7J). The median a.u. comparisons of data within the same gate resulted in a statistic ranging from 3,817 to 10,482 a.u. (175%) for fluorescence and from 3,114 to 10,858 a.u. (249%) for light scatter parameters. The use of qFCM resulted in highly concordant populations, with median statistics ranging from 838 to 1,054 EGFP MESF (∼26%) and from 119.1 to 120.6 nm (∼1%) for fluorescence and light scatter, respectively.

While the use of fluorescently tagged, commercially available reference materials is ideal for developing and validating optimal instrument settings for small-particle analysis, the numbers of fluorescently tagged molecules are at relatively high densities compared to the abundance of typical protein targets. As we have demonstrated using qFCM, there are likely an average of 800–1,000 copies of EGFP per rEV. Most often, FCM is utilized to measure the presence of surface epitopes, which are commonly present with less than 100 copies per particle. Detecting small numbers of surface proteins can be challenging due to having very weak signals. Not only do instrument detection parameters need to be finely tuned, but antibody fluorescent conjugates and concentrations also need to be thoughtfully carried out.

When comparing staining of identical clones of anti-CD81 antibodies conjugated to APC, PE, or Pacific Blue, anti-CD81-Pacific Blue yielded the lowest stain index, with a peak stain index of 0.63 using 30-min incubation and 0.60 using an overnight incubation at 2 μg mL^−1^. Anti-CD81-PE and anti-CD81-APC with a 30-min incubation yielded peak stain indices of 2.00 and 2.18, respectively, at 1 μg mL^−1^. At a higher antibody concentration of 2 μg mL^−1^, the stain indices for both PE and APC conjugates were decreased to 1.83 due to the increase in unbound antibodies resulting in “swarm detection.” Both anti-CD81-PE and anti-CD81-APC stain indices increased when using overnight incubation, with peak indices of 3.12 for PE and 2.68 for APC. While the peak stain index for anti-CD81-PE was at 2 μg mL^−1^, for anti-CD81-APC, this was at 1 μg mL^−1^ before decreasing to 2.27 at 2 μg mL^−1^ (Figures S2 and S3).

## Discussion

In this work we have demonstrated the SPOT pipeline to derive the most sensitive settings for FCM applications where small, dim particle analysis is required. We have demonstrated that the utilization of qFCM in assessing instrument detector sensitivity, laser powers, WEs, and pulse statistics enables both execution of SPOT to identify optimal small-particle FCM settings and reporting of data in standardized units. Furthermore, we have shown in a small cross-platform standardization study the ability of qFCM to generate highly concordant data, making cross-platform comparisons possible, facilitating the utility of commercially available instruments for the development of standardized assays. In addition to demonstrating a streamlined process for small-particle FCM instrument optimization, we have demonstrated the utility of this pipeline in the selection and optimization of conjugated antibody use for phenotyping of EVs. As well, we have validated the use of this approach to compare results across instruments, experiments, and institutions.

FCM_PASS_ was developed to be compatible with any commercial calibration reference materials for both fluorescence and light scattering calibration. Since its initial release, further improvements have been made to better support the needs of the field. The outputs of the FCM_PASS_ software now support automated export and completion of calibration materials, parameters, quality control, and hardware information for both MIFlowCyt and MIFlowCyt-EV reporting standards to enable transparent reporting in an ergonomic manner. The SPOT pipeline presented in this article provides a stepwise and streamlined way to leverage FCM_PASS_ tools to generate more robust and reproducible EV measurements.

While we have demonstrated the utility of commercially available instruments when optimized for small-particle detection, we have also demonstrated that these instruments are working close to their LoDs and beyond their intended specifications. Standard reference materials, software tools, and the methods presented herein are not only needed for researchers but also for manufacturers to improve the consistency and performance of instruments and instrument support, from manufacturing to benchtop performance.

### Limitations of the study

While the developed pipeline has been tested on conventional and spectral cytometers along with on avalanche photodiode, photomultiplier tubes and silicon photomultiplier detectors, the pipeline is not applicable to imaging cytometers. The use of calibration on any flow cytometry platform assumes that the system has a linear response to input intensity. Some older generation instruments utilizing analogue log amplifiers which can be non-linear in their signal:response may therefore may lack accuracy when calibrated.

## STAR★Methods

### Key resources table

**Table:** 

REAGENT or RESOURCE	SOURCE	IDENTIFIER
**Antibody**		

anti-CD81 APC	Biolegend	Cat 349509, Lot B340367, RRID:AB_2564021
anti-CD81 Pacific Blue	Biolegend	Cat 349515, Lot B349914, RRID:AB_2687126
anti-CD81 PE	Biolegend	Cat 349505, Lot B330215, RRID:AB_10645519

**Software and algorithms**		

MATLAB	Mathworks	https://www.mathworks.com/
FCM_PASS_	National Cancer Institute	https://nano.ccr.cancer.gov/fcmpass
Data analysis scripts	This paper	https://doi.org/10.6084/m9.figshare.22335835.v1

**Other**		

Aurora + ESP (V-B-Y-R), NIH	Cytek Biosciences	N/A
Aurora + ESP (UV-V-B-Y-R), Cytek	Cytek Biosciences	N/A
Aurora + ESP (UV-V-B-Y-R), uOttawa	Cytek Biosciences	N/A
Aurora + ESP (UV-V-B-Y-R), AFC	Cytek Biosciences	N/A
CytoFLEX (V-B-Y-R), NIH	Beckman Coulter	N/A
CytoFLEX (V-B-Y-R), AFC1	Beckman Coulter	N/A
CytoFLEX (V-B-Y-R), AFC2	Beckman Coulter	N/A
CytoFLEX (V-B-Y-R), AFC3	Beckman Coulter	N/A
DI water	Sigma Aldrich	Cat 270733
DPBS	Thermo Fisher Scientific	Cat. 270733
Exosome standards, fluorescent (rEVs)	Millipore Sigma	Cat. SAE0193, Lot 125377
FluoSpheres Carnboxylate Beads (100 nm)	Thermo Fisher Scientific	F8803
Low Protein Binding Tubes (1.5 mL)	Thermo Fisher Scientific	Cat. 90410
Low Protein Binding Tubes (0.5 mL)	Thermo Fisher Scientific	Cat. 88379
MESF Beads (APC)	Becton Dickinson	Cat. 626425, Lot 0273462
MESF Beads (BV421)	Becton Dickinson	Cat. 625508, Lot 026682
MESF Beads (FITC)	Bangs Laboratories	Cat. 555B, Lot 14610
MESF Beads (PE)	Becton Dickinson	Cat 340495, Lot 51753
NIST-traceable beads (81 nm)	Thermo Fisher Scientific	Cat. 3080A, Lot 228748
NIST-traceable beads (100 nm)	Thermo Fisher Scientific	Cat. 3100A, Lot. 204935
NIST-traceable beads (152 nm)	Thermo Fisher Scientific	Cat. 3150A, Lot. 202026
NIST-traceable beads (203 nm)	Thermo Fisher Scientific	Cat. 3200A, Lot. 205131
NIST-traceable beads (240 nm)	Thermo Fisher Scientific	Cat. 3240A, Lot. 226952
NIST-traceable beads (303 nm)	Thermo Fisher Scientific	Cat. 3300A, Lot. 204665
NIST-traceable beads (345 nm)	Thermo Fisher Scientific	Cat. 3350A, Lot. 199283
NIST-traceable beads (401 nm)	Thermo Fisher Scientific	Cat. 3400A, Lot. 203859
NIST-traceable beads (453 nm)	Thermo Fisher Scientific	Cat. 3450A, Lot. 204047
Protein LoBind Tubes (5 mL)	Eppendorf	Cat. 30122356
QbSure Beads	Cytek Biosciences	Cat B7-10005, Lot AF01
V-botton plates (96-well)	Evergreen	Cat. 222-8031-01V

### Resource availability

#### Lead contact

Requests for further information should be directed to the lead contact: Joshua Welsh, joadwe@outlook.com.

#### Materials availability

No unique reagents were generated in this study.

#### Data and code availability

(1) The code used to generate all figures and supplementary information in the manuscript can be found on FigShare: https://doi.org/10.6084/m9.figshare.22335835.v1.
(2) All data used in the paper can be found on the NanoFlow Repository at: https://genboree.org/nano-ui/manuscript/1753349353
(3) Any additional information required to reanalyze the data reported in this work paper is available from the lead contact upon request.

### Experimental model and subject details

#### Ethics statement

No human samples were utilized in this study. All reagents and reference materials utilized in this study are commercially available with information shared in the key resource table.

### Method details

#### Instrument characterization using SPOT pipeline

Instrument characterization is initially started by running the manufacturer’s daily QC procedure to ensure correct laser delays for downstream analysis, and to ensure detectors are within their normal working ranges as determined by the manufacturer, Figure 1. From here, the light scatter detector optimization is performed utilizing Methods S1. This process identifies the optimal light scatter detector settings by performing detector setting incrementation (DSI) analysis using a fluorescent trigger. DSI is a method where the same reference material is acquired multiple times, with each acquisition having an increasing detector setting, such as voltage or gain. Once found, the optimal light scatter detector settings are then used to determine the optimal trigger threshold settings. The optimal trigger settings are found by using a buffer only control and reducing the threshold until a stable event rate of 1000–1500 events s^−1^ is found. In a clean system, the majority of triggered events using the buffer only control will be from the opto-electronic noise population which is critical to identify for downstream fluorescence limit of detection. This population is distinct from sheath and sample debris in that it has a sharp increase in event rate once reached and can be triggered on any detector. Optimal fluorescent detector settings and limit of detection are next found by performing DSI analysis using fluorescent multi-peak beads and using the light scatter trigger on the opto-electronic noise previously identified (Methods S2). Finally, the sensitivity of the light scatter detector is quantitated by acquiring NIST-traceable beads and performing light scatter calibration (Methods S3). Protocols used to validate the SPOT pipeline derived settings using recombinant extracellular vesicles (rEVs) can be found for light scatter detector settings (Methods S4), fluorescence detector settings and sensitivity (Methods S5), light scatter sensitivity (Methods S6), and antibody staining (Methods S7). All resources can be found in the supplemental information and online.^16^

#### Flow cytometry

FCM fluorescence and light scattering settings were optimized and calibrated utilizing FCM_PASS_ (v4.2, https://nano.ccr.cancer.gov/fcmpass). To demonstrate the consistency of optimal gain derivation on the CytoFLEX platform that showed less of a clear improvement than the Aurora platform, the optimal gain characteristics were derived fives and plotted, Figure 1. Calibration reference materials, acquisition files, settings, QC plots, MIFlowCyt and MIFlowCyt-EV reports can be found in https://genboree.org/nano-ui/manuscript/1753349353.^12^^,^^17^^,^^18^^,^^19^ All Aurora (Cytek Bioscience) instruments had an enhanced small particle detection (ESP) module on the 405 nm laser. Three Aurora’s were configured with 50 mW 355, 405, 488, 561, and 640 nm, One Aurora was configured with 50 mW 405 and 640 nm lasers, 150 mW 488 and 561 nm lasers, and a quantiFlash LED pulser (APE) and electronically integrated FSC LED pulser trigger.^20^ All CytoFLEX S (Beckman Coulter) platforms were equipment with 405, 488, 561, 640 nm lasers. Flow cytometers were calibrated using QbSure beads cross-calibrated to molecules of equivalent soluble fluorophore (MESF) units using FCM_PASS_ on the NIH CytoFLEX and Aurora flow cytometers. The assigned bead values were used across all other cytometers of the same platform. Aurora and CytoFLEX cross-calibrations can be found in Figure 4.

#### Cross-calibration of ERF to MESF

Currently, commercially available fluorescence calibration reference materials are available in MESF and equivalent reference fluorophore (ERF) units. An understanding of the differences between MESF and ERF units is critical when attempting to make comparisons across platforms and assays. In the context of FCM applications, a detector scale calibrated to MESF units is quantifying a signal from a fluorophore whose spectroscopic properties match the fluorophore being quantified e.g., a detector’s intensity scale is calibrated into molecules of phycoerythrin (PE) and the particles being phenotyped are labeled with a PE-conjugated antibody. A detector scale calibrated to ERF units is quantifying a signal whose spectroscopic properties do not match the fluorophore being quantified. Figure S5 demonstrates how ERF and MESF units relate and can be made interoperable by factoring in spectroscopic data. Figure S5A shows a detector’s intensity scale calibrated into molecules of fluorescein Isothiocyanate (FITC) and particles fluorescently tagged with enhanced green fluorescent protein (EGFP). When EGFP-tagged rEVs are detected between two platforms with different fluorescent detection filters, the intensity of the rEV population detected by the CytoFLEX is approximately 23% higher than the Aurora, with median FITC ERF intensities of 505 and 412. Currently, FITC reference materials are readily available, those for EGFP are not.

When observing the spectra of FITC and EGFP, Figure S15B, it is evident that the emission properties of the two fluorophores differ in their region of excitation maximum, with EGFP at ∼508 nm and FITC at ∼525 nm. Furthermore, the CytoFLEX S collection filter has a larger bandwidth (525/40) than the Aurora (525.5/17). The result of differing collection bandwidths is a difference in brightness collected with the CytoFLEX collecting ∼2x more photons than the Aurora for FITC and ∼2.5x more photons from EGFP. The difference in collection bandwidth not only affects the quantity of light collected but also the ratio of FITC molecules to GFP molecules, Figure S5C. One molecule of FITC on the CytoFLEX S is equivalent to ∼0.57 molecules of EGFP, whereas on the Aurora one molecule of FITC is equivalent to ∼0.47 molecules of EGFP, Figure S5D. By accounting for fluorophore brightness and collection filter bandwidths, it is possible to approximately convert FITC ERF to EGFP MESF by dividing the ERF values by 0.57 on the CytoFLEX S and 0.47 on the Aurora platforms, Figure S5E. This conversion to MESF allows for direct comparisons of fluorescent information between the two platforms with the 23% difference in FITC ERF units reduced to 1% difference in EGFP MESF units. The median rEV intensities now being 882 GFP MESF for the CytoFLEX and 875 GFP MESF for the Aurora.

The ability to make MESF comparisons is therefore not limited to the availability of fluorescence reference materials but can be approximated by accounting for differences in fluorophore spectroscopic properties using readily available fluorophore spectroscopic information and the collection filters used within a flow cytometer. GFP MESF throughout this work utilize this normalization method.

#### Reference material preparation

Commercial (rEVs) (Millipore Sigma, Cat. SAE0193) were resuspended from lyophilization according to manufacturer recommendations in 100 μL deionized water (Sigma Aldrich, Cat. 270733) and reverse pipetted. Serial dilutions of resuspended rEVs in DPBS, Figure S6, were used to confirm the concentration of ∼5 x10^6^ particles mL^−1^ resulted in single particle detection that was used for all downstream analyses.

#### rEV immunophenotyping

Three identical clones (TAPA-1) of CD81 antibody conjugated to three different fluorophores were tested: pacific blue, phycoerythrin (PE), and allophycocyanin (APC). Antibodies were incubated from 6.25x10^−2^ to 2 μg mL^−1^ and compared at 30 min and overnight incubations (∼12 h). Buffer + antibody controls, unstained rEVs, and antibody-labelled rEVs were acquired. A stain index, calculated using qFCM EGFP-positive rEVs data incorporating the buffer with antibody control in lieu of a negative rEV population, was used to compared labeling efficacy while accounting for the fluorescence contribution from unbound antibodies, along with ERF and MESF unit calibrations, Figures S2 and S3.

### Quantification and statistical analysis

#### Statistical analysis

Data analyses were performed in MATLAB (Mathworks Inc, v2022b). All manuscript data and MATLAB data analysis and figure generation scripts can be accessed at the repository link: https://doi.org/10.6084/m9.figshare.22335835.v1. Unless otherwise stated all stated fluorescent intensities are based on the median statistic due to the non-parametric nature of the fluorescent intensity. Unless otherwise stated the lower LoD was obtained using the 99^th^ percentile of the background noise (DPBS) population. Normality testing of noise population utilized a MATLAB’s default Kruskal-Wallis, skewness and kurtosis functions. Stain indices were calculated using the following formula:

$$\frac{\left(rEV\;median\;–\;buffer+antibody\;median\right)}{95^{th}percentile\;of\;buffer+antibody}\text{.}$$

## Supplemental information

Document S1. Figures S1–S6, Table S1, and supplemental methods

Document S2. Article plus supplemental information
