# Supplementary material for: Quantitative flow cytometry enables end-to-end optimization of cross-platform extracellular vesicle studies
Source: Cell Rep Methods. 2023 Dec 18;3(12):100664. doi: 10.1016/j.crmeth.2023.100664 (PMC10753385; doi:10.1016/j.crmeth.2023.100664)
Supplement: Document S1. Figures S1–S6, Table S1, and supplemental methods [file mmc1.pdf]

Cell Reports Methods, Volume *3*

Supplemental information

Quantitative flow cytometry enables end-to-end optimization of cross-platform extracellular vesicle studies

Sean Cook, Vera A. Tang, Joanne Lannigan, Jennifer C. Jones, and Joshua A. Welsh

Supporting Information for

Quantitative flow cytometry (qFCM) enables end-to-end optimization of cross-platform extracellular vesicle studies.

Sean Cook, Vera A. Tang, Joanne Lannigan, Jennifer C. Jones, Joshua A. Welsh

Lead author: Joshua A. Welsh.
E-mail: joadwe@outlook.com

This PDF file includes:

Figs. S1 to S6
Table S1

Contents

1 Flow cytometer noise characterization methods **3**

2 CytoFLEX light scatter optimal gain derivation laser power and rEV detection **4**

3 Recombinant EV immunophentyping **5**

4 Rainbow bead cross-calibration **7**

5 FITC ERF to GFP MESF **8**

6 Recombinant EV serial dilution **9**

7 Supplementary Methods **10**
A Methods S1: Flow cytometer light Scatter detector setting incrementation, related to Figure 1 10
B Methods S2: Flow cytometer fluorescence detector setting incrementation, related to Figure 1 18
C Methods S3: Flow cytometer light scatter calibration, related to Figure 1 26
D Methods S4: Flow cytometer rEV serial dilution, related to Figure 1 34
E Methods S5: Flow cytometer rEV light scatter detector setting incrementation, related to Figure 1 43
F Methods S6: Flow cytometer rEV fluorescence detector setting incrementation, related to Figure 1 51
G Methods S7: Flow cytometer rEV immunophenotyping, related to Figure 1 58

1. Flow cytometer noise characterization methods

Name	Lower LoD (FITC ERF)	Normality (p-value)	Skewness	Kurtosis
QbSure	421	<0.001	0.1036	2.8962
LED Pulser	212	<0.001	0.1578	3.2118
100 nm	58	<0.001	0.4456	3.6074
81 nm	47	<0.001	0.4292	3.5076
DPBS	27	<0.001	0.6572	4.2718

Table S1. Comparison of reference materials available for assessing fluorescence lower limits of detection, derived from Figure 3B.

2. CytoFLEX light scatter optimal gain derivation laser power and rEV detection

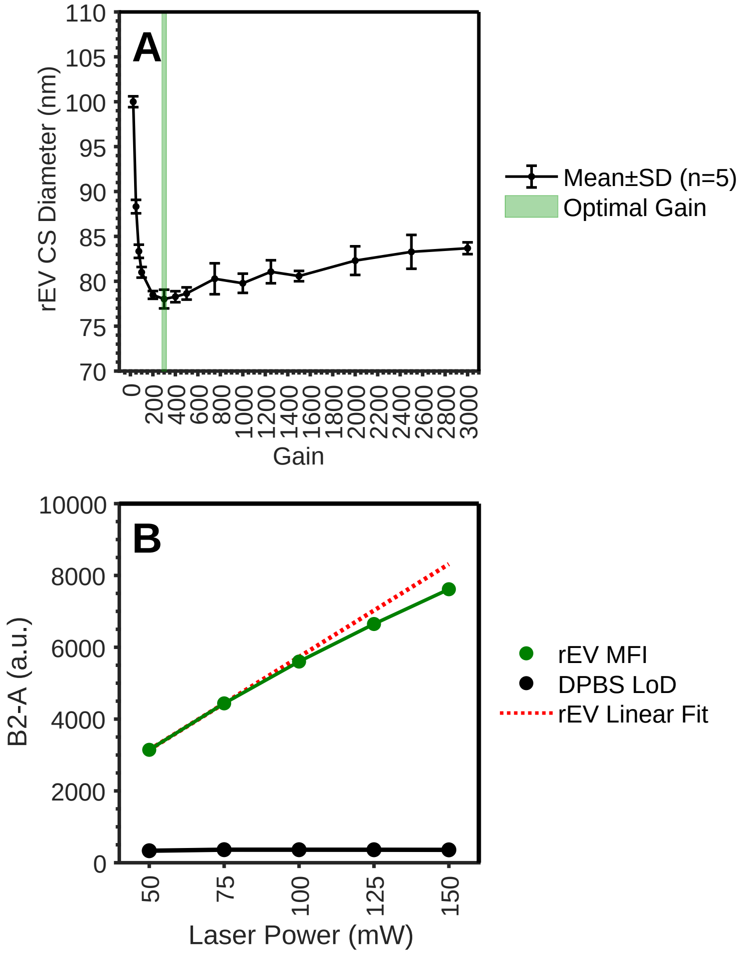

Fig. S1. Stability of SPOT pipeline, as outlined in Figure 3. A) CytoFLEX LX light scatter detector setting incrementation showing the mean and standard deviation of the derived limit of detection from the SPOT pipeline. Optimal gain derived by the SPOT pipeline is highlighted in green. B) Median fluorescent intensity (MFI) of recombinant EVs (green) and 95th percentile of DPBS, which is the limit of detection (LoD).

3. Recombinant EV immunophentyping

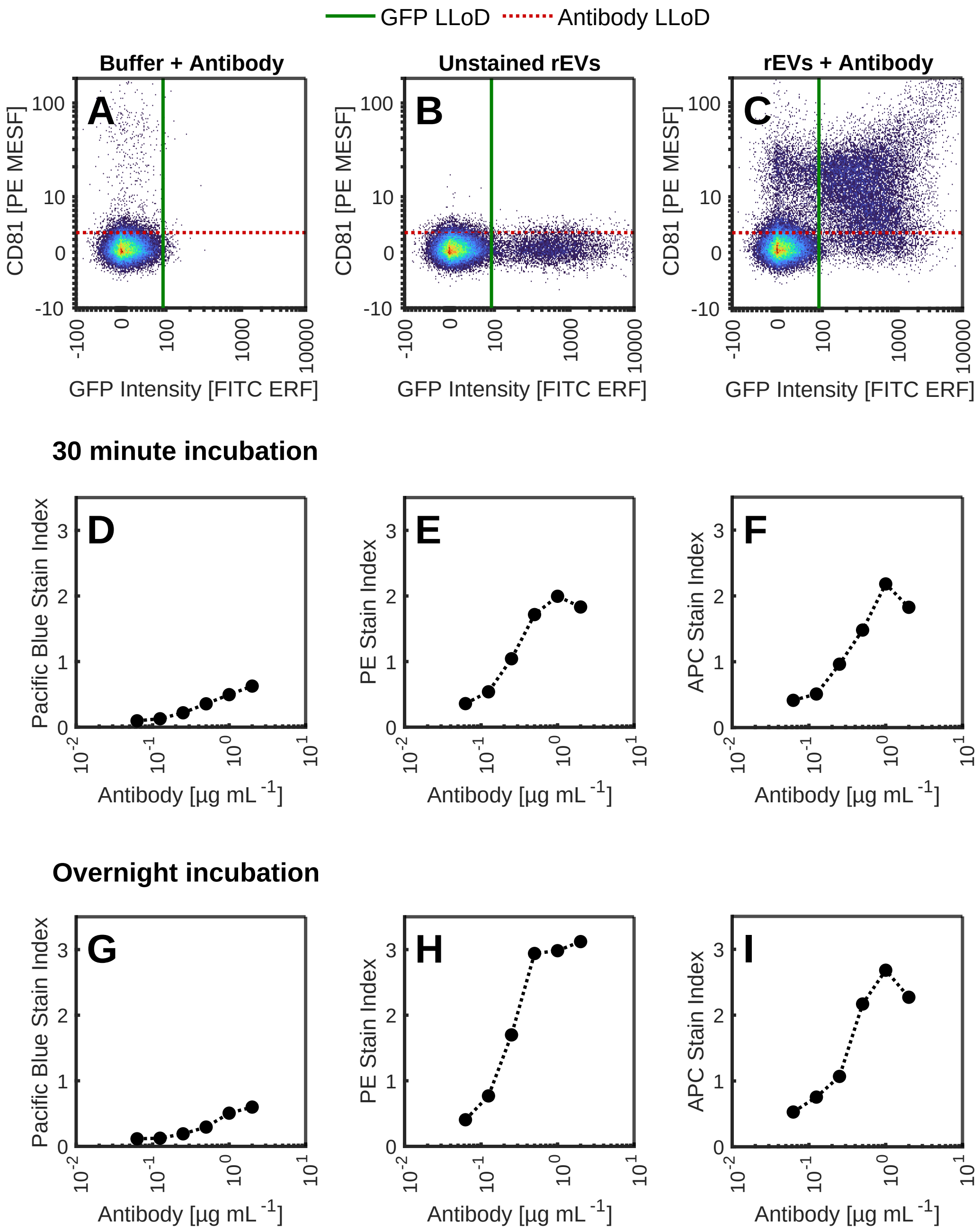

Fig. S2. Ability to phenotype at optimized settings, as derived from Figure 3. Optimization of antibody staining with stain index. rEV were stained with anti-CD81-PE antibody with buffer + antibody control (A), unstained rEV population (B), and 2 µg mL-1 anti-CD81-PE-stained rEV population incubated overnight at 2 µg mL-1 (C), before a 200-fold dilution for acquisition. Stain index of stained rEVs with anti-CD81-Pacific Blue (D, G), PE (E, H), and APC (F, I) at 30 minutes and overnight, respectively.

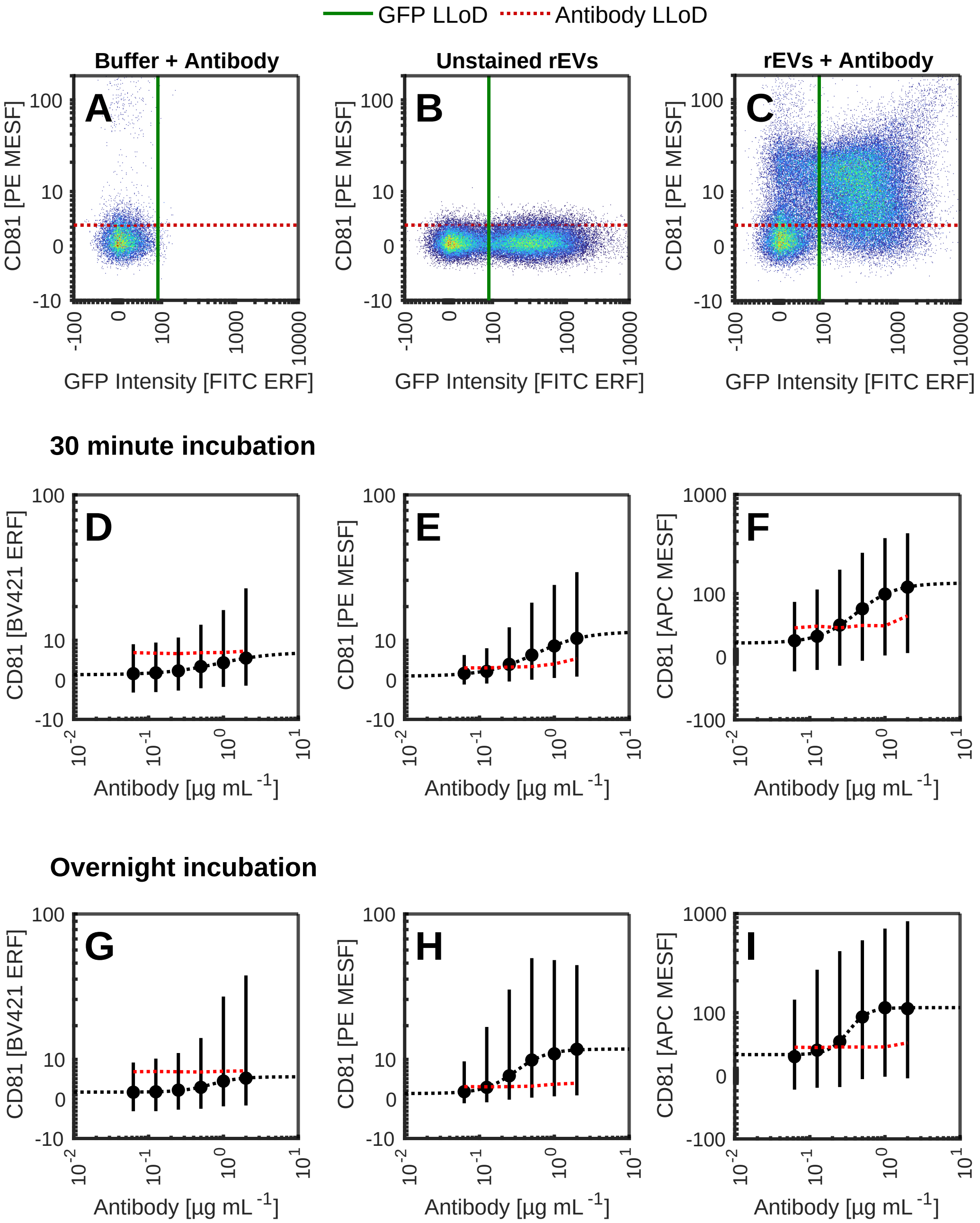

Fig. S3. Ability to phenotype at optimized settings, as derived from Figure 3. Optimization of antibody staining with calibrated units. Acquisition of buffer with antibody incubated overnight at 2 µg mL-1 anti-CD81-PE before a 200-fold dilution for acquisition (A), unstained rEV population (B), and 2 µg mL-1 anti-CD81-PE-stained rEV population incubated overnight (C). Calibrated intensity of stained rEVs with anti-CD81-Pacific Blue (D, G), PE (E, H), and APC (F, I) at 30 minutes and overnight, respectively. The limit of detection (95th percentile) using the buffer with antibody control is denoted by the dotted red line.

4. Rainbow bead cross-calibration

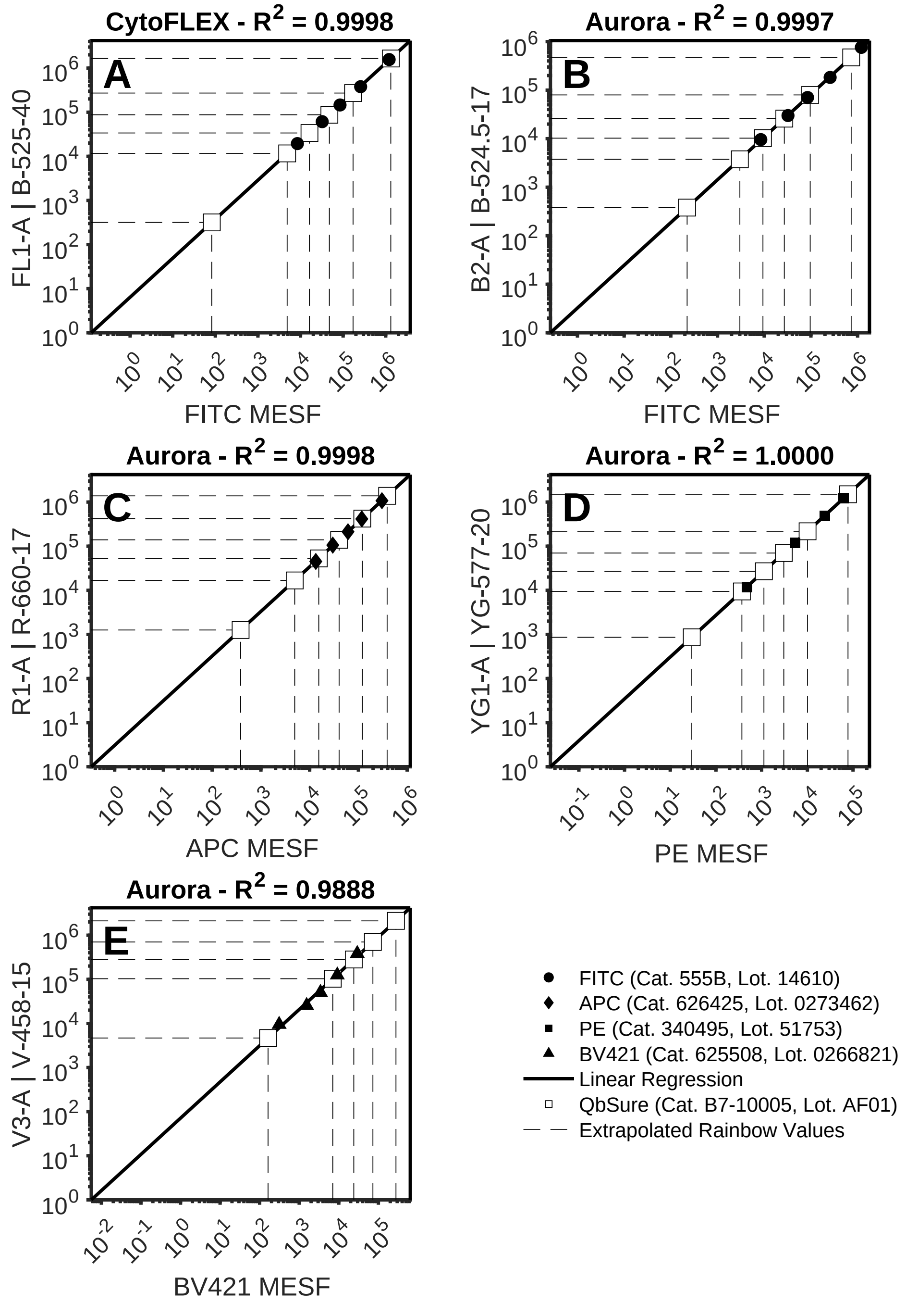

Fig. S4. Cross-calibration of QbSure beads to ERF units using MESF beads on the CytoFLEX and Aurora flow cytometers, as used in Figures 2-7

5. FITC ERF to GFP MESF

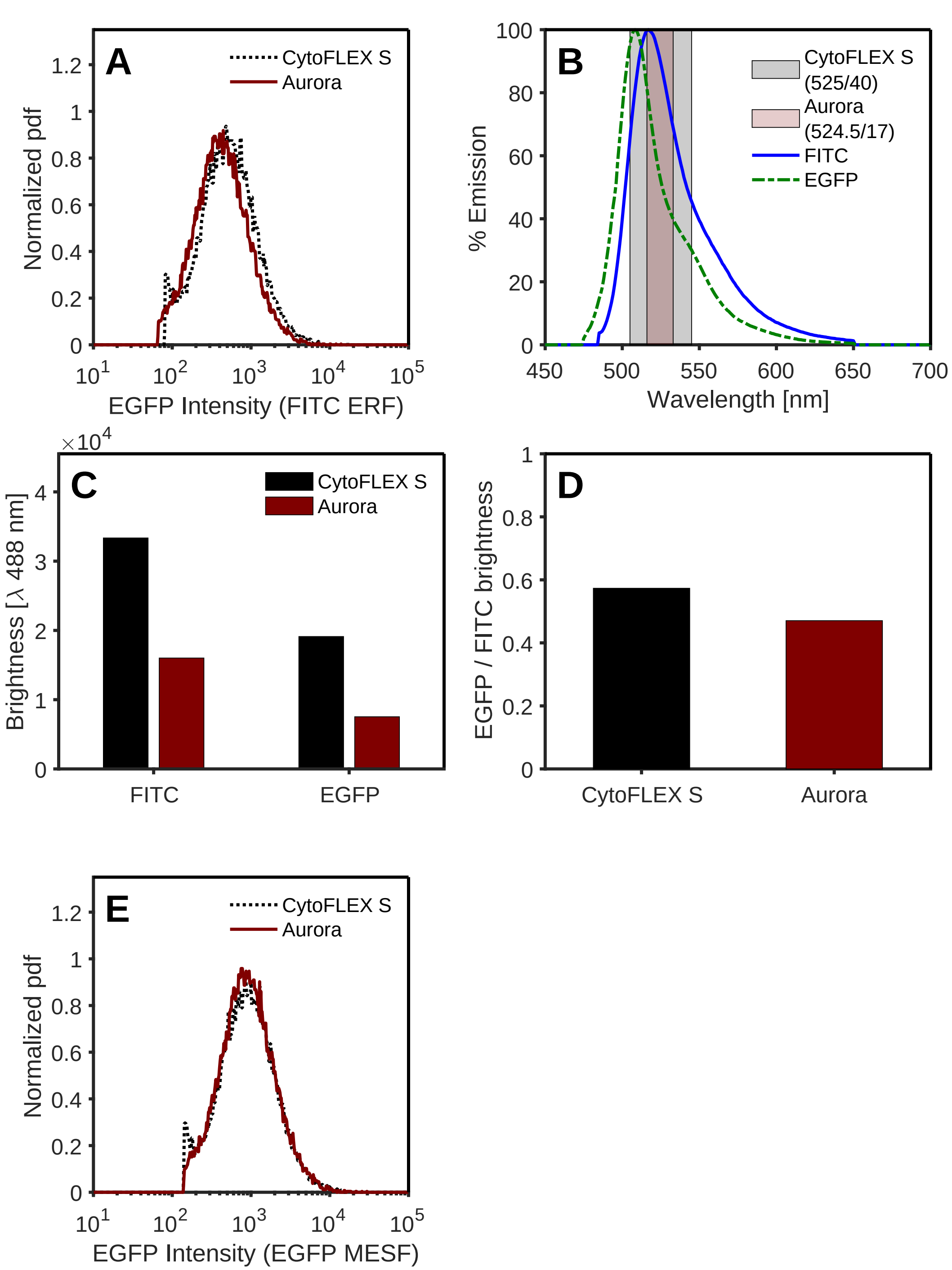

Fig. S5. Utilization of cross-calibration, as used in Figures 2-7. A) rEV population calibrated to FITC ERF units on the CytoFLEX S (dashed blacked line) and Aurora (solid red line) platforms. B) Normalized emission spectra for fluorescein isothiocyanate (FITC) (solid blue line) and enhanced green fluorescent protein (EGFP) (dashed dotted green line). The collection bandwidths for the CytoFLEX S (grey) and Aurora (red) as overlaid on the emission spectra. C) Collected light from FITC and EGFP fluorophores on the CytoFLEX S (black) with a bandpass of 525/40 nm and the Aurora (red) with a bandpass of 524.5/17 nm. Brightness of FITC assumes extinction coefficient of 75000 M-1 cm-1, quantum yield of 0.92, and excitation of 77.33% at 488 nm. Brightness of EGFP assumes extinction coefficient of 55900M-1 cm-1, quantum yield of 0.6, and excitation of 99.87% at 488 nm. D) Ratio of EGFP to FITC brightness between CytoFLEX (red) and Aurora (black) account for relative brightness and collection bandwidth. E) rEV population calibrated to EGFP MESF units on the CytoFLEX S (dashed blacked line) and Aurora (solid red line).

6. Recombinant EV serial dilution

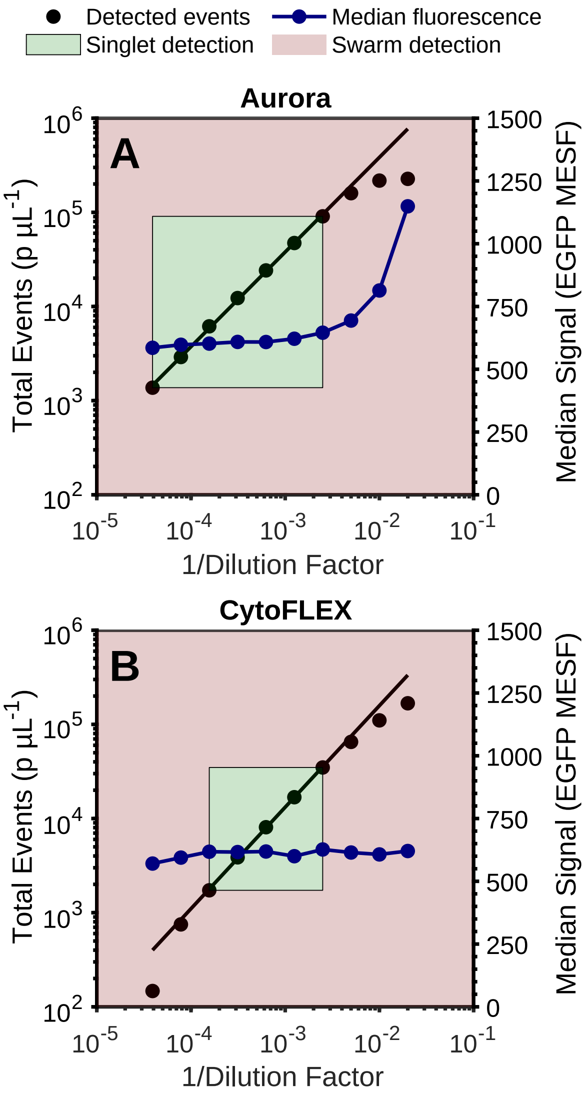

Fig. S6. rEV serial dilution on Cytek Aurora (A) and Beckman Coulter CytoFLEX S (B), as used in Figures 2, 4-7. Representative serial dilution of rEV stock on Aurora platform. Measured (black dots) and robust regression (solid black line) concentration of rEVs plotted on left y axis. Measured median fluorescent intensity in calibrated units shown on right y axis. (blue dots). Green region represents reliable single particle detection where there is a linear decrease in particle number and stable median fluorescent intensity. Red region represents coincidence detection.

7. Supplementary Methods

A. Methods S1: Flow cytometer light Scatter detector setting incrementation, related to Figure 1.

Resource 1: Scatter Detector Setting Incrementation for FCMPASS

Forked from Flow Cytometer Fluorescence Voltration for FCMPASS

In 1 collection

Sean M Cook[1], Jennifer Jones[1], Joshua A Welsh[1]

[1]Translataional Nanobiology Section, Laboratory of Pathology, Center for Cancer Research, National Cancer Institute, National Institutes of Health

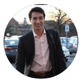

Sean M Cook

Protocol Info: Sean M Cook, Jennifer Jones, Joshua A Welsh . Resource 1: Scatter Detector Setting Incrementation for FCMPASS. **protocols.io** https://protocols.io/view/resource-1-scatter-detector-setting-incrementation-cnjcvciw

Created: Jan 30, 2023

Last Modified: Mar 25, 2023

PROTOCOL integer ID: 76100

DISCLAIMER

This protocol summarizes key steps for a specific type of method, which is one of a collection of methods and assays used for EV analysis in the NCI Translational Nanobiology Section at the time of submission of this protocol. Appropriate use of this protocol requires careful, cohesive integration with other methods for EV production, isolation, and characterization.

ABSTRACT

Protocol to perform flow cytometer voltration to identify optimal detector settings for small particle analysis. Data acquired from this protocol are compatible with semi-automated analysis tools built into FCMPASS software.

MATERIALS

FluoSpheres Carnboxylate **Thermo Fisher Scientific Catalog #F8803** , referred to as 'FluoSpheres'

5 mL Round-bottom tube **Corning Catalog #352052** , referred to as 'FACS tubes'

DPBS **Thermo Fisher Scientific Catalog #14190144** , referred to as 'DPBS'

QbSure **Cytek Catalog #B7-10005** , referred to as 'QbSure'

Sample preparation

10s

1 Vortex FluoSpheres bottle on a high setting for 00:00:05 . 5s

Note

This stock bottle is 2% solids containing 110 nm beads, equal to ~2.73E13 / mL

Note

This method in principle is compatible with any fluorescent nanosphere that is bright enough to be fully resolved when using a fluorescent trigger.

2 Create 3 mL of 5E6 p/mL solution of FluoSpheres in a FACS tube.

Note

An observation from our protocol development is that using low protein binding tubes for this step may negatively impact the efficacy of this protocol as it can result in excess unbound fluorophore from the beads increasing background noise leading to excessive event rate.

2.1 Pipette 2998.90 µL of DPBS into a FACS tube. Label this tube '1E10 Intermediate'.

2.2 Pipette 1.10 µL stock FluoSpheres into the tube labelled '1E10 Intermediate' . Reverse pipette the tube to mix.

2.3 Pipette 990 µL of DPBS into a FACS tube. Label this tube '1E8 Intermediate'. Pipette 10 µL "1E10 Intermediate" into the tube. Reverse pipette the tube to mix.

2.4 Pipette 2850 µL of DPBS into a FACS tube. Label this tube 'Beads. Pipette 150 µL "1E8 Intermediate" into the tube. Reverse pipette the tube to mix. This should result in a concentration of ~5E6 p/mL of FluoSpheres, and this tube will be used for acquisition.

3 Pipette 500 µL of DPBS into a FACS tube. Label this tube 'DPBS'.

Cytometer Setup

4 Ensure cytometer is clean and that -Height and -Area statistics are set to be collected on all parameters and that all parameters are on.

5 On the Cytek Aurora, set window extension to 0. On the CytoFLEX platform turn on 'High Acquisition Mode'.

> **Note**
>
> To set the window extension to 0 on the Cytek Aurora, navigate to the 'Lasers' tab under the sample acquisition settings and set the window extension to 0.
>
> To change the acquisition mode on the CytoFLEX S, click on the 'Advanced' menu on the top-of-the-screen ribbon. Next click on 'Event Rate Settings' menu, change the acquisition mode to 'High', and press 'Ok'.

6 Create a histogram plot with the FITC height parameter (CytoFLEX: FL1-H | B-525-40, Aurora: B2 | B-524.5-17) on the X-Axis and make sure it is plotted on a log-scale.

7 Create a histogram plot with (405 nm) violet SSC-A (CytoFLEX: SSC_1-A, Aurora: SSC-A) on the X-Axis and make sure it is plotted on a log-scale.

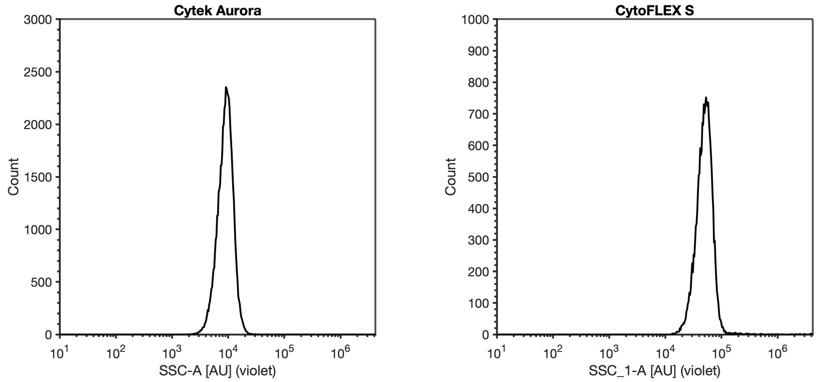

Example of FluoSpheres acquired on Cytek Aurora and CytoFLEX S. Data has been gated to remove the noise population.

8 Set the cytometer triggering threshold to the FITC parameter (CytoFLEX: FL1 | B-525-40, Aurora: B2 | B-524.5-17) . **All samples should be acquired with the lowest flow rate, typically**

~10-15 µL min^{-1}.

Note

Cytometer Voltage/Gain and threshold settings are subjective due to their dependency on alignment, and the scatter filters in place, amongst other variables. The following are guide values to start with and may need adjustment for optimal acquisition.

Beckman Coulter, CytoFLEX [405 nm OD0 filter, 488 nm OD2 filter]

- Threshold FL1 | B-525-40-H = 550;
- FL1 | B-525-40 Gain = 500

Cytek Bioscience, Aurora [405 nm OD0 filter, 488 nm OD2 filter]

- Threshold B2 | B-524.5-17-H = 500;
- B2 | B-524.5-17 Gain = 1750

9 Acquire the 'DPBS' tube while viewing the FITC histogram plot from go to step #6. Adjust the detector gain or trigger threshold until the instrument noise is being **acquired at ~1000 events/sec.**

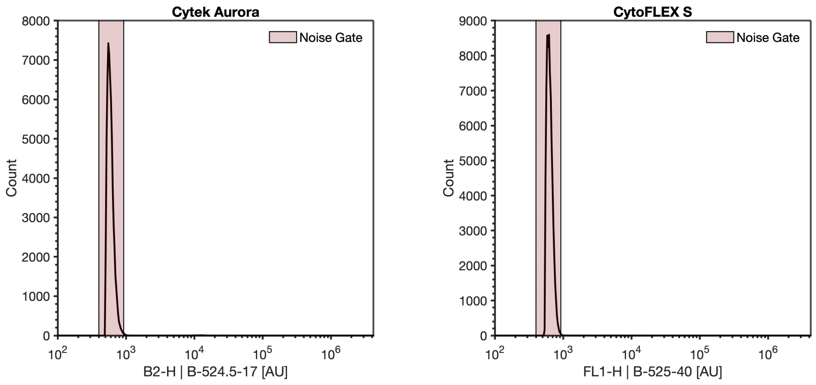

Acquisition of DPBS on the Cytek Aurora and CytoFLEX S, with the Aurora triggering on instrument noise on the B2-H channel at gain 1750 and threshold 500 and the CytoFLEX triggering on instrument noise on the FL1-H channel at gain 500 and threshold 550.

Note

The instrument noise floor is distinct from detected background events in sheath as it has a sharp increase. In a system with debris there may be a tail that elongates out of this this sharp peak.

9.1 Recording this noise is not necessary as this step is identifying optimal settings.

10 Acquire the "Beads" tube from go to step #2 . Using the plot from go to step #6 , ensure the FluoSpheres are visible on the FITC and violet SSC parameter. Use the FITC trigger settings identified in go to step #9

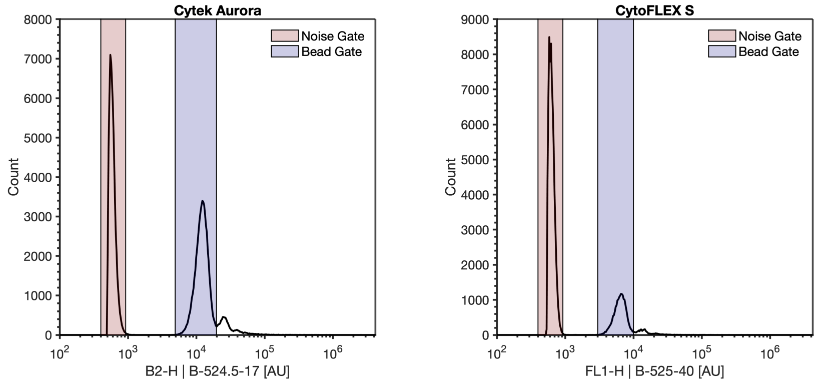

Acquisition of 110 nm FluoSpheres in DPBS on the Cytek Aurora and CytoFLEX S, with the Aurora triggering on instrument noise on the B2-H channel at gain 1750 and threshold 500 and the CytoFLEX triggering on instrument noise on the FL1-H channel at gain 500 and threshold 550.

Note

The total event rate when acquiring the FluoSpheres should not exceed 6000 events/sec. If the event rate is higher than 6000 events/sec, further dilution of the FluoSpheres is necessary prior to acquisition.

11 Draw a gate around the FluoSpheres on the FITC parameter. Label this gate 'Bead Gate'

12 Adjust the stopping criteria of the instrument to record until at least 2,000 events are acquired on 'Bead Gate' drawn in go to step #11 .

Performing Voltration

13 Voltration can now be performed by recording the 'Beads' tube at multiple light scatter detector gains, leaving the trigger threshold and fluorescent gains consistent. It is recommended that a recording of at least 10 light scatter detector settings is taken. Including more increments within a voltration will result in being more confident of the subsequent optimal detector settings.

Acquisition Template.xlsx

Note

For flow cytometers with avalanche photodiodes its is recommended that the detector settings have more incrementation at lower gains than higher gains while for instruments with photomultiplier tubes they should be spaced evenly. See template for example of settings for each tube analyses

Example Gain Voltration for CytoFLEX & Aurora

1. 50
2. 100
3. 200
4. 300
5. 400
6. 500
7. 750
8. 1000
9. 1250
10. 1500
11. 2000
12. 2500
13. 3000

13.1 To ensure accurate data analysis, the FluoSpheres must separate from the noise population on the light scatter parameter on at least two of the selected gains. If they do not, repeat this protocol using a 200 nm FluoSphere population.

B. Methods S2: Flow cytometer fluorescence detector setting incrementation, related to Figure 1.

Resource 2: Fluorescence Detector Setting Incrementation for FCMPASS

Forked from Flow Cytometer Fluorescence Voltration for FCMPASS

In 1 collection

Joshua A Welsh[1], Sean M Cook[1], Jennifer Jones[1]

[1]Translataional Nanobiology Section, Laboratory of Pathology, Center for Cancer Research, National Cancer Institute, National Institutes of Health

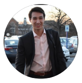

Sean M Cook

Protocol Info: Joshua A Welsh, Sean M Cook, Jennifer Jones . Resource 2: Fluorescence Detector Setting Incrementation for FCMPASS. **protocols.io** https://protocols.io/view/resource-2-fluorescence-detector-setting-increment-cnjjvckn

Created: Jan 30, 2023

Last Modified: Mar 15, 2023

PROTOCOL integer ID: 76107

DISCLAIMER

This protocol summarizes key steps for a specific type of method, which is one of a collection of methods and assays used for EV analysis in the NCI Translational Nanobiology Section at the time of submission of this protocol. Appropriate use of this protocol requires careful, cohesive integration with other methods for EV production, isolation, and characterization.

ABSTRACT

Protocol to perform flow cytometer voltration to identify optimal detector settings for small particle analysis. Data acquired from this protocol are compatible with semi-automated analysis tools built into FCMPASS software.

MATERIALS

- QbSure **Cytek Catalog #B7-10005** , referred to as 'QbSure'
- DPBS **Thermo Fisher Scientific Catalog #14190144** , referred to as 'DPBS'
- 5 mL Round-bottom tube **Corning Catalog #352052** , referred to as 'FACS tube'
- FITC-5 MESF Beads **Bangs Laboratories Catalog #555B** , referred to as 'FITC MESF beads'

Sample preparation

10s

1 Vortex QbSure bottle on a high setting for 00:00:05 . 5s

Note

This protocol in principle is compatible with all multi-peak rainbow beads. Validation has only been tested on QbSure and Spheretech 8-peak beads. QbSure beads have been highlighted due to having consistently cleaner negative populations in our testing.

2 Pipette 500 µL of DPBS to two FACS tubes. Label one tube 'DPBS" and the second tube 'Beads'.

Note

An observation from our protocol development is that it is important not to use a low protein binding tube for this step, as it can result in excess unbound fluor from the beads creating background noise increases leading to excessive event rate.

3 Add 3 drops of QbSure beads to the 'Beads' tube and vortex for 00:00:05 . 5s

Cytometer Setup

4 Ensure cytometer is clean and that -Height and -Area statistics are set to be collected on all parameters and that all parameters are on.

5 On the Cytek Aurora, set window extension to 0. On the CytoFLEX platform turn on 'High Acquisition Mode'.

Note

To set the window extension to 0 on the Cytek Aurora, navigate to the 'Lasers' tab under the sample acquisition settings and set the window extension to 0.

To change the acquisition mode on the CytoFLEX S, click on the 'Advanced' menu on the top-of-the-screen ribbon. Next click on 'Event Rate Settings' menu, change the acquisition mode to 'High', and press 'Ok'.

6 Create a pseudocolor plot with FSC-H on the X-Axis and (488 nm) B-SSC-H on the Y-Axis and

make sure both parameters are being plotted on a linear-scale.

7 Create a histogram plot with (405 nm) V-SSC-H on the X-Axis and make sure it is plotted on a log-scale.

8 Set the cytometer triggering threshold to (405 nm) V-SSC-H. **All samples should be acquired with the lowest flow rate, typically ~10-15 µL min^{-1}.**

Note

Cytometer Voltage/Gain and threshold settings are subjective due to their dependency on alignment, and the scatter filters in place, amongst other variables. The following are guide values to start with and may need adjustment for optimal acquisition.

Beckman Coulter, CytoFLEX [405 nm OD0 filter, 488 nm OD2 filter]
- Threshold V-SSC-H = 1000;
- V-SSC Gain = 200
- FSC Gain = 100
- B-SSC Gain = 100

Cytek Bioscience, Aurora [405 nm OD0 filter, 488 nm OD2 filter]
- Threshold V-SSC-H = 1000;
- V-SSC Gain = 2500
- FSC Gain = 150
- B-SSC Gain = 40

9 Acquire the DPBS tube while viewing the histogram plot from go to step #7. Adjust the detector gain or trigger threshold until the instrument noise is being **acquired at ~1000 events/sec.** The instrument noise floor is distinct from detected background events in sheath as it has a sharp increase. In a system with debris there may be a tail that elongates out of this this sharp peak.

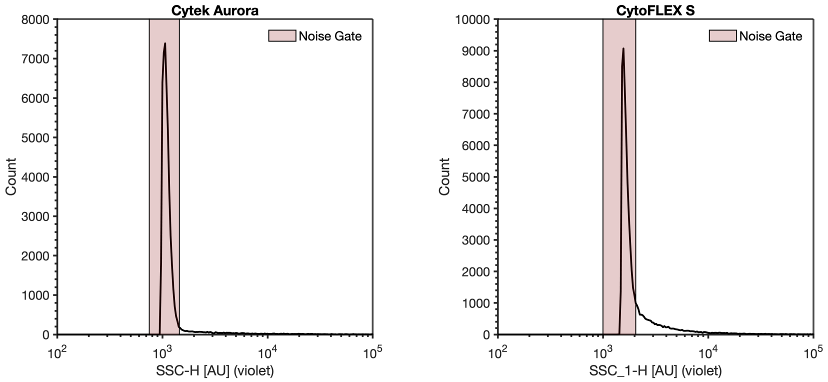

Example of threshold on the instrument noise floor on the Cytek Aurora and CytoFLEX S. Cytek Aurora triggered on SSC-H channel at gain 2500 and threshold 975. CytoFLEX S triggered on SSC_1-H channel at gain 250 and threshold 1200.

9.1 Recording this noise is not necessary as this step is identifying optimal settings.

10 Acquire the 'Beads' tube from go to step #3 . Using the plot from go to step #6 adjust the FSC and B-SSC gain until the single bead population is clearly visible and can be easily gated from the doublet population to the top right of it. Use the Violet SSC trigger settings identified in go to step #9

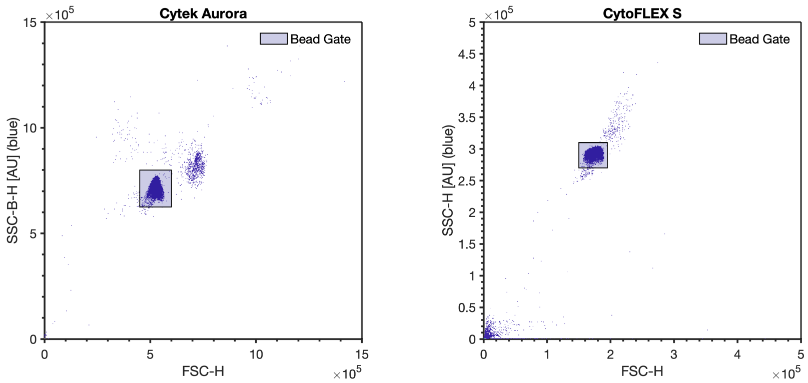

Example of clearly resolved singlet QbSure bead population on the Cytek Aurora and CytoFLEX S. Cytek Aurora FSC and SSC-B gains set to 150 and 40 respectively. CytoFLEX S FSC and SSC gains both set to 100.

11 Creating a gate around the single bead population named 'Bead Gate';

12 Adjust the stopping criteria of the instrument to record until 10,000 events are acquired on 'Bead Gate' drawn in go to step #11 .

Performing Voltration

13 Voltration can now be performed by recording the 'Beads' tube at multiple fluorescent detector gains, leaving the trigger threshold and light scatter gains consistent. It is recommended that a recording of at least 10 fluorescent detector settings is taken. Including more increments within a voltration will result in being more confident of the subsequent optimal detector settings.

Acquisition Template.xlsx

Note

For flow cytometers with avalanche photodiodes its is recommended that the fluorescent detector settings have more incrementation at lower gains than higher gains while for instruments with photomultiplier tubes they should be spaced evenly. See template for example of settings for each tube analyses

Example Gain Voltration for CytoFLEX & Aurora

1. 100
2. 200
3. 300
4. 400
5. 500
6. 750
7. 1000
8. 1250
9. 1500
10. 2000
11. 2500
12. 3000

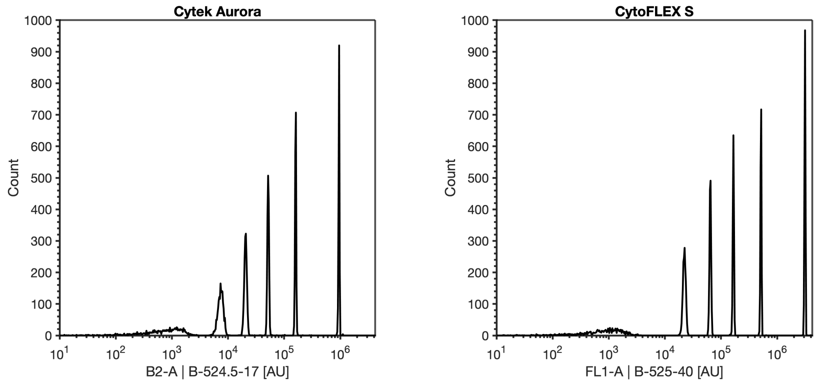

Example of QbSure beads acquired on Cytek Aurora and CytoFLEX S on the FITC parameter. Data has been gated to remove the noise population.

13.1 To ensure accurate data analysis, the brightest bead must be visible on at least two of the selected gains.

14 Fluorescent channels can be cross calibrated to determine lower limit of detection for the channel in calibrated units in the FCM_{PASS} software. A cross calibration between the desired MESF bead and QbSure beads should be acquired at a fluorescent detector gain where all MESF bead populations are **on-scale and fully resolved** from the noise.

Note

Cross calibration on Cytek Aurora and CytoFLEX were run with FITC MESF beads on the B2 | B-524.5-17 and FL1 | B-525-40 channels respectively. The QbSure beads were acquired at the same settings as the FITC MESF beads.

C. Methods S3: Flow cytometer light scatter calibration, related to Figure 1.

Resource 3: SSC Collection Optics and Calibration

Forked from FCMPASS - Acquisition and gating of light scatter reference materials

In 1 collection

Joshua A Welsh[1], Jennifer Jones[1]

[1]Translational Nanobiology Section, Laboratory of Pathology, Center for Cancer Research, National Cancer Institute, National Institutes of Health

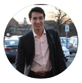

Sean M Cook

Protocol Info: Joshua A Welsh, Jennifer Jones . Resource 3: SSC Collection Optics and Calibration. **protocols.io** https://protocols.io/view/resource-3-ssc-collection-optics-and-calibration-cnmcvc2w

MANUSCRIPT CITATION:
Welsh J A, Jones J C,Small Particle Fluorescence and Light Scatter Calibration Using FCMPASSSoftware,Current Protocols in Cytometry, 94, e79. doi: 10.1002/cpcy.79

Created: Jan 31, 2023

Last Modified: Mar 25, 2023

PROTOCOL integer ID: 76164

Keywords: flow cytometry, calibration, extracellular vesicles

DISCLAIMER

This protocol summarizes key steps for a specific type of method, which is one of a collection of methods and assays used for EV analysis in the NCI Translational Nanobiology Section at the time of submission of this protocol. Appropriate use of this protocol requires careful, cohesive integration with other methods for EV production, isolation, and characterization.

Terms & Conditions of use for FCMPASS software.

Definitions: The term "SOFTWARE" throughout this agreement means the machine readable, binary, object code form, and the related documentation for FCMPASS, a software package that is designed to allow flow cytometer calibration for small particles. The term "RECIPIENT" means the party that downloads the software. The term "PROVIDER" means the National Cancer Institute (NCI), a participating institute of the National Institutes of Health (NIH), and an agency of the United States Government.By downloading or otherwise receiving the SOFTWARE, RECIPIENT may use the SOFTWARE subject to RECIPIENT's agreement to the following terms:

1. THE SOFTWARE SHALL NOT BE USED IN THE TREATMENT OR DIAGNOSIS OF HUMAN SUBJECTS. RECIPIENT is responsible for compliance with all laws and regulations applicable to the use of the SOFTWARE.
2. RECIPIENT shall not distribute the SOFTWARE, in whole or in part without express advance written approval of PROVIDER.
3. The SOFTWARE may be used for research, academic, and educational purposes only. The SOFTWARE may not be used for commercial purposes. RECIPIENT will not license or sell or use the SOFTWARE for commercial purposes or applications.
4. The SOFTWARE that is distributed pursuant to this Agreement has been created by United States Government employees. In accordance with Title 17 of the United States Code, section 105, the SOFTWARE isnot subject to copyright protection in the United States. Other than copyright, all rights,

title and interest in the SOFTWARE shall remain with the PROVIDER.

5. RECIPIENT shall not modify, extend, decompile, make derivatives of, merge, publish, reverse engineer or distribute the SOFTWAREwithout written permission from PROVIDER.
6. RECIPIENT may publish or otherwise publicly disclose the results of using the SOFTWARE. RECIPIENT agrees to acknowledge PROVIDER's contribution of the SOFTWARE in all written publications containing any data or information regarding or resulting from use of the SOFTWARE.
7. THE SOFTWARE IS PROVIDED "AS IS" AND ANY EXPRESS OR IMPLIED WARRANTIES, INCLUDING, BUT NOT LIMITED TO, THE IMPLIED WARRANTIES OF MERCHANTABILITY, FITNESS FOR A PARTICULAR PURPOSE AND NONINFRINGEMENT ARE DISCLAIMED. IN NO EVENT SHALL THE PROVIDER OR THE INDIVIDUAL DEVELOPERS BE LIABLE FOR ANY DIRECT, INDIRECT, INCIDENTAL, SPECIAL, EXEMPLARY, OR CONSEQUENTIAL DAMAGES (INCLUDING, BUT NOT LIMITED TO, PROCUREMENT OF SUBSTITUTE GOODS OR SERVICES; LOSS OF USE, DATA, OR PROFITS; OR BUSINESS INTERRUPTION) HOWEVER CAUSED AND ON ANY THEORY OF LIABILITY, WHETHER IN CONTRACT, STRICT LIABILITY, OR TORT (INCLUDING NEGLIGENCE OR OTHERWISE) ARISING IN ANY WAY OUT OF THE USE OF THIS SOFTWARE, EVEN IF ADVISED OF THE POSSIBILITY OF SUCH DAMAGE. PROVIDER makes no representations that the use of SOFTWARE will not infringe any patent or proprietary rights of third parties.
8. RECIPIENT may, on an as-needed basis, send to PROVIDER reports regarding the application of the SOFTWARE and the effectiveness and problems encountered in using the SOFTWARE, without disclosing RECIPIENT's confidential information. Information from general reports may be used by the PROVIDER to enhance the capabilities of the SOFTWARE. Reports can be forwarded to the PROVIDER at one of the following addresses: joshua.welsh@nih.gov or jennifer.jones2@nih.gov
9. No indemnification for any loss, claim, damage, or liability is intended or provided by either Party under this Agreement. Each Party shall be liable for any loss, claim, damage, or liability that said Party incurs as a result of said Party's activities under this Agreement, except that Provider, as an agency of the United States, assumes liability only to the extent as provided under the Federal Tort Claims Act (28 U.S.C. Chapter 171 Sections 2671-2680).
10. RECIPIENT agrees not to claim, infer, or imply endorsement by the United States Government, or any of its organizational units, contractors or employees. RECIPIENT agrees not to use any trademarks, service marks, trade names, logos or product names of NCI or NIH to endorse or promote products derived from the SOFTWARE without specific, prior and written permission.
11. Title in the SOFTWARE shall remain with the PROVIDER. It is understood that nothing herein will be deemed to constitute, by implication or otherwise, the grant to either party by the other of any license or other rights under any patent, patent application or other intellectual property

right or interest. PROVIDER reserves the right to distribute the SOFTWARE to others and to use it for PROVIDER's own purposes. The United States Government explicitly retains all rights to use the SOFTWARE for any purpose, to have it used on the Government's behalf or to allow others to use it.

ABSTRACT

This protocol outlines the steps required to collect data from light scatter reference materials for use with the FCMPASS software. This is one of a number of protocols in the pipeline for performing small particle calibration using the fcmpass software package.

MATERIALS

MATERIALS

- Flow Cytometer **Contributed by users**
- Vortex **Contributed by users**
- Low Protein Binding Collection Tubes (1.5 mL) **Thermo Fisher Scientific Catalog #90410**
- DPBS **Invitrogen - Thermo Fisher Catalog #14190**
- NIST-Traceable Polystyrene Beads (3000 Series) **Thermo Fisher Scientific**
- Falcon® 5 mL Round Bottom Polystyrene Test Tube with Snap Cap Sterile Individually Wrapped **Corning Catalog #352003**

1 Calculate the stock traceable size calibration reference bead particle concentration using percent solids value and particle density provided by the manufacturer and the following formula, whereis the concentration (particles mL^{-1}),, is the percent solids,is the particle density (g mL^{-1}), andis the average diameter (µm).

$$N_P=\frac{(W_V\%,\times 6\times10^{12})}{(\pi\rho_\rho D^3)}$$

Note

For example, 100 nm polystyrene beads at 1% with 1.05 g mL^{-1}would be calculated using:

$$1.82\times10^{13}=\frac{(0.01\times 6\times10^{12})}{(\pi\times1.05\times 0.1^3)}$$

A online calculator can also be found at this link.

2 Thoroughly vortex the traceable size calibration reference bead stock bottles to homogenize the mixtures before dispensing 1 drop (~50 µL) into separate 500 µL low-protein binding Eppendorf.

3 Using the working stock from step 2, make up 500 µL solution at $1x10^7$ particles mL^{-1}.

Note

It is recommended that serial dilutions are used and volumes of no less than 10 µL to avoid pipetting errors. The optimal particle concentration at which to run the reference materials will vary depending on several factors, including the flow rate, beam height, and electronic sampling rate. If running for the first time, it is recommended that serial dilutions are performed to determine the optimal concentration for preparation of the beads.

4 On the flow cytometer, set the triggering threshold to the most sensitive light scatter detector and ensure the parameter is using log-scaling (not linear or biexponential).

5 Running DPBS, lower the triggering threshold until the noise floor of the instrument becomes visible. This is most clearly when using a histogram.

5.1 Plotting the trigger-channel height parameter against time and monitoring while running DPBS is a good indication for determining whether an instrument is clean. If the spread of noise (and event rate) decreases over time, it is indicative that the instrument was dirty and is becoming cleaner.

Note

There are a number of cleaning solutions. Some shared resource lab managers have a preference. These include bleach, contrad 70, micro 90, surfanol

5.2 The extent to which the opto-electronic noise of an instrument can be sampled will vary between instruments. Legacy flow cytometers will tolerate a couple of 1000-2000 events/second whilst allowing room to sample desired events, while high-speed jet-in-air sorters are capable of sample 10,000+ events per second.

5.3 Triggering using a light scatter parameter on the opto-electronic noise of the instrument has benefits in determining and tracking the lower limit of detection, as well as being informative for buffer + reagent controls where background fluorescence will show clear shifts due to many events being triggered from sampling the noise. The use of this method comes at the cost of having high event rates and therefore larger files. Before utilizing this method the instrument should be validated to determine: 1) its ability to detect and accurately process

particles, 2) the event rate at which single small particles are detected, and 3) the degree to which the opto-electronic noise can be sampled without creating artefacts or reducing the ability to detect genuine events.

5.4 On some instruments that utilize peristaltic pumps there can appear to be an increase and decrease of the baseline corresponding to the turnover of the pump. This is a result of the threshold being set close to (but above) the electronic noise, resulting in the increase and decrease in trigger events in light scatter. This can be overcome by lowering the threshold so that the noise is being sampled regardless of the peristaltic pump turnover or increasing the threshold and therefore decreasing the instrument's limit of sensitivity.

6 Analyze each bead sample at the same acquisition settings until >5000 bead events are recorded.

6.1 It is preferable to analyze and store bead populations individually. This will minimize population overlap, aggregates, background noise, and artifacts.

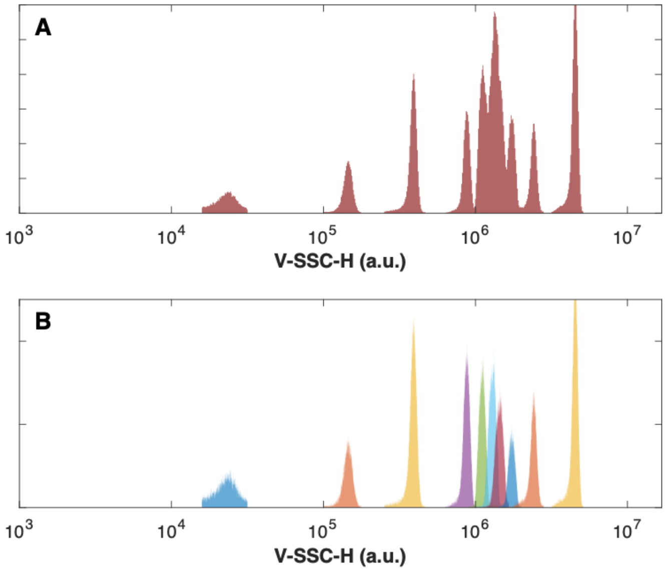

Panel A demonstrates the cumulative distribution of the gated populations when mixed together. While some populations are clearly distinguished some are not. The areas where bunching of populations occurs is dependent upon the cytometer and is useful in determining the collection angle. Panel B illustrates overlaid and colored gated bead population from Oanel A.

7 Gate each bead population using the parameter Height vs. Area in a dot-plot to remove doublets/aggregates and then use a histogram on the light scatter parameter (Height) to obtain statistics for each population. The light scatter parameter should use log scaling.

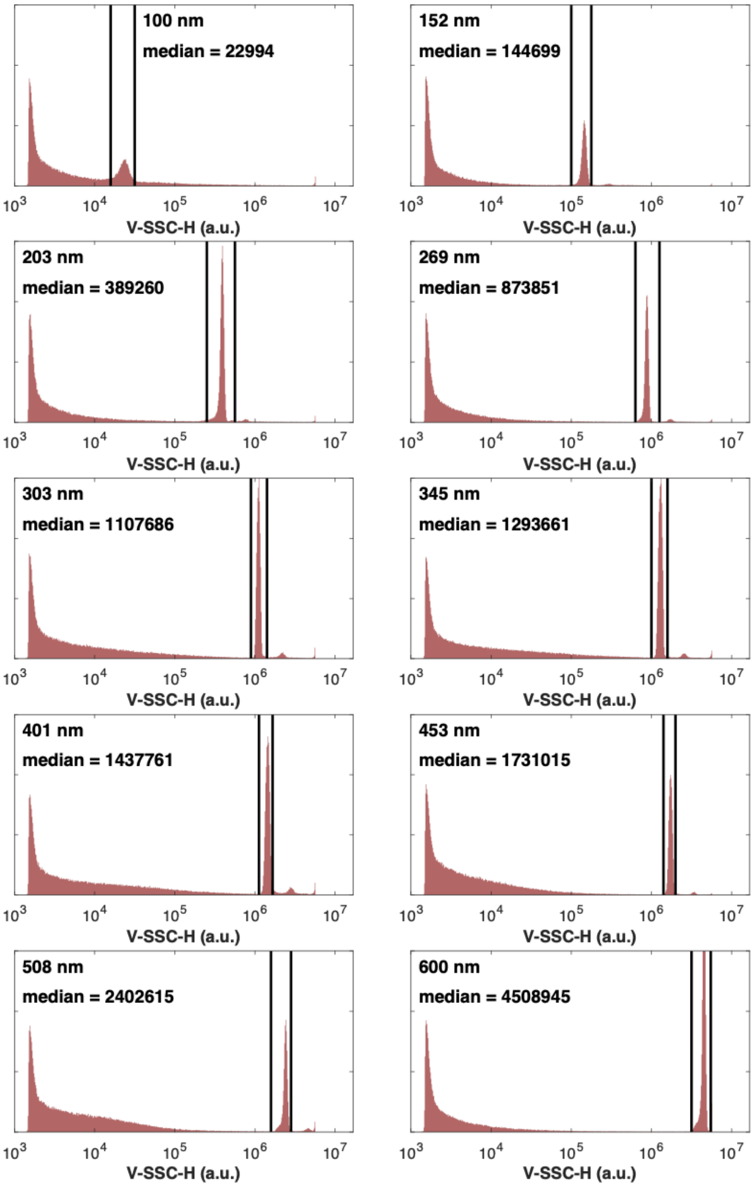

Gating light scatter reference beads. Each panel shows the gating of polystyrene NIST-traceable reference beads ranging in mean diameter from 100 to 600 nm. The median light scatter statistic of the gated population is given in each panel

8 Obtain the median statistic for each of the bead populations.

8.1 By default, flow cytometers trigger the acquisition of an event using the pulse height parameter. In cases where a trigger threshold is being defined (e.g. SSC), it is recommended that the pulse-height is used so that the limit of detection can be defined in calibrated units. There is no consensus within the small particle community over the use of pulse height vs. area. We recommend that, in general, if the parameter being calibrated was not used as a trigger channel the pulse area statistic should be used due to the tendency for low signal intensities to be linear and therefore a more reliable method for extrapolation.

D. Methods S4: Flow cytometer rEV serial dilution, related to Figure 1.

Resource 4: rEV Serial Dilution

Forked from rEV Acquisition

In 1 collection

Sean M Cook[1], Vera A. Tang[2], Joanne Lannigan[3], Jennifer Jones[1], Joshua A Welsh[4]

[1]Laboratory of Pathology, Translational Nanobiology Section, Centre for Cancer Research, National Institute of Health, National Institutes of Health; [2]University of Ottawa; [3]Flow Cytometry Support Services; [4]Translataional Nanobiology Section, Laboratory of Pathology, Center for Cancer Research, National Cancer Institute, National Institutes of Health

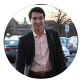

Sean M Cook

Protocol Info: Sean M Cook, Vera A. Tang, Joanne Lannigan, Jennifer Jones, Joshua A Welsh . Resource 4: rEV Serial Dilution. **protocols.io** https://protocols.io/view/resource-4-rev-serial-dilution-cnjkvckw

Created: Jan 30, 2023

Last Modified: Mar 25, 2023

PROTOCOL integer ID: 76108

DISCLAIMER

This protocol summarizes key steps for a specific type of method, which is one of a collection of methods and assays used for EV analysis in the NCI Translational Nanobiology Section at the time of submission of this protocol. Appropriate use of this protocol requires careful, cohesive integration with other methods for EV production, isolation, and characterization.

MATERIALS

Exosome standards fluorescent **Merck MilliporeSigma (Sigma-Aldrich) Catalog #SAE0193**, referred to as 'rEVs'

DPBS **Thermo Fisher Scientific Catalog #14190144**, referred to as 'DPBS'

Low Protein Binding Collection Tubes (1.5 mL) **Thermo Fisher Scientific Catalog #90410**, referred to as 'low-binding Eppendorf tubes'

QbSure **Cytek Catalog #B7-10005**, referred to as 'QbSure'

100 nm polystyrene NIST bead **Thermo Fisher Scientific Catalog #3100A**, referred to as '100 nm NIST bead'

rEV Reconstitution

1 Briefly centrifuge 100 x g, 4°C, 00:05:00 rEVs before opening. 5m

2 Add 100 µL of 4 °C deionized water.

3 Pipet up and down to mix.

Note

Do not vortex

4 Aliquot 20 µL into low-binding Eppendorf tubes.

Equipment

**Low Protein Binding Microcentrifuge Tubes**	NAME
Microcentrifuge Tubes	TYPE
Thermo Scientific	BRAND
90410	SKU
https://www.thermofisher.com/order/catalog/product/90410	LINK

5 Store at 2-4 °C for up to one day (up to 2 weeks at -80 °C).

rEV Dilutions

6 Label 10 low-binding Eppendorf tubes:

A	B
Label	Dilution
1	50
2	100

A	B
3	200
4	400
5	800
6	1600
7	3200
8	6400
9	12800
10	25600

7 Add 980 µL of DPBS (preferably 0.1 µm filtered) to the tube 1 from go to step #6 .

8 Add 500 µL of DPBS (preferably 0.1 µm filtered) to the remaining tubes.

9 Add 20 µL of the stock reconstituted rEVs to the tube labeled 1 (go to step #6), pipet up and down to mix.

Note

Do not vortex

10 Pipette 500 µL of the 1:50 dilution in tube 1 into the 1:100 dilution in tube 2, pipet up and down to mix.

Note

Do not vortex

11 Repeat the above step for each of the remaining dilution tubes until the serial dilution has been completed for all dilutions.

12 Keep on ice during acquisition.

Flow Cytometer Acquisition

13 Use the instrument settings that were established from the previous gain incrementation and scatter calibration resources. On the Aurora, set the window extension to 0. On the CytoFLEX, use the high acquisition mode. Collect for at least 1 minute at a low flow rate with a 30 second recording delay if using plate mode.

Note

To set the window extension to 0 on the Cytek Aurora, navigate to the 'Lasers' tab under the sample acquisition settings and set the window extension to 0.

To change the acquisition mode on the CytoFLEX S, click on the 'Advanced' menu on the top-of-the-screen ribbon. Next click on 'Event Rate Settings' menu, change the acquisition mode to 'High', and press 'Ok'.

13.1 Set your instrument to trigger on the most sensitive side scatter channel at the FCM_{PASS} optimal gain output as determined by the FluoSpheres scatter voltration. Set the threshold in the noise such that the event rate is ~1000 events/sec when running DPBS.

Protocol

NAME

Resource 1: Scatter Detector Setting Incrementation for FCMPASS

CREATED BY

Sean M Cook

PREVIEW

13.2 Set the FITC parameter to the FCM_{PASS} optimal gain output as determined by the QbSure fluorescent voltration.

Protocol

NAME

Resource 2: Fluorescence Detector Setting Incrementation for FCMPASS

CREATED BY

Sean M Cook

PREVIEW

14 Collect a DPBS sample in a FACS tube for one minute on low.

Note

This is to ensure the instrument is clean on the side scatter and fluorescent channels before acquiring the rEVs.

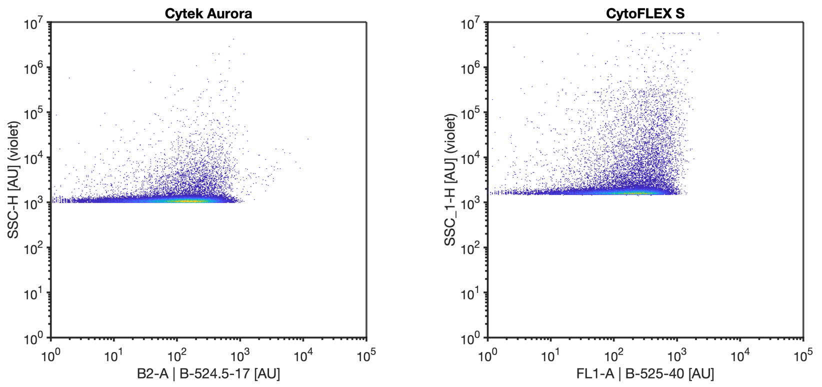

DPBS acquired on the Cytek Aurora and CytoFLEX S.

15 Collect all rEVs samples for one minute on low, starting with the highest dilution (least concentrated) and working down to lowest dilution (most concentrated). This will reduce the effect of carry over from high concentrations.

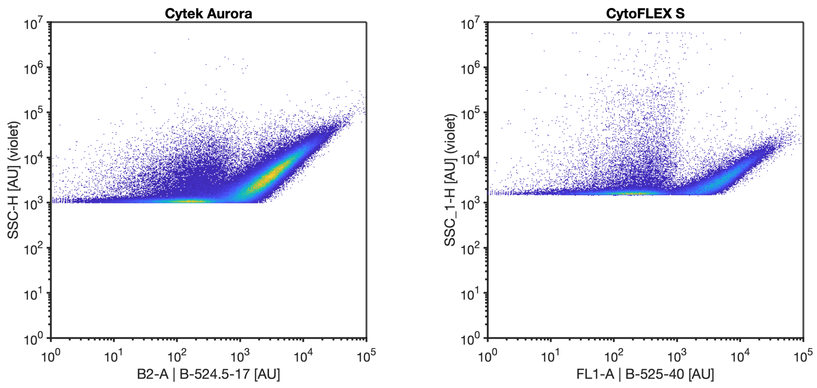

rEVs acquired on the Cytek Aurora and CytoFLEX S.

16 Run a DPBS sample in a FACS tube to make sure there are no carryover events; there should be no events in the B1 and B2 channel.

Note

If there is carry over clean instrument with bleach and flush with deionized water as appropriate.

17 Label as FACS tube as 'QbSure', and add 500 µL DPBS. Vortex the QbSure beads for 5 sec and add 3 drops QbSure beads into FACS tube. Run QbSure beads on low and collect 10,000 bead events.

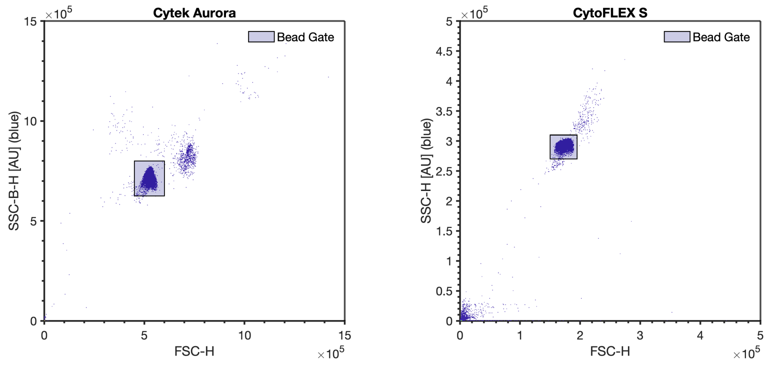

QbSure beads gated on blue SSC-H and FSC-H on the Cytek Aurora and CytoFLEX S. The violet SSC settings remain unchanged. In order to limit .fcs file size, a FSC scatter trigger may be used until 10000 QbSure bead events are acquired.

Note

You may need to adjust the B-SSC and FSC detector settings for the bead acquisition. Do not adjust fluorescence detector settings or high sensitivity SSC detector settings. Assuming the QbSure beads were cross calibrated in the previous protocols, this allows for the calibration of the rEV fluorescent data into calibrated units.

18 Run the 100 nm polystyrene NIST beads at the same settings as the rEVs (diluted in DPBS to a concentration of 5E6 p/mL) until at least 10000 bead events have been acquired.

Note

Acquiring the 100 nm polystyrene NIST bead allows for the calibration of the rEV side scatter data into calibrated units if the collection half-angle of the instrument has been determined in previous protocols.

19 If calibration of data into standard units is desired, the 100 nm polystyrene NIST bead and QbSure beads can be used to calibrate data by following the FCM_{PASS} experiment calibration protocols.

Protocol

NAME

FCMPASS Protocol Collection

CREATED BY

Joshua A Welsh

PREVIEW

E. Methods S5: Flow cytometer rEV light scatter detector setting incrementation, related to Figure 1.

Resource 5: rEV Scatter Detector Setting Incrementation

In 1 collection

Sean M Cook[1], Vera A. Tang[2], Joanne Lannigan[3], Jennifer Jones[1], Joshua A Welsh[1]

[1]Translataional Nanobiology Section, Laboratory of Pathology, Center for Cancer Research, National Cancer Institute, National Institutes of Health; [2]University of Ottawa; [3]Flow Cytometry Support Services

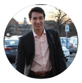

Sean M Cook

Protocol Info: Sean M Cook, Vera A. Tang, Joanne Lannigan, Jennifer Jones, Joshua A Welsh . Resource 5: rEV Scatter Detector Setting Incrementation. **protocols.io** https://protocols.io/view/resource-5-rev-scatter-detector-setting-incrementa-cnjpvcmn

Created: Jan 30, 2023

Last Modified: Mar 25, 2023

PROTOCOL integer ID: 76111

DISCLAIMER

This protocol summarizes key steps for a specific type of method, which is one of a collection of methods and assays used for EV analysis in the NCI Translational Nanobiology Section at the time of submission of this protocol. Appropriate use of this protocol requires careful, cohesive integration with other methods for EV production, isolation, and characterization.

MATERIALS

Exosome standards fluorescent **Merck MilliporeSigma (Sigma-Aldrich) Catalog #SAE0193**, referred to as 'rEVs'

DPBS **Thermo Fisher Scientific Catalog #14190144**, referred to as 'DPBS'

5 mL Round-bottom tube **Corning Catalog #352052**, referred to as 'FACS tube'

Protein LoBind Tubes (5 mL) **Thermo Fisher Scientific Catalog #0030122356**, referred to as 'low-binding Eppendorf tubes'

QbSure **Cytek Catalog #B7-10005**, referred to as 'QbSure'

100 nm polystyrene NIST bead **Thermo Fisher Scientific Catalog #3100A**, referred to as '100 nm NIST bead'

Sample Preprepation

5m

1 Briefly centrifuge rEVs at 100 x g, 4°C, 00:05:00 before opening 5m

2 Add 100 µL of 4 °C deionized water to rEVs vial. Pipette up and down to mix well.

Note

Do not vortex

3 Create 5 mL of a 1:2000 dilution of rEVs by pipetting 1 µL rEVs into 1999 µL DPBS in a low-binding Eppendorf tube. This gives a 5E6 p/mL solution.

4 Label a FACS tube as 'DPBS'. Pipette 500 µL DPBS into the tube.

5 Label as FACS tube as 'QbSure', and add 500 µL DPBS. Vortex the QbSure beads for 5 sec and add 3 drops QbSure beads into FACS tube.

Cytometer Setup

6 Ensure cytometer is clean and that -Height and -Area statistics are set to be collected on all parameters and that all parameters are on.

7 On the Cytek Aurora, set window extension to 0. On the CytoFLEX platform turn on 'High Acquisition Mode'.

Note

To set the window extension to 0 on the Cytek Aurora, navigate to the 'Lasers' tab under the sample acquisition settings and set the window extension to 0.

To change the acquisition mode on the CytoFLEX S, click on the 'Advanced' menu on the top-of-the-screen ribbon. Next click on 'Event Rate Settings' menu, change the acquisition mode to 'High', and press 'Ok'.

8 Create a histogram plot with the FITC parameter (CytoFLEX: FL1 | B-525-40, Aurora: B2 | B-524.5-17) on the X-Axis and make sure it is plotted on a log-scale.

9 Create a histogram plot with (405 nm) V-SSC-H on the X-Axis and make sure it is plotted on a log-scale.

10 Set the cytometer triggering threshold to the FITC parameter at the same settings used to acquire the bead scatter voltration . **All samples should be acquired with the lowest flow rate, typically ~10-15 µL min^{-1}.**

11 Acquire the 'DPBS' tube while viewing the FITC histogram plot from go to step #9 . Adjust the detector gain or trigger threshold until the instrument noise is being **acquired at ~1000 events/sec.** The instrument noise floor is distinct from detected background events in sheath as it has a sharp increase. In a system with debris there may be a tail that elongates out of this this sharp peak.

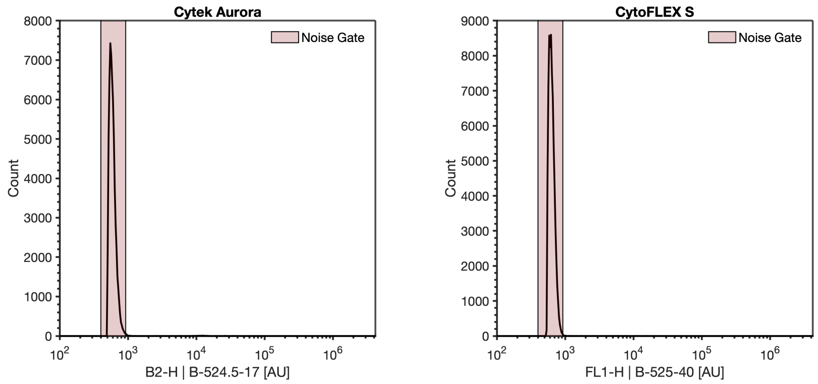

Acquisition of DPBS on the Cytek Aurora and CytoFLEX S, with the Aurora triggering on instrument noise on the B2-H channel at gain 1750 and threshold 500 and the CytoFLEX triggering on instrument noise on the FL1-H channel at gain 500 and threshold 550.

11.1 Recording this noise is not necessary as this step is for identifying optimal settings.

rEV Acquisition

12 Validation of FCM_{PASS} outputs can now be performed by acquiring the rEVs at the same voltration gains used when acquiring the FluoSpheres scatter voltration.

Note

For flow cytometers with avalanche photodiodes its is recommended that the detector settings have more incrementation at lower gains than higher gains while for instruments with photomultiplier tubes they should be spaced evenly. See template for example of settings for each tube analyses

Example Gain Voltration for CytoFLEX & Aurora

1. 50
2. 100
3. 200
4. 300
5. 400
6. 500
7. 750
8. 1000
9. 1250
10. 1500
11. 2000
12. 2500
13. 3000

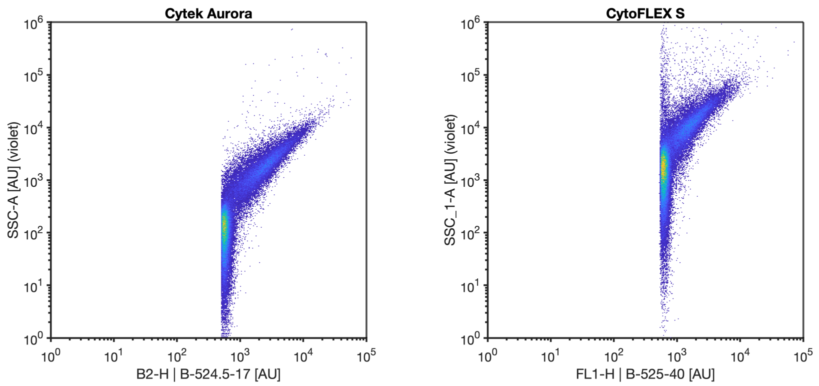

Example of rEVs acquired on the Cytek Aurora and CytoFLEX S. Cytek Aurora triggered on B2-H | B-524.5-17 at gain 1750 and threshold 500. CytoFLEX S triggered on violet FL1-H | B-525-40 at gain 500 and threshold 550.

Note

The total event rate when acquiring the rEVs should not exceed 6000 events/sec. If the event rate is higher than 6000 events/sec, further dilution of the rEVs is necessary prior to acquisition.

13 Pipette 200 µL from the 5E6 rEV solution from go to step #3 into a FACS tube. Acquire the FACS tube for 60 s on the lowest flow rate at one of the scatter gains. Repeat this for each gain.

Note

In order to prevent the crashing out of rEVs over the time course of the experiment, only pipette from 5E6 rEV solution into a FACS tube immediately prior to acquisition. Reverse pipette to mix in the FACS tube, do not vortex.

14 Run the 100 nm polystyrene NIST beads at the same settings as the rEVs (diluted in DPBS to a concentration of 5E6 p/mL) until at least 10000 bead events have been acquired for each gain.

Note

Acquiring the 100 nm polystyrene NIST bead allows for the calibration of the rEV side scatter data into calibrated units if the collection half-angle of the instrument has been determined in previous protocols.

15 Run QbSure beads on low at the same fluorescent settings as the rEVs and collect 10,000 bead events. QbSure beads only need to be acquired once at the fluorescent settings used in the experiment.

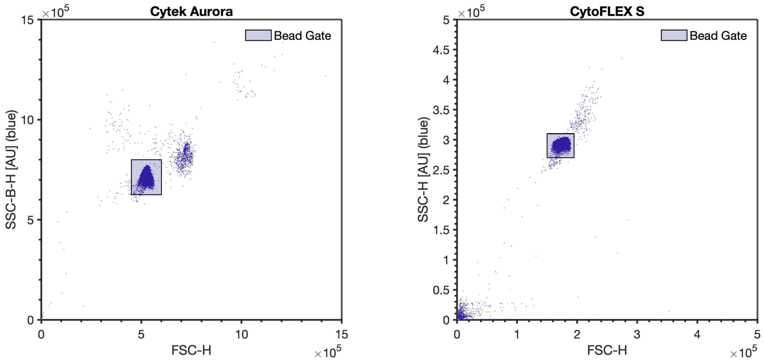

QbSure beads gated on blue SSC-H and FSC-H on the Cytek Aurora and CytoFLEX S. The violet SSC settings remain unchanged. In order to limit .fcs file size, a FSC scatter trigger may be used until 10000 QbSure bead events are acquired.

Note

You may need to adjust the B-SSC and FSC detector settings for the bead acquisition. Do not adjust fluorescence detector settings or high sensitivity SSC detector settings. Assuming the QbSure beads were cross calibrated in the previous protocols, this allows for the calibration of the rEV fluorescent data into calibrated units.

16 If calibration of data into standard units is desired, the 100 nm polystyrene NIST bead and QbSure beads can be used to calibrate data by following the FCM_{PASS} experiment calibration protocols.

Protocol

NAME

FCMPASS Protocol Collection

CREATED BY

Joshua A Welsh

PREVIEW

Note

Not all scatter gains will resolve the 100 nm NIST bead, but the data can still be calibrated using the 110 nm FluoSpheres that were collected in the FluoSpheres scatter voltration.

F. Methods S6: Flow cytometer rEV fluorescence detector setting incrementation, related to Figure 1.

Resource 6: rEV Fluorescent Detector Setting Incrementation

In 1 collection

Sean M Cook[1], Vera A. Tang[2], Joanne Lannigan[3], Jennifer Jones[1], Joshua A Welsh[1]

[1]Translataional Nanobiology Section, Laboratory of Pathology, Center for Cancer Research, National Cancer Institute, National Institutes of Health; [2]University of Ottawa; [3]Flow Cytometry Support Services

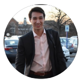

Sean M Cook

Protocol Info: Sean M Cook, Vera A. Tang, Joanne Lannigan, Jennifer Jones, Joshua A Welsh . Resource 6: rEV Fluorescent Detector Setting Incrementation. **protocols.io** https://protocols.io/view/resource-6-rev-fluorescent-detector-setting-increm-cnjivcke

Created: Jan 30, 2023

Last Modified: Mar 25, 2023

PROTOCOL integer ID: 76106

DISCLAIMER

This protocol summarizes key steps for a specific type of method, which is one of a collection of methods and assays used for EV analysis in the NCI Translational Nanobiology Section at the time of submission of this protocol. Appropriate use of this protocol requires careful, cohesive integration with other methods for EV production, isolation, and characterization.

MATERIALS

Exosome standards fluorescent **Merck MilliporeSigma (Sigma-Aldrich) Catalog #SAE0193**
, referred to as 'rEVs'
DPBS **Thermo Fisher Scientific Catalog #14190144** , referred to as 'DPBS'
5 mL Round-bottom tube **Corning Catalog #352052** , referred to as 'FACS tube'
Protein LoBind Tubes (5 mL) **Thermo Fisher Scientific Catalog #0030122356**
, referred to as 'low-binding Eppendorf tubes'
QbSure **Cytek Catalog #B7-10005** , referred to as 'QbSure'
100 nm polystyrene NIST bead **Thermo Fisher Scientific Catalog #3100A**
, referred to as '100 nm NIST bead'

Sample Preprepation

5m

1 Briefly centrifuge rEVs at 100 x g, 4°C, 00:05:00 before opening

5m

2 Add 100 µL of 4 °C deionized water to rEV vial. Pipette up and down to mix well.

> **Note**
>
> Do not vortex

3 Create 5 mL of a 1:2000 dilution of rEVs by pipetting 1 µL rEVs into 1999 µL DPBS in a low-binding Eppendorf tube. This gives a 5E6 p/mL solution.

4 Label a FACS tube as 'DPBS'. Pipette 500 µL DPBS into the tube.

5 Label as FACS tube as 'QbSure', and add 500 µL DPBS. Vortex the QbSure beads for 5 sec and add 3 drops QbSure beads into FACS tube.

Cytometer Setup

6 Ensure cytometer is clean and that -Height and -Area statistics are set to be collected on all parameters and that all parameters are on.

7 On the Cytek Aurora, set window extension to 0. On the CytoFLEX platform turn on 'High Acquisition Mode'.

> **Note**
>
> To set the window extension to 0 on the Cytek Aurora, navigate to the 'Lasers' tab under the sample acquisition settings and set the window extension to 0.
>
> To change the acquisition mode on the CytoFLEX S, click on the 'Advanced' menu on the top-of-the-screen ribbon. Next click on 'Event Rate Settings' menu, change the acquisition mode to 'High', and press 'Ok'.

8 Create a histogram plot with the FITC parameter (CytoFLEX: FL1 | B-525-40, Aurora: B2 | B-524.5-17) on the X-Axis and make sure it is plotted on a log-scale.

9 Create a histogram plot with (405 nm) V-SSC-H on the X-Axis and make sure it is plotted on a log-scale.

10 Set the cytometer triggering threshold to the violet SSC parameter at the same settings used to acquire the bead fluorescent voltration. **All samples should be acquired with the lowest flow rate, typically ~10-15 µL min^{-1}.**

11 Acquire the 'DPBS' tube while viewing the SSC histogram plot from go to step #9. Adjust the detector gain or trigger threshold until the instrument noise is being **acquired at ~1000 events/sec.** The instrument noise floor is distinct from detected background events in sheath as it has a sharp increase. In a system with debris there may be a tail that elongates out of this this sharp peak.

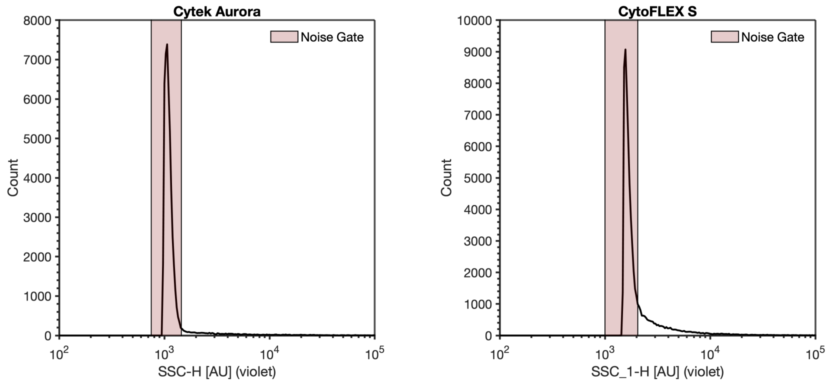

Example of threshold on the instrument noise floor on the Cytek Aurora and CytoFLEX S. Cytek Aurora triggered on SSC-H channel at gain 2500 and threshold 975. CytoFLEX S triggered on SSC_1-H channel at gain 250 and threshold 1200.

11.1 Recording this noise is not necessary as this step is for identifying optimal settings.

rEV Acquisition

12 Validation of FCM_{PASS} outputs can now be performed by acquiring the rEVs at the same voltration gains used when acquiring the QbSure fluorescence voltration.

Note

For flow cytometers with avalanche photodiodes its is recommended that the detector settings have more incrementation at lower gains than higher gains while for instruments with photomultiplier tubes they should be spaced evenly. See template for example of settings for each tube analyses

Example Gain Voltration for CytoFLEX & Aurora

1. 50
2. 100
3. 200
4. 300
5. 400
6. 500
7. 750
8. 1000
9. 1250
10. 1500
11. 2000
12. 2500
13. 3000

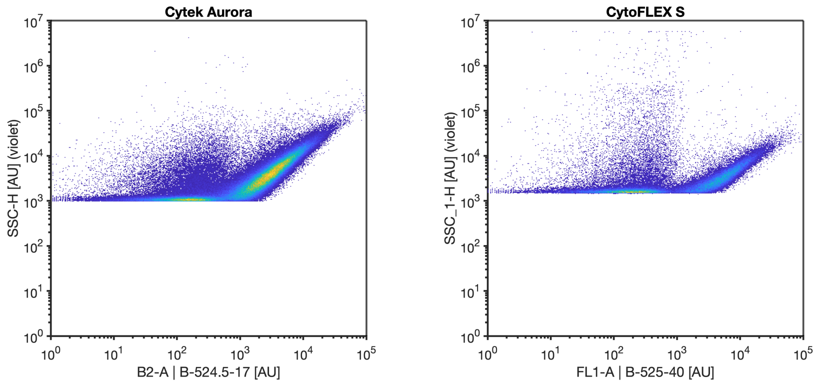

Example of rEVs acquired on the Cytek Aurora and CytoFLEX S. Cytek Aurora triggered on violet SSC-H at gain 2500 and threshold 975. CytoFLEX S triggered on violet SSC_1-H at gain 250 and threshold 1200.

4

Note

The total event rate when acquiring the rEVs should not exceed 6000 events/sec. If the event rate is higher than 6000 events/sec, further dilution of the rEVs is necessary prior to acquisition.

13 Pipette 200 µL from the 5E6 rEV solution from go to step #3 into a FACS tube. Acquire the FACS tube for 60 s on the lowest flow rate at one of the fluorescence gains. Repeat this for each gain.

Note

In order to prevent the crashing out of rEVs over the time course of the experiment, only pipette from 5E6 rEV solution into a FACS tube immediately prior to acquisition. Reverse pipette to mix in the FACS tube, do not vortex.

14 Run the 100 nm polystyrene NIST beads at the same scatter settings as the rEVs (diluted in DPBS to a concentration of 5E6 p/mL) until at least 10000 bead events have been acquired. The 100 nm bead only needs to be acquired once at the scatter settings used in the experiment.

Note

Acquiring the 100 nm polystyrene NIST bead allows for the calibration of the rEV side scatter data into calibrated units if the collection half-angle of the instrument has been determined in previous protocols.

15 Run QbSure beads on low at the same fluorescent settings and collect 10,000 bead events for each gain.

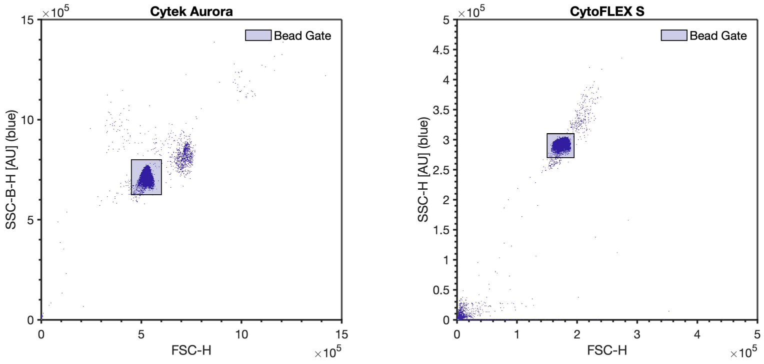

QbSure beads gated on blue SSC-H and FSC-H on the Cytek Aurora and CytoFLEX S. The violet SSC settings remain unchanged. In order to limit .fcs file size, a FSC scatter trigger may be used until 10000 QbSure bead events are acquired.

Note

You may need to adjust the B-SSC and FSC detector settings for the bead acquisition. Do not adjust fluorescence detector settings or high sensitivity SSC detector settings. Assuming the QbSure beads were cross calibrated in the previous protocols, this allows for the calibration of the rEV fluorescent data into calibrated units.

16 If calibration of data into standard units is desired, the 100 nm polystyrene NIST bead and QbSure beads can be used to calibrate data by following the FCM_{PASS} experiment calibration protocols.

Protocol

NAME

FCMPASS Protocol Collection

CREATED BY

Joshua A Welsh

PREVIEW

G. Methods S7: Flow cytometer rEV immunophenotyping, related to Figure 1.

Resource 7: rEV immunophenotyping

In 1 collection

Sean M Cook[1], Vera A. Tang[2], Joanne Lannigan[3], Jennifer Jones[1], Joshua A Welsh[4]

[1]Laboratory of Pathology, Translational Nanobiology Section, Centre for Cancer Research, National Institute of Health, National Institutes of Health; [2]University of Ottawa; [3]Flow Cytometry Support Services; [4]Translataional Nanobiology Section, Laboratory of Pathology, Center for Cancer Research, National Cancer Institute, National Institutes of Health

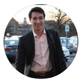

Sean M Cook

Protocol Info: Sean M Cook, Vera A. Tang, Joanne Lannigan, Jennifer Jones, Joshua A Welsh . Resource 7: rEV immunophenotyping. **protocols.io** https://protocols.io/view/resource-7-rev-immunophenotyping-cnjsvcne

Created: Jan 30, 2023

Last Modified: Mar 25, 2023

PROTOCOL integer ID: 76114

DISCLAIMER

This protocol summarizes key steps for a specific type of method, which is one of a collection of methods and assays used for EV analysis in the NCI Translational Nanobiology Section at the time of submission of this protocol. Appropriate use of this protocol requires careful, cohesive integration with other methods for EV production, isolation, and characterization.

MATERIALS

Exosome standards fluorescent **Merck MilliporeSigma (Sigma-Aldrich) Catalog #SAE0193**
, referred to as 'rEVs'

DPBS **Thermo Fisher Scientific Catalog #14190144** , referred to as 'DPBS'

5 mL Round-bottom tube **Corning Catalog #352052** , referred to as 'FACS tube'

Protein LoBind Tubes (5 mL) **Thermo Fisher Scientific Catalog #0030122356**
, referred to as 'low-binding Eppendorf tubes'

Low Protein Binding Microcentrifuge **Thermo Fisher Scientific Catalog #88379**

96-well V bottom plate **Contributed by users**

PE anti-human CD81 (TAPA-1) Antibody **BioLegend Catalog #349505** , referred to as 'anti-CD81-PE'

APC anti-human CD81 (TAPA-1) Antibody **BioLegend Catalog #349509** , referred to as 'anti-CD81-APC'

Pacific Blue™ anti-human CD81 (TAPA-1) Antibody **BioLegend Catalog #349515**
, referred to as anti-CD81-PB'

QbSure **Cytek Catalog #B7-10005** , referred to as 'QbSure'

100 nm polystyrene NIST bead **Thermo Fisher Scientific Catalog #3100A**
, referred to as '100 nm NIST bead'

Sample Preparation

35m

1 Briefly centrifuge the rEVs 100 x g, 4°C, 00:05:00 before opening

5m

2 Add 100 µL of 4 °C deionized water. Pipette up and down to mix.

Note

Do not vortex

3 Dilute the reconstituted rEVs 1 in 5 in PBS for staining* Take 50 µL of the rEV stock and add

200 µL of PBS.

Note

The sample concentration chosen for staining should take into consideration the need to dilute the sample further after staining to reduce the concentration of free antibodies, since the samples will be acquired without a washing step to remove these antibodies as in the case of cells.

4 Prepare the antibody dilutions for a 2x staining concentration of each of the antibodies to be used in the antibody titration. See the example below for sample calculations prepared for a staining concentration of 2 µg/ml.

A	B	C	D	E	F
	AB stock concentration (µg/ml)	2x staining conc (µg/ml)	**Stock volume for 2x staining conc (µL)**	Total volume for 2x staining conc (µL)	**Volume of PBS to be added (uL)**
anti-Human CD81-PE	120	4	**2**	60	**58**
anti-Human CD81-APC	200	4	**2**	100	**98**
anti-Human CD81-PB	300	4	**2**	150	**148**

Sample calculations prepared for a staining concentration of 2 µg/ml. All Antibodies clones are 5A6.

5 Samples will now be prepared using the following plate map.

A	B	C	D	E	F	G	H
	**CD81 PE**	**CD81 APC**	**CD81 Pacific Blue**	**CD81 PE**	**CD81 APC**	**CD81 Pacific Blue**	
**Sample**	rEV	rEV	rEV	PBS	PBS	PBS	
**0 µg/mL**	A1	A2	A3	A4	A5	A6	
**0.0625 µg/mL**	B1	B2	B3	B4	B5	B6	
**0.125 µg/mL**	C1	C2	C3	C4	C5	C6	
**0.25 µg/mL**	D1	D2	D3	D4	D5	D6	
**0.5 µg/mL**	E1	E2	E3	E4	E5	E6	
**1 µg/mL**	F1	F2	F3	F4	F5	F6	

	A	B	C	D	E	F	G	H
	**2 µg/mL**	G1	G2	G3	G4	G5	G6	**QbSure Beads**
		PBS	PBS	PBS	PBS	PBS	PBS	**100 nm PS NIST-Traceable Beads**

6 In a 96-well V-bottom plate, add 10 µL 1:5 rEV solution to wells A1-A3

7 Add 10 µL DPBS to wells A1-A3 and reverse pipet to mix. These wells will serve as the rEV controls

8 Add 20 µL DPBS to wells A4-A6. These wells will serve as buffer only controls

9 In the same 96-well V-bottom plate from go to step #6, add 10 µL 1:5 rEV solution to wells B1-G3. Add 10 µL DPBS to wells A1-G1. Add 10 µL from the PE working solution tubes to wells B1-G1, add 10 µL from the APC working solution tubes to wells B2-G2, add 10 µL from the PB working solution tubes to wells B3-G3.

10 In the same 96-well V-bottom plate from go to step #6, add 10 µL from the corresponding PE working solutions tube to wells B4-G4. Add 10 µL from the corresponding APC working solutions tube to wells B5-G5. Add 10 µL from the corresponding PB working solutions tube to wells B6-G6. These wells will serve as the DBPS+AB control wells.

11 Cover and incubate this plate for 00:30:00 at RT. 30m

12 Using a separate 96-well V-bottom plate, add 199 µL DPBS into wells A1-G6. Pipet 1 µL from the incubation plate into the new plate in the same wells and reverse pipette to mix.

Note

Steps 11 and 12 will be repeated for the ON incubations as well to bring rEVs down to running concentration that doesn't swarm the instrument detectors.

13 Add 200 µL DPBS into wells A7-G7. These wells will help reduce sample carryover after each rEV+AB combination is acquired.

14 Re-cover incubation plate and let incubate ON at RT to repeat measurements next day as directed in the following steps.

15 Label as FACS tube as 'QbSure', and add 500 µL DPBS. Vortex the QbSure beads for 5 sec and add 3 drops QbSure beads into FACS tube.

Cytometer Setup

16 Setup cytometer to acquire at FCM_{PASS} output gains for both light scatter and fluorescent detectors as determined by the FCM_{PASS} detector optimization module outputs. For the Aurora, an optimal gain template is returned to import into the Spectraflo software.

17 On the Cytek Aurora, set window extension to 0. On the CytoFLEX platform turn on 'High Acquisition Mode'.

Note

To set the window extension to 0 on the Cytek Aurora, navigate to the 'Lasers' tab under the sample acquisition settings and set the window extension to 0.

To change the acquisition mode on the CytoFLEX S, click on the 'Advanced' menu on the top-of-the-screen ribbon. Next click on 'Event Rate Settings' menu, change the acquisition mode to 'High', and press 'Ok'.

18 Set the cytometer triggering threshold to the violet SSC parameter and run a DPBS control first to ensure the cytometer is clean and thresholds/event rates have remained unchanged. The

background event rate should be ~1000 events/sec. **All samples should be acquired with the lowest flow rate, typically ~10-15 µL min^{-1}.**

19 Acquire all wells for at least 60 sec.

20 In an open well, run the 100 nm polystyrene NIST beads at the same settings as the rEVs (diluted in DPBS to a concentration of 5E6 p/mL) until at least 10000 bead events have been acquired.

21 In an open well, add 200 µL from the QbSure FACS tube and collect 10,000 bead events at the same settings as the rEVs.

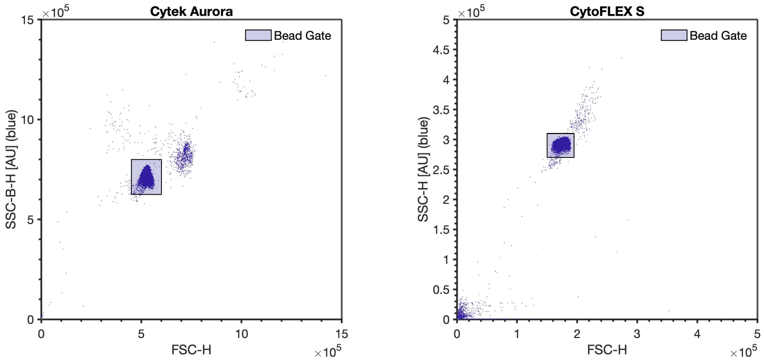

QbSure beads gated on blue SSC-H and FSC-H on the Cytek Aurora and CytoFLEX S. The violet SSC settings remain unchanged. In order to limit .fcs file size, a FSC scatter trigger may be used until 10000 QbSure bead events are acquired.

Note

You may need to adjust the B-SSC and FSC detector settings for the bead acquisition. Do not adjust fluorescence detector settings or high sensitivity SSC detector settings. Assuming the QbSure beads were cross calibrated in the previous protocols, this allows for the calibration of the rEV fluorescent data into calibrated units.

22 If calibration of data into standard units is desired, the 100 nm polystyrene NIST bead and QbSure beads can be used to calibrate data by following the FCM_{PASS} experiment calibration protocols.

Protocol

NAME

FCMPASS Protocol Collection

CREATED BY

Joshua A Welsh

PREVIEW

23 Don't forget to repeat the acquisition for the samples incubating ON.

References
