# Supplementary material for: Quantitative flow cytometry enables end-to-end optimization of cross-platform extracellular vesicle studies
Source: Cell Rep Methods. 2023 Dec 18;3(12):100664. doi: 10.1016/j.crmeth.2023.100664 (PMC10753385; doi:10.1016/j.crmeth.2023.100664)
Supplement: Document S2. Article plus supplemental information [file mmc2.pdf]

Cell Reports Methods

Article

Quantitative flow cytometry enables end-to-end optimization of cross-platform extracellular vesicle studies

Graphical abstract

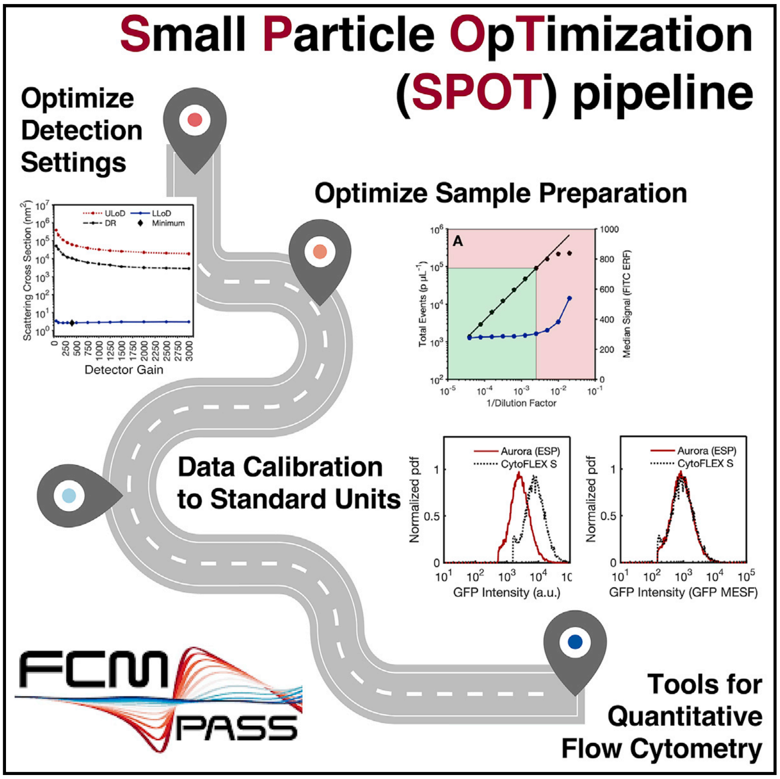

Highlights

- The SPOT pipeline optimizes quantitative flow cytometry of extracellular particles
- SPOT facilitates standardized cross-platform comparisons for qFCM
- SPOT enables optimization of fluorophore selection and laser and detector settings

Authors

Sean Cook, Vera A. Tang, Joanne Lannigan, Jennifer C. Jones, Joshua A. Welsh

Correspondence

joadwe@outlook.com

In brief

Cook et al. demonstrate the utility of flow cytometer detector setting optimization and calibration for small-particle analysis and cross-platform comparisons.

Cook et al., 2023, Cell Reports Methods *3*, 100664
December 18, 2023 © 2023
https://doi.org/10.1016/j.crmeth.2023.100664

CellPress

Cell Reports Methods

CellPress
OPEN ACCESS

Article

Quantitative flow cytometry enables end-to-end optimization of cross-platform extracellular vesicle studies

Sean Cook,[1] Vera A. Tang,[2] Joanne Lannigan,[3] Jennifer C. Jones,[1,4] and Joshua A. Welsh[1,4,5,*]
[1]Laboratory of Pathology, Translational Nanobiology Section, Centre for Cancer Research, National Institute of Health, National Institutes of Health, Bethesda, MD, USA
[2]Faculty of Medicine, Department of Biochemistry, Microbiology, and Immunology, University of Ottawa, Flow Cytometry and Virometry Core Facility, Ottawa, ON K1H 8M5, Canada
[3]Flow Cytometry Support Services, Alexandria, VA, USA
[4]Senior author
[5]Lead contact
*Correspondence: joadwe@outlook.com
https://doi.org/10.1016/j.crmeth.2023.100664

MOTIVATION Small-particle flow cytometry is increasingly being utilized on instruments primarily developed for cellular analysis. To date, there has been a lack of instrument agnostic methods to characterize and optimize flow cytometer performance for small-particle applications.

SUMMARY

Flow cytometry (FCM) is a common method for characterizing extracellular particles (EPs), including viruses and extracellular vesicles (EVs). Frameworks such as MIFlowCyt-EV exist to provide reporting guidelines for metadata, controls, and data reporting. However, tools to optimize FCM for EP analysis in a systematic and quantitative way are lacking. Here, we demonstrate a cohesive set of methods and software tools that optimize FCM settings and facilitate cross-platform comparisons for EP studies. We introduce an automated small-particle optimization (SPOT) pipeline to optimize FCM fluorescence and light scatter detector settings for EP analysis and leverage quantitative FCM (qFCM) as a tool to further enable FCM optimization of fluorophore panel selection, laser power, pulse statistics, and window extensions. Finally, we demonstrate the value of qFCM to facilitate standardized cross-platform comparisons, irrespective of instrument configuration, settings, and sensitivity, in a cross-platform standardization study utilizing a commercially available EV reference material.

INTRODUCTION

Flow cytometers were first developed in the 1960s for fluorescence-based detection of cells.[1] Today, their design is still primarily focused on cellular phenotyping but with increased throughput and multi-dimensionality.[2,3] As the use of flow cytometry (FCM) as a technique has become common place in research institutions, alternative applications have been explored. Today, FCM is increasingly being utilized to characterize sub-micron particles in the form of extracellular particles (EPs), which include extracellular vesicles (EVs) and viruses.[4–8] These particles are orders of magnitude smaller and dimmer than most FCM equipment was originally intended to characterize.

As utilization of FCM for EP analysis has increased so too has the awareness of its limitations and the lack of reproducibility and validity of published data.[9–11] In 2020, the MIFlowCyt-EV framework was published as a position paper delineating minimal reporting standards for EV FCM.[12] This work was a product of a 5-year collaboration of international researchers from the International Society of Extracellular Vesicles (ISEV), the International Society for Advancement of Cytometry (ISAC), and the International Society for Thrombosis and Haemostasis (ISTH), which formed an intersocietal EV FCM working group in 2015. This reporting framework outlines key metadata, controls, calibration, and data reporting fields that should be completed when undertaking small-particle measurements using FCM. While reporting criteria have been established, there is an unmet need for FCM tools to facilitate increased reproducibility and validity of EPs that will in turn aid in the reliability and correct interpretation of published data.

This is an open access article under the CC BY license (http://creativecommons.org/licenses/by/4.0/).

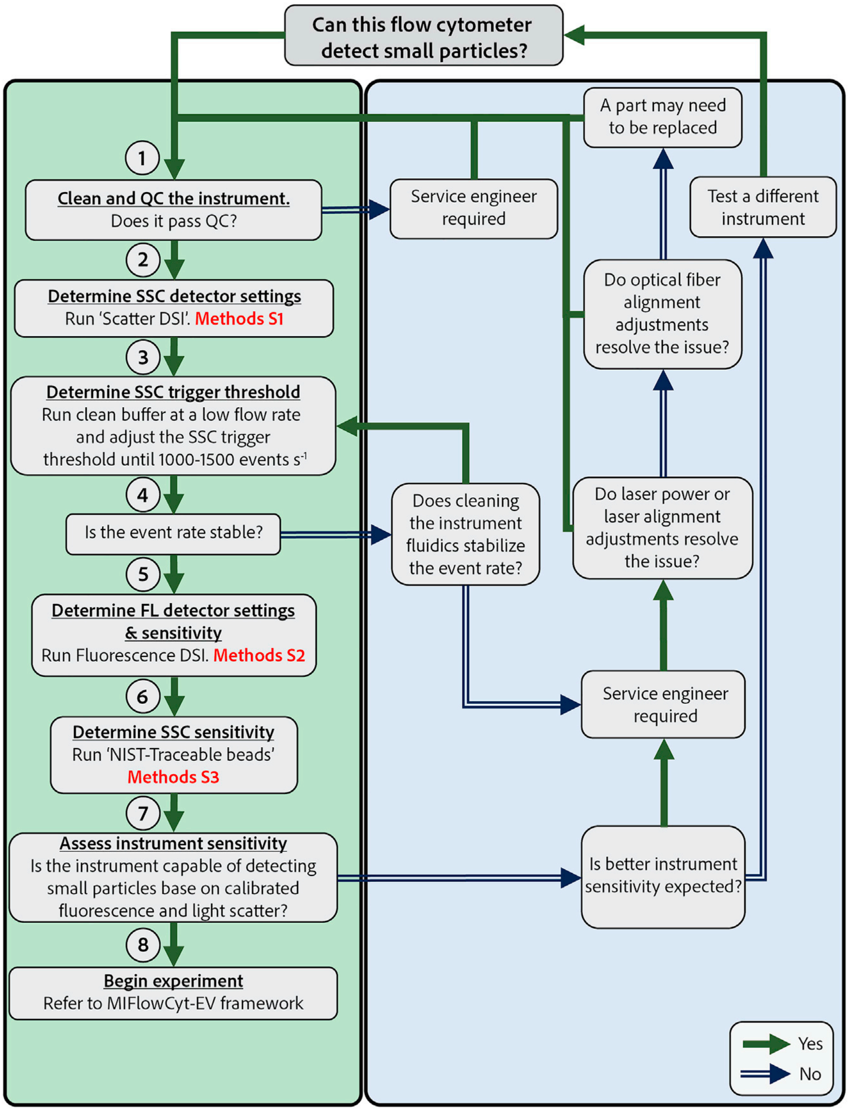

Figure 1. Decision tree for assessing the suitability of a flow cytometer for small-particle analysis

Robust detection of EPs using FCM requires systematic and rigorous optimization of flow cytometer configuration and settings. Furthermore, the characterization of EPs themselves requires quantitative metrics to enable longitudinal utility and intrainstrument comparisons. In order to address these outstanding requirements in the field, we developed a small-particle optimization (SPOT) pipeline utilizing quantitative FCM (qFCM; Figure 1).

In the small-particle FCM field, the term ''calibration'' has been interpreted in multiple ways. Commonly, simply analyzing beads and drawing gates between the populations has been marketed and published in the small-particle field as "calibration" and has been the product of early standardization initiatives.[13,14] Here, we introduce qFCM as a term for the conversion of arbitrary units (a.u.) to standard units in order to avoid confusion with existing literature and reagent implementations. qFCM is essential for EP FCM, owing to commercially available equipment being unable to detect the full distribution of EVs from complex biofluids such as plasma and cerebrospinal fluid. This limitation results in different equipment detecting different portions of the same population, and without quantitating the data in standard units, comparison between platforms become infeasible, limiting the utility of data. Here, we not only demonstrate the ability of qFCM to characterize instrument performance and enable optimization of the instrument, but we also demonstrate that qFCM facilitates cross-platform comparisons irrespective of instrument platform, configuration, and settings.

All flow cytometers have daily quality control measures that track longitudinal performance. However, these measures are sub-optimal for small-particle analysis, as they have been developed for cellular analysis, which has different optimization considerations and requirements. Protocols currently developed for optimization of the cellular detector settings commonly derive the ''minimum detector settings.''[15] These settings are derived to minimize electronic noise while maximizing dynamic range. Furthermore, these settings are based on the acquisition of large, bright beads that have hundreds of thousands of copies of a given fluorophore on their surface. Utilizing qFCM, we developed the SPOT pipeline to automate the derivation of optimal small-particle fluorescence and light scatter detection settings in order to maximize the sensitivity of the FCM platform and derive instrument sensitivity in quantitative units. We go on to validate the performance of these derived settings using beads with commercially available EV reference materials.

RESULTS

qFCM enables cross-platform comparisons

To quantitatively characterize the performance of an instrument, a standard metric for the parameters measured must be identified. The light detected in FCM in the form of scatter (SSC) and fluorescence (FL) is reported in a.u. and does not allow for direct comparisons between instruments. Due to the broad range in cellular epitope abundance, detector optimization methods to date have focused on the minimal setting to reduce the effects of electronic noise on the detection of cells.[15] These methods use a bright bead population(s) and identify the detector setting by using the inflection point of the detector setting versus the coefficient of variation.

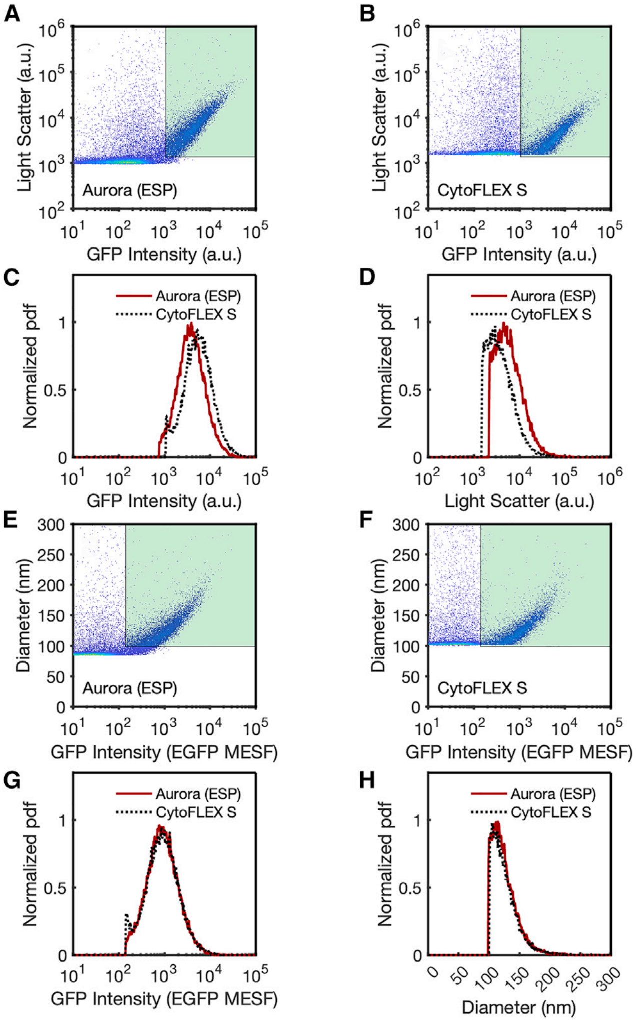

Figure 2. Fluorescence and light scatter calibration allow EV intra-instrument comparisons

(A) rEVs acquired on the Aurora platform plotted in arbitrary units.

(B) rEVs acquired on the CytoFLEX platform plotted in arbitrary units. The green region denotes the gated area of the CytoFLEX platform in arbitrary units.

(C) Comparison of GFP intensity in arbitrary units from gated (green region in plot A-B) Aurora (solid red line) and CytoFLEX S (dotted black line) data.

(D) Comparison of SSC intensity in arbitrary units from gated (green region in plot A-B) Aurora (solid red line) and CytoFLEX S (dotted black line) data.

(E) rEVs acquired from the Aurora platform plotted in calibrated units.

(F) rEVs acquired from the CytoFLEX platform plotted in calibrated units. The green region denotes the gated area of the CytoFLEX platform in calibrated units.

(G) Comparison of GFP intensity in calibrated units from gated (green region in plot E-F) Aurora (solid red line) and CytoFLEX S (dotted black line) data as normalized probability distribution functions (pdfs).

(H) Comparison of SSC intensity in calibrated units from gated (green region in plot A-B) Aurora (solid red line) and CytoFLEX S (dotted black line) data.

Since the signal intensities associated with cells are well within the range of detection for commercial flow cytometers, the quantitative characterization of the limits of detection for these instruments is not prioritized for the majority of FCM users. Using qFCM to derive limits of detection has been necessitated by the fact that current commercially available flow cytometers lack the sensitivity to detect the full distribution of EVs from complex biofluids such as plasma and cerebrospinal fluid. In this application, it becomes important for the limit of detection to be quantitatively defined and optimized for each instrument to allow for the greatest detection of the EV population being characterized. Therefore, utilizing qFCM, rather than a.u. FCM, is necessary for comparisons to be made between platforms. To illustrate this, the analysis of recombinant EVs (rEVs) expressing EGFP, a commercially available reference material, is shown on two flow cytometers as reported in a.u. (Figures 2A and 2B). A gate using the limits of detection from the CytoFLEX cytometer in a.u. was applied to the data of the same sample collected on the Aurora platform. When comparing the gated data, shown in Figures 2C and 2D, there is poor concordance between the populations from the same rEV sample analyzed by the CytoFLEX and Aurora on both fluorescence and light scatter. The uncalibrated fluorescence intensities on the CytoFLEX and Aurora were 5.4×10^3 and 3.8×10^3 a.u., and the uncalibrated light scatter intensities were 3.5×10^3 and 5.4×10^3 a.u., respectively. Upon calibrating the EGFP intensity from a.u. to molecules of equivalent soluble fluorophore (MESF) and the light scatter intensity to units of diameter in nanometers and then gating the populations (Figures 2E and 2F), concordance is greatly improved between platforms. Calibrated data between the CytoFLEX and Aurora platforms had median fluorescence intensities of 882 and 875 EGFP MESF (Figure 2G), and diameters of 120.8 and 120.3 nm (Figure 2H), respectively. These data demonstrate that irrespective of instrument platform, sensitivity, and settings, data can be compared when qFCM is utilized, whereas direct comparisons between a.u. FCM data cannot be made.

Identifying flow cytometer limits of detection and optimizing detector settings

When data are acquired on a flow cytometer, they are scaled on a.u. axes. While the number of photons reaching the detector can be constant, altering the detector settings amplifies the photons that have reached the detector and transposes the data up and down this arbitrary axis. To date, optimizing and maintaining detector settings for cellular analysis has typically been achieved by assessing the coefficient of variation (CV) in a reference bead population and identifying the detector setting at which the bead fluorescence variation plateaus (Figure 3A).[15] Due to cells having orders of magnitude more epitopes for labeling than EPs, dynamic range is a concern that must be balanced. Full separation of positive versus negatively stained cells can therefore be achieved using detector settings with reduced sensitivity or by titrating antibodies, resulting in dimmer stained populations. The requirements for small, dim particles are not the same for those of cells. Due to many EP derivations having a log-normal distribution with a modal point $\lesssim$100 nm and having <50 copies of any given epitope, detector sensitivity must be maximized due to the majority of EPs being undetectable. The use of minimum CV for a bright bead population is not sufficient for identifying optimal settings for dim signals, as the detector settings at which the minimum CV is

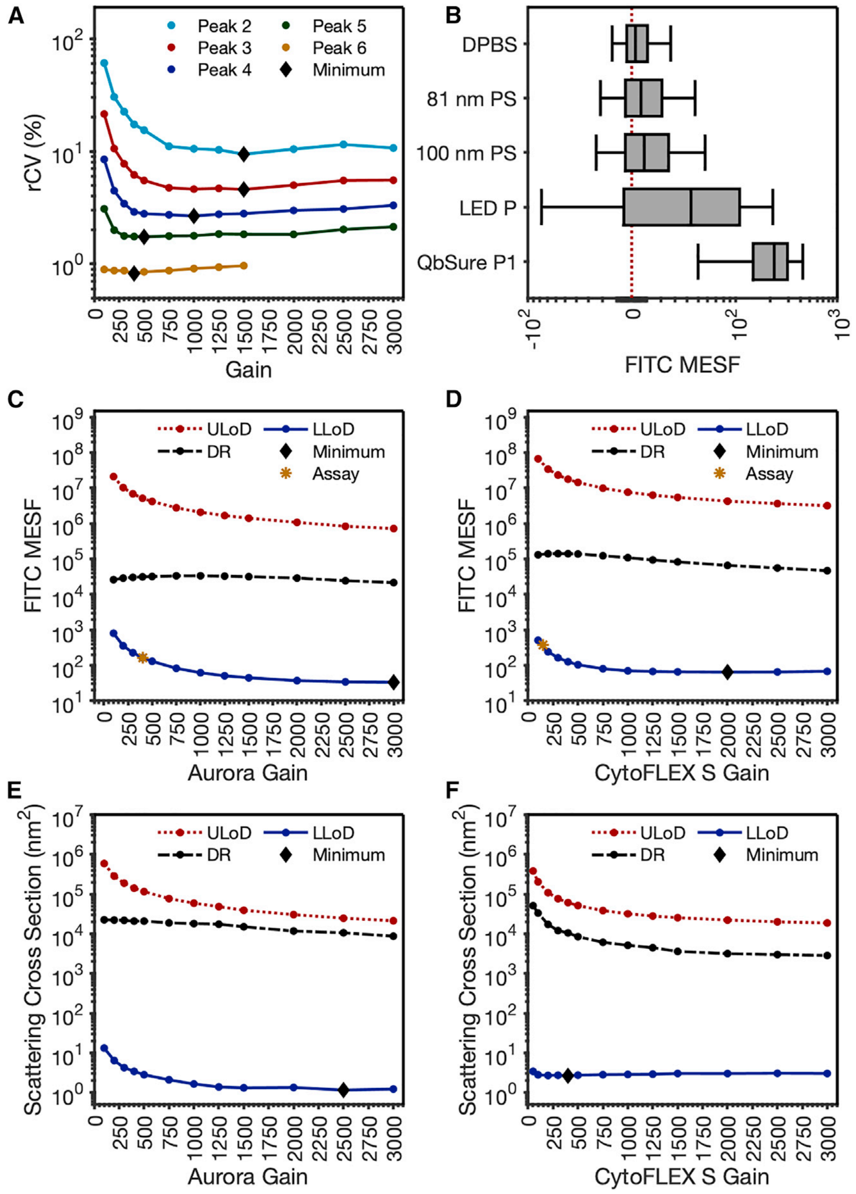

Figure 3. Development of a small-particle detector setting optimization methodology

(A) Percentage of CV of QbSure rainbow multi-peak bead populations across a range of detector settings on the Aurora. The minimum percentage of CV per population is denoted by a black diamond.

(B) Comparison of different methods for quantification of the lower limit of detection (LLoD) on the Aurora platform. Red dotted line indicates 0.

(C and D) Aurora (C) and CytoFLEX (D) LLoD, upper LoD (ULoD), and dynamic range (DR) default instrument settings (Assay) of their FITC channel as a function of gain setting using FCM_{PASS} fluorescent detector optimization protocol.

(E and F) Aurora (E) and CytoFLEX (F) scatter LLoD, ULoD, and DR as a function of gain setting using FCM_{PASS} light scatter detector optimization protocol.

reached are dependent on how bright the signal is (Figure 3A). This is demonstrated with a set of 6-peak rainbow beads, where the minimum CV for the brightest population is reached at a gain of 400, while the second dimmest population reaches a minimum CV at a gain of 1,500.

The criteria for optimizing detector settings for EPs are different than those for cells. With EPs, full separation of positively and negatively stained populations is often unachievable due to sensitivity limitations. With limitations in sensitivity and the lower abundance of epitopes, dynamic range is less often a concern when optimizing detector settings for relatively small signals. Therefore, the most sensitive detector settings on photon multiplier tubes (PMTs) and avalanche photodiode (APD)-based flow cytometers tend to have sufficient sensitivity to detect a portion of EPs. Most critically, the derivation of optimal detector settings for small particles relies on having the ability to determine an instruments lower limit of detection (LoD). Commonly in spectrometric assays, this is derived by having a "blank" control. Typically, FCM reference materials are in the form of beads due to low cost, stability, and ease of use, and have tunable properties such as size, fluorescence, and refractive index (RI).

Multi-peak rainbow beads (QbSure, ~3 μm), polystyrene nanoparticles (81, 100 nm), an LED pulser, and instrument opto-electronic noise were compared to understand their fluorescence intensity and distribution (Figure 3B). When compared, instrument opto-electronic noise had the lowest 95th percentile intensity of 27 fluorescein isothiocyanate (FITC) MESF, with the dimmest rainbow peak bead having the highest 95th percentile intensity at 421 MESF (statistics summarized in Table S1). All distributions when tested for normality had significantly nonnormal distribution with various levels of skewness (Table S1). From this, we have demonstrated that triggering on opto-electronic noise using Dulbecco's phosphate buffered saline (DPBS) as a sample is the most reliable method of determining the instrument's lower LoD.

By normalizing the opto-electronic noise as a reference population across detector setting incrementation (DSI) either using qFCM or normalizing to a bright fluorescent particle that is detectable at all gains, it is possible to identify the settings at which the instrument has the greatest sensitivity for fluorescence (Figures 3C and 3D) and light scatter (Figures 3E and 3F). It is notable that maximal sensitivity on the Aurora and CytoFLEX platforms for fluorescence and light scattering was not always achieved simply by increasing the detectors to their maximum settings. This analysis procedure, along with automated bead

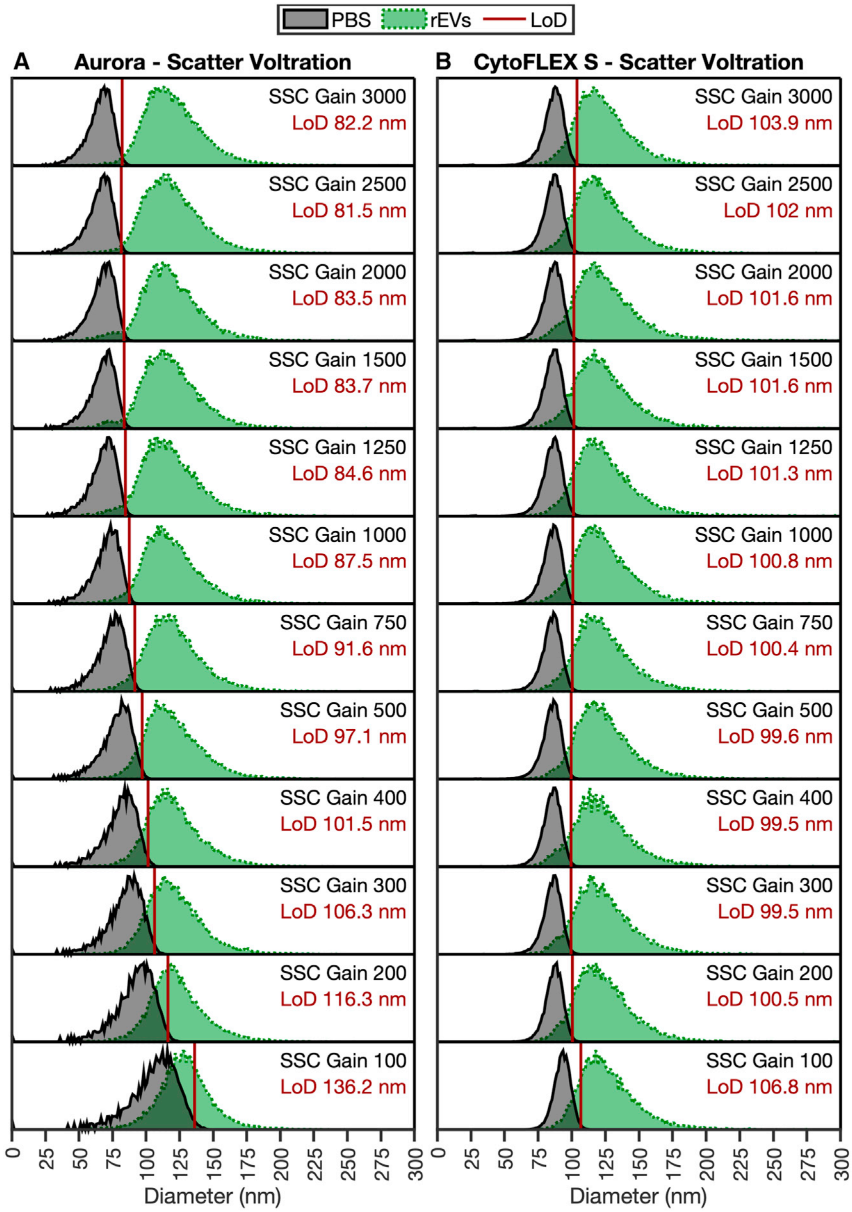

Figure 4. 405 nm light scatter detector setting incrementation

Probability density functions of buffer (gray) and rEVs (green) collected on the (A) Aurora and (B) CytoFLEX S at increasing detector settings. Probability density functions are normalized to 1 at the modal point of each distribution. The LLoD (solid red line) is specified in nm in the top right of the distribution plot and is calculated as the 99th percentile of the buffer control. rEVs data were acquired at ~5 × 10^6 particles mL^{-1} diluted in PBS. The optimal gain occurs at the lowest LoD. rEV diameter assumes a core-shell structure with a shell thickness of 5 nm, an RI of 1.486, and a core RI of 1.42.

cross-calibration, was built into an automated peak gating and analysis function within FCM_{PASS} software to allow ergonomic assessment of optimal instrument detector settings and sensitivities in calibrated units. Currently, commercial flow cytometers only optimize fluorescent detector settings for fluorescence. Here, we show that compared to default cellular settings, we achieved a 4.91-fold increase in sensitivity on the Aurora platform and a 5.80-fold increase on the CytoFLEX platform using the SPOT pipeline.

Validating optimized detector settings on EV detection

To validate the utility of the SPOT pipeline for biological particles, detector setting incrementation (DSI) using reference EVs was acquired across light scatter (Figure 4) and fluorescent (Figure 5) detector settings on two different instrument platforms. Despite both cytometers using APD detectors, there are notable differences in the results. The change in sensitivity was larger between settings on the Aurora (Figure 4A) than the CytoFLEX S (Figure 4B), with a total change in sensitivity from least to most sensitive being 136.2 to 81.5 nm, whereas the CytoFLEX S had a total range of 106.8 to 99.5 nm. Notably, in both cases, the most sensitive settings light scatter detectors did not have the highest detector gain. The light scatter detector settings derived as optimal using the SPOT pipeline are in agreement with the EV validation in Figures 3E and 3F, whereby peak sensitivity for the Aurora platform was obtained at a detector gain setting of 2,500 and the CytoFLEX S at 400.

In the case of fluorescence detector settings, sensitivity increased, due to the lower LoD decreasing from 734 to 42 FITC equivalent reference fluorophore (ERF), with peak sensitivity (lowest LoD) at a setting of 2,500. On the CytoFLEX S, sensitivity increased from 754 to 72 ERF. The derived detector settings using the SPOT pipeline and validation with EVs are in agreement. As indicated by bead-based detector setting analysis (Figure 3D), the highest gain on the CytoFLEX S does depreciate the sensitivity of the signal from peak sensitivity by ~10%. These results indicate that the use of opto-electronic noise and the developed SPOT pipeline are capable of identifying optimal detector settings for small-particle analysis without the use of biological samples (Figures 3C–3F). The derivation of these settings is multi-factorial, and at a minimum, the SPOT pipeline should be repeated when any changes are made to the system's electronics, fluidics, or optics, i.e., after a preventative maintenance visit. Periodic implementation is, however, recommended to ensure flow cytometer performance consistency over time.

Optimized instrument configuration on EV detection

Beyond detector settings, further modifications can be made to potentially increase the detection sensitivity of flow cytometers

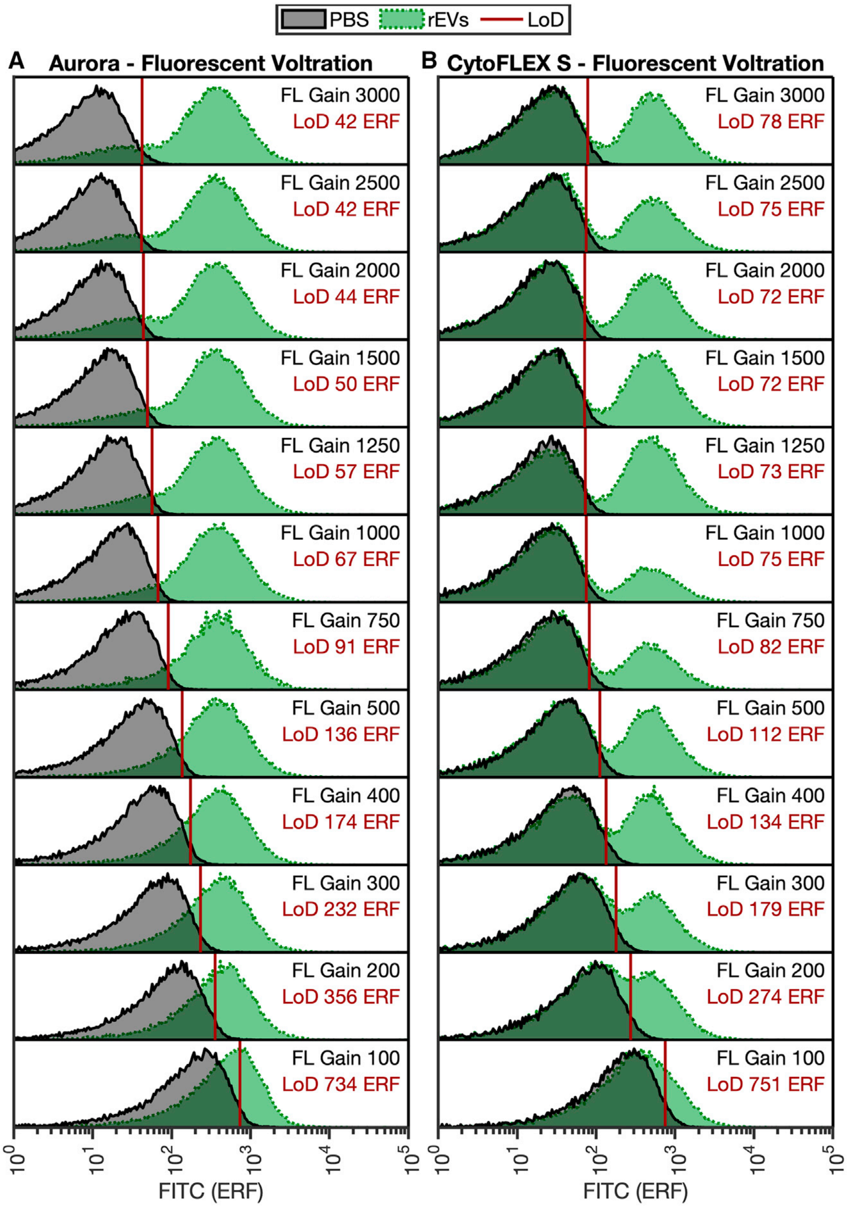

Figure 5. Fluorescence detector setting incrementation

Probability density functions of buffer (gray) and rEVs (green) collected on the (A) Aurora and (B) CytoFLEX S at increasing detector settings. Probability density functions are normalized to 1 at the modal point of each distribution. The LLoD (solid red line) is specified in FITC ERF in the top right of the distribution plot and is calculated as the 99th percentile of the buffer control. rEVs data were acquired at ~5 × 10^{6} particles mL^{-1} diluted in PBS. The optimal gain occurs at the lowest LoD.

for signal quantification. These include the laser powers and window extensions. The effect of laser power on rEV detection was measured by increasing the power by 25 mW from 50 to 150 mW using optimal detector settings derived from the FCM$_{PASS}$ DSI protocol (Figures 6A–6D). When calibrated units are utilized, the sensitivity increased from 43 to 24 ERF (Figure 6A). When visualized using a.u. (Figure 6B), the small gain in sensitivity from 100 to 150 mW is difficult to visualize, as both the noise and rEV populations are increasing with laser power. Figure 6C shows that, while the median fluorescence intensity of the rEV population is maintained, the signal-to-noise ratio continues to increase, signified by the linear reduction in the DPBS LoD population. Furthermore, when observing the median rEV statistics and the DPBS LoD (Figure 7D), the increase in rEV signal forms a hyperbolic response that is beginning to plateau, while the LoD linearly increases. This indicates that further increases in laser power will result in a diminished signal-to-noise ratio, as, eventually, the EGFP will saturate and be unable to emit anymore photons. Laser power versus fluorescence intensity can also be viewed on a linear plot in Figure S1.

Electronic acquisition settings, as well as detector and laser settings, can impact the sensitivity of the instrument. Most flow cytometers by default have a window extension (WE) of ~5 μs. The WE is designed for cellular analysis and can increase the sensitivity for events that are larger than the laser beam height. The laser beam height on Aurora and CytoFLEX S platforms is ~5 μm and is therefore far larger than most biological EPs. By decreasing the WE from 6 to 0 μs, sensitivity increased based on the fluorescence area statistic on the Aurora by 2.9-fold from 142 to 49 FITC ERF for rEVs. When using the fluorescence height statistic, the change in lower LoD (LLoD) was not as pronounced, decreasing from 90 to 70 FITC ERF, a 1.3-fold increase in sensitivity. Similarly, switching from "default" to "high acquisition mode" on the CytoFLEX S increased fluorescence area sensitivity 1.8-fold from 73 to 41 FITC MESF, while the height sensitivity increased from 113 to 98 FITC ERF (Figure 6E). In the case of both cytometers, when WE settings were optimized for small-particle detection, the fluorescence area statistic resulted in a higher sensitivity value than the height statistic.

qFCM enables concordance in cross-platform interlaboratory small-particle studies

To investigate the utility of the FCM$_{PASS}$ DSI protocol developed and the reproducibility of biological data acquired when optical signals were calibrated, a small cross-platform study was undertaken with four Aurora (Figures 7A–7D) and four CytoFLEX S (Figures 7E–7H) platforms. In all cases, when instruments were

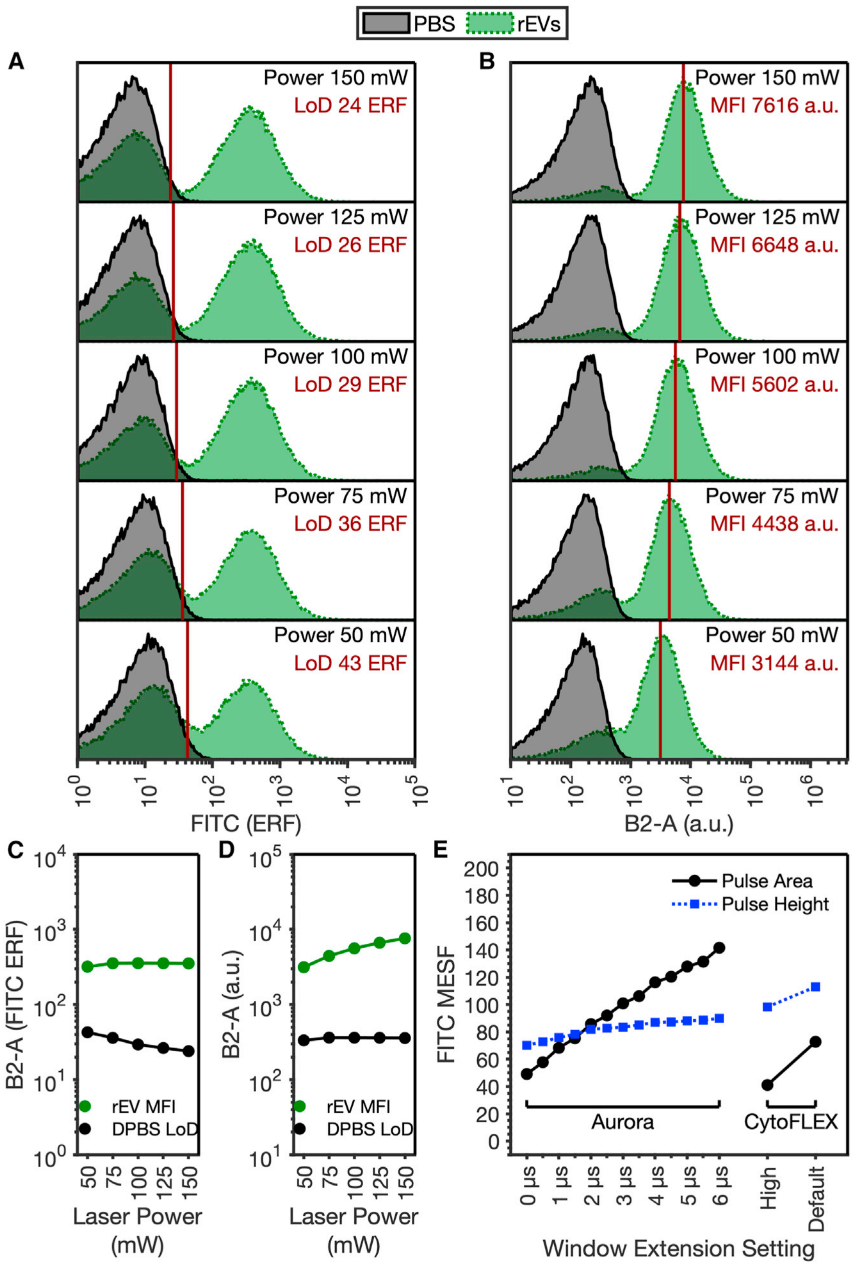

Figure 6. Laser power and window extension optimization

(A and B) Probability density function overlays of PBS and rEV populations at varying laser powers on the Aurora platform in (A) FITC ERF units and (B) arbitrary units.

(C and D) Summary statistics of PBS and rEV populations at varying laser powers on the Aurora platform in (C) FITC ERF units and (D) arbitrary units.

(E) LLoD of the area (black dots) and height statistics (blue dots) on Aurora and CytoFLEX S platforms with varying window extension settings. Window extension settings were altered in 1-μs increments on the Aurora from 0 to 6 μs, while the CytoFLEX S platform was compared using "default" and "high acquisition mode," as manual adjustments to window extensions are not supported.

optimized for small-particle analysis, the EGFP-tagged rEV population was detectable. These instruments ranged in fluorescence sensitivity from 29 to 155 EGFP MESF and light scatter sensitivity from 72 to 100 nm for EGFP-tagged rEVs (modeled with a shell thickness of 5 nm RI of 1.486 at 405 nm and a core RI of 1.42 at 405 nm). To investigate the concordance of data in a fair comparison, the rEV gate was drawn using the sensitivity of the least sensitive instruments. Events inside a gate with a diameter of 100–300 nm and an EGFP MESF intensity of 155 to 2×10^5 were compared by fluorescence (Figure 7I) and light scatter (Figure 7J). The median a.u. comparisons of data within the same gate resulted in a statistic ranging from 3,817 to 10,482 a.u. (175%) for fluorescence and from 3,114 to 10,858 a.u. (249%) for light scatter parameters. The use of qFCM resulted in highly concordant populations, with median statistics ranging from 838 to 1,054 EGFP MESF (~26%) and from 119.1 to 120.6 nm (~1%) for fluorescence and light scatter, respectively.

While the use of fluorescently tagged, commercially available reference materials is ideal for developing and validating optimal instrument settings for small-particle analysis, the numbers of fluorescently tagged molecules are at relatively high densities compared to the abundance of typical protein targets. As we have demonstrated using qFCM, there are likely an average of 800–1,000 copies of EGFP per rEV. Most often, FCM is utilized to measure the presence of surface epitopes, which are commonly present with less than 100 copies per particle. Detecting small numbers of surface proteins can be challenging due to having very weak signals. Not only do instrument detection parameters need to be finely tuned, but antibody fluorescent conjugates and concentrations also need to be thoughtfully carried out.

When comparing staining of identical clones of anti-CD81 antibodies conjugated to APC, PE, or Pacific Blue, anti-CD81-Pacific Blue yielded the lowest stain index, with a peak stain index of 0.63 using 30-min incubation and 0.60 using an overnight incubation at 2 μg mL^{-1}. Anti-CD81-PE and anti-CD81-APC with a 30-min incubation yielded peak stain indices of 2.00 and 2.18, respectively, at 1 μg mL^{-1}. At a higher antibody concentration of 2 μg mL^{-1}, the stain indices for both PE and APC conjugates were decreased to 1.83 due to the increase in unbound antibodies resulting in "swarm detection." Both anti-CD81-PE and anti-CD81-APC stain indices increased when using overnight incubation, with peak indices of 3.12 for PE and 2.68 for APC. While the peak stain index for

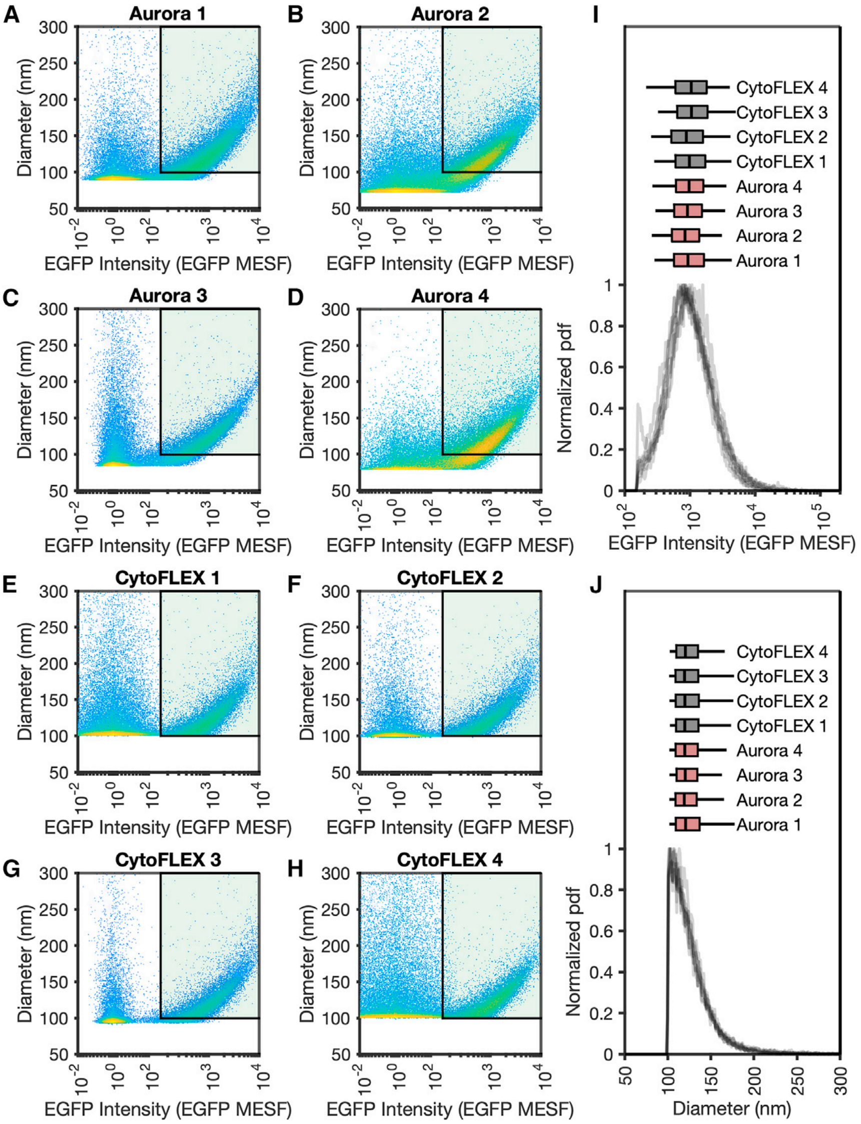

Figure 7. Cross-platform standardization

(A–H) rEVs were acquired at FCM_{PASS} DSI derived settings on four Aurora (A–D) and four CytoFLEX S (E–H) cytometers. A rEV gate (green box) was drawn that was based on the least sensitive EGFP MESF and diameter detectable by the cohort of instruments.

(I and J) Gated rEVs were overlaid for EGFP intensity (I) and diameter (J). Boxplots using the 5th, 25th, 50th, 75th, and 95th percentiles of each distribution were plotted for each individual cytometer above the distributions.

anti-CD81-PE was at 2 μg mL^{-1}, for anti-CD81-APC, this was at 1 μg mL^{-1} before decreasing to 2.27 at 2 μg mL^{-1} (Figures S2 and S3).

DISCUSSION

In this work we have demonstrated the SPOT pipeline to derive the most sensitive settings for FCM applications where small, dim particle analysis is required. We have demonstrated that the utilization of qFCM in assessing instrument detector sensitivity, laser powers, WEs, and pulse statistics enables both execution of SPOT to identify optimal small-particle FCM settings and reporting of data in standardized units. Furthermore, we have shown in a small cross-platform standardization study the ability of qFCM to generate highly concordant data, making cross-platform comparisons possible, facilitating the utility of commercially available instruments for the development of standardized assays. In addition to demonstrating a streamlined process for small-particle FCM instrument optimization, we have demonstrated the utility of this pipeline in the selection and optimization of conjugated antibody use for phenotyping of EVs. As well, we have validated the use of this approach to compare results across instruments, experiments, and institutions.

FCM_{PASS} was developed to be compatible with any commercial calibration reference materials for both fluorescence and light scattering calibration. Since its initial release, further improvements have been made to better support the needs of the field. The outputs of the FCM_{PASS} software now support automated export and completion of calibration materials, parameters, quality control, and hardware information for both MIFlowCyt and MIFlowCyt-EV reporting standards to enable transparent reporting in an ergonomic manner. The SPOT pipeline presented in this article provides a stepwise and streamlined way to leverage FCM_{PASS} tools to generate more robust and reproducible EV measurements.

While we have demonstrated the utility of commercially available instruments when optimized for small-particle detection, we have also demonstrated that these instruments are working close to their LoDs and beyond their intended specifications. Standard reference materials, software tools, and the methods presented herein are not only needed for researchers but also for manufacturers to improve the consistency and performance of instruments and instrument support, from manufacturing to benchtop performance.

Limitations of the study

While the developed pipeline has been tested on conventional and spectral cytometers along with on avalanche photodiode, photomultiplier tubes and silicon photomultiplier detectors, the pipeline is not applicable to imaging cytometers. The use of calibration on any flow cytometry platform assumes that the system has a linear response to input intensity. Some older generation instruments utilizing analogue log amplifiers which can be

non-linear in their signal:response may therefore may lack accuracy when calibrated.

STAR★METHODS

Detailed methods are provided in the online version of this paper and include the following:

- KEY RESOURCES TABLE
- RESOURCE AVAILABILITY
  - Lead contact
  - Materials availability
  - Data and code availability
- EXPERIMENTAL MODEL AND SUBJECT DETAILS
  - Ethics statement
- METHOD DETAILS
  - Instrument characterization using SPOT pipeline
  - Flow cytometry
  - Cross-calibration of ERF to MESF
  - Reference material preparation
  - rEV immunophenotyping
- QUANTIFICATION AND STATISTICAL ANALYSIS
  - Statistical analysis

SUPPLEMENTAL INFORMATION

Supplemental information can be found online at https://doi.org/10.1016/j.crmeth.2023.100664.

ACKNOWLEDGMENTS

J.A.W. is an International Society for Advancement of Cytometry (ISAC) Marylou Ingram Scholar (2019–2023). V.A.T. is an ISAC Shared Resource Laboratory Emerging Leader (2018–2023). The authors would like to thank early testers of the DSI features in FCM_{PASS}, which include Beckman Coulter, Cytek Biosciences, Brandy Pinckney and John Tigges (Beth Israel Deaconess Medical Center, Flow Cytometry Core), and Ionita Ghiran (Beth Israel Deaconess Medical Center). The authors would like to thank Beckman Coulter and Cytek Biosciences for providing access to instruments at the 2022 Annual Flow Cytometry Course for cross-platform comparison studies. S.C., J.C.J., and J.A.W. were supported by the Intramural Research Program of the NIH, NCI, Center for Cancer Research. J.C.J. acknowledges NIH ZIA BC011502 and ZIA BC011503.

AUTHOR CONTRIBUTIONS

J.A.W. and J.C.J. conceived of the pipeline. S.C., V.T., J.L., and J.A.W. collected the data and validated the tools. J.A.W. and V.A.T. developed analysis protocols. S.C. and J.A.W. developed the software tools. J.A.W. wrote the manuscript, created figures, and performed the data analyses. J.C.J. financially supported the study.

DECLARATION OF INTERESTS

J.A.W. and J.C.J. are inventors on NCI patents and patent applications related to EV analysis. J.L. holds a financial interest in Cytek Biosciences.

Received: April 18, 2023
Revised: July 28, 2023
Accepted: November 15, 2023
Published: December 8, 2023

REFERENCES

1. Fulwyler, M.J. (1965). Electronic separation of biological cells by volume. Science *150*, 910–911.
2. Park, L.M., Lannigan, J., and Jaimes, M.C. (2020). OMIP-069: Forty-Color Full Spectrum Flow Cytometry Panel for Deep Immunophenotyping of Major Cell Subsets in Human Peripheral Blood. Cytometry *97*, 1044–1051.
3. Ferrer-Font, L., Small, S.J., Lewer, B., Pilkington, K.R., Johnston, L.K., Park, L.M., Lannigan, J., Jaimes, M.C., and Price, K.M. (2021). Panel Optimization for High-Dimensional Immunophenotyping Assays Using Full-Spectrum Flow Cytometry. Curr Protoc *1*, e222.
4. Gąsecka, A., Pluta, K., Solarska, K., Rydz, B., Eyileten, C., Postula, M., van der Pol, E., Nieuwland, R., Budnik, M., Kochanowski, J., et al. (2021). Plasma Concentrations of Extracellular Vesicles Are Decreased in Patients with Post-Infarct Cardiac Remodelling. Biology *10*.
5. van der Pol, E., Coumans, F.A., Grootemaat, A.E., Gardiner, C., Sargent, I.L., Harrison, P., Sturk, A., van Leeuwen, T.G., and Nieuwland, R. (2014). Particle size distribution of exosomes and microvesicles determined by transmission electron microscopy, flow cytometry, nanoparticle tracking analysis, and resistive pulse sensing. J Thromb Haemost *12*, 1182–1192.
6. Burnie, J., Tang, V.A., Welsh, J.A., Persaud, A.T., Thaya, L., Jones, J.C., and Guzzo, C. (2020). Flow virometry quantification of host proteins on the surface of HIV-1 pseudovirus particles. Viruses *12*.
7. Tang, V.A., Renner, T.M., Varette, O., Le Boeuf, F., Wang, J., Diallo, J.S., Bell, J.C., and Langlois, M.A. (2016). Single-particle characterization of oncolytic vaccinia virus by flow virometry. Vaccine *34*, 5082–5089.
8. Tang, V.A., Renner, T.M., Fritzsche, A.K., Burger, D., and Langlois, M.A. (2017). Single-Particle Discrimination of Retroviruses from Extracellular Vesicles by Nanoscale Flow Cytometry. Sci. Rep. *7*, 17769.
9. Welsh, J.A., Holloway, J.A., Wilkinson, J.S., and Englyst, N.A. (2017). Extracellular Vesicle Flow Cytometry Analysis and Standardization. Front. Cell Dev. Biol. *5*, 78.
10. Welsh, J.A., Arkesteijn, G.J.A., Bremer, M., Cimorelli, M., Dignat-George, F., Giebel, B., Görgens, A., Hendrix, A., Kuiper, M., Lacroix, R., et al. (2023). A compendium of single extracellular vesicle flow cytometry. J. Extracell. Vesicles *12*, e12299.
11. Nolan, J.P., Chiu, D.T., and Welsh, J.A. (2022). Rigor and reproducibility: status and challenges for single vesicle analysis. Extracellular Vesicles and Circulating Nucleic Acids *3*, 244–248.
12. Welsh, J.A., Van Der Pol, E., Arkesteijn, G.J.A., Bremer, M., Brisson, A., Coumans, F., Dignat-George, F., Duggan, E., Ghiran, I., Giebel, B., et al. (2020). MIFlowCyt-EV: a framework for standardized reporting of extracellular vesicle flow cytometry experiments. J. Extracell. Vesicles *9*, 1713526.
13. Robert, S., Poncelet, P., Lacroix, R., Arnaud, L., Giraudo, L., Hauchard, A., Sampol, J., and Dignat-George, F. (2009). Standardization of platelet-derived microparticle counting using calibrated beads and a Cytomics FC500 routine flow cytometer: a first step towards multicenter studies? J Thromb Haemost *7*, 190–197.
14. Chandler, W.L., Yeung, W., and Tait, J.F. (2011). A new microparticle size calibration standard for use in measuring smaller microparticles using a new flow cytometer. J Thromb Haemost *9*, 1216–1224.
15. Maecker, H.T., and Trotter, J. (2006). Flow cytometry controls, instrument setup, and the determination of positivity. Cytometry *69*, 1037–1042.
16. Cook, S.M., Tang, V.A., Lannigan, J., Jones, J.C., and A, W.J. (2023). Quantitative Flow Cytometry (qFCM) protocols for end-to-end optimization of cross-platform extracellular vesicle studies. Protocols.io.
17. Lee, J.A., Spidlen, J., Boyce, K., Cai, J., Crosbie, N., Dalphin, M., Furlong, J., Gasparetto, M., Goldberg, M., Goralczyk, E.M., et al. (2008). MIFlowCyt: the minimum information about a Flow Cytometry Experiment. Cytometry *73*, 926–930.
18. Welsh, J.A., Horak, P., Wilkinson, J.S., Ford, V.J., Jones, J.C., Smith, D., Holloway, J.A., and Englyst, N.A. (2020). FCMPASS Software Aids

Extracellular Vesicle Light Scatter Standardization. Cytometry *97*, 569–581.

19. Welsh, J.A., and Jones, J.C. (2020). Small Particle Fluorescence and Light Scatter Calibration Using FCMPASS Software. Current Protocols in Cytometry *94*, e79.

20. Parks, D.R., Moore, W.A., Brinkman, R.R., Chen, Y., Condello, D., El Khettabi, F., Nolan, J.P., Perfetto, S.P., Redelman, D., Spidlen, J., et al. (2018). Methodology for evaluating and comparing flow cytometers: A multisite study of 23 instruments. Cytometry *93*, 1087–1091.

STAR★METHODS

KEY RESOURCES TABLE

REAGENT or RESOURCE	SOURCE	IDENTIFIER
Antibody		
anti-CD81 APC	Biolegend	Cat 349509, Lot B340367, RRID:AB_2564021
anti-CD81 Pacific Blue	Biolegend	Cat 349515, Lot B349914, RRID:AB_2687126
anti-CD81 PE	Biolegend	Cat 349505, Lot B330215, RRID:AB_10645519
Software and algorithms		
MATLAB	Mathworks	https://www.mathworks.com/
FCM_{PASS}	National Cancer Institute	https://nano.ccr.cancer.gov/fcmpass
Data analysis scripts	This paper	https://doi.org/10.6084/m9.figshare.22335835.v1
Other		
Aurora + ESP (V-B-Y-R), NIH	Cytek Biosciences	N/A
Aurora + ESP (UV-V-B-Y-R), Cytek	Cytek Biosciences	N/A
Aurora + ESP (UV-V-B-Y-R), uOttawa	Cytek Biosciences	N/A
Aurora + ESP (UV-V-B-Y-R), AFC	Cytek Biosciences	N/A
CytoFLEX (V-B-Y-R), NIH	Beckman Coulter	N/A
CytoFLEX (V-B-Y-R), AFC1	Beckman Coulter	N/A
CytoFLEX (V-B-Y-R), AFC2	Beckman Coulter	N/A
CytoFLEX (V-B-Y-R), AFC3	Beckman Coulter	N/A
DI water	Sigma Aldrich	Cat 270733
DPBS	Thermo Fisher Scientific	Cat. 270733
Exosome standards, fluorescent (rEVs)	Millipore Sigma	Cat. SAE0193, Lot 125377
FluoSpheres Carnboxylate Beads (100 nm)	Thermo Fisher Scientific	F8803
Low Protein Binding Tubes (1.5 mL)	Thermo Fisher Scientific	Cat. 90410
Low Protein Binding Tubes (0.5 mL)	Thermo Fisher Scientific	Cat. 88379
MESF Beads (APC)	Becton Dickinson	Cat. 626425, Lot 0273462
MESF Beads (BV421)	Becton Dickinson	Cat. 625508, Lot 026682
MESF Beads (FITC)	Bangs Laboratories	Cat. 555B, Lot 14610
MESF Beads (PE)	Becton Dickinson	Cat 340495, Lot 51753
NIST-traceable beads (81 nm)	Thermo Fisher Scientific	Cat. 3080A, Lot 228748
NIST-traceable beads (100 nm)	Thermo Fisher Scientific	Cat. 3100A, Lot. 204935
NIST-traceable beads (152 nm)	Thermo Fisher Scientific	Cat. 3150A, Lot. 202026
NIST-traceable beads (203 nm)	Thermo Fisher Scientific	Cat. 3200A, Lot. 205131
NIST-traceable beads (240 nm)	Thermo Fisher Scientific	Cat. 3240A, Lot. 226952
NIST-traceable beads (303 nm)	Thermo Fisher Scientific	Cat. 3300A, Lot. 204665
NIST-traceable beads (345 nm)	Thermo Fisher Scientific	Cat. 3350A, Lot. 199283
NIST-traceable beads (401 nm)	Thermo Fisher Scientific	Cat. 3400A, Lot. 203859
NIST-traceable beads (453 nm)	Thermo Fisher Scientific	Cat. 3450A, Lot. 204047
Protein LoBind Tubes (5 mL)	Eppendorf	Cat. 30122356
QbSure Beads	Cytek Biosciences	Cat B7-10005, Lot AF01
V-botton plates (96-well)	Evergreen	Cat. 222-8031-01V

RESOURCE AVAILABILITY

Lead contact

Requests for further information should be directed to the lead contact: Joshua Welsh, joadwe@outlook.com.

Materials availability

No unique reagents were generated in this study.

Data and code availability

(1) The code used to generate all figures and supplementary information in the manuscript can be found on FigShare: https://doi.org/10.6084/m9.figshare.22335835.v1.
(2) All data used in the paper can be found on the NanoFlow Repository at: https://genboree.org/nano-ui/manuscript/1753349353
(3) Any additional information required to reanalyze the data reported in this work paper is available from the lead contact upon request.

EXPERIMENTAL MODEL AND SUBJECT DETAILS

Ethics statement

No human samples were utilized in this study. All reagents and reference materials utilized in this study are commercially available with information shared in the key resource table.

METHOD DETAILS

Instrument characterization using SPOT pipeline

Instrument characterization is initially started by running the manufacturer's daily QC procedure to ensure correct laser delays for downstream analysis, and to ensure detectors are within their normal working ranges as determined by the manufacturer, Figure 1. From here, the light scatter detector optimization is performed utilizing Methods S1. This process identifies the optimal light scatter detector settings by performing detector setting incrementation (DSI) analysis using a fluorescent trigger. DSI is a method where the same reference material is acquired multiple times, with each acquisition having an increasing detector setting, such as voltage or gain. Once found, the optimal light scatter detector settings are then used to determine the optimal trigger threshold settings. The optimal trigger settings are found by using a buffer only control and reducing the threshold until a stable event rate of 1000–1500 events s^{-1} is found. In a clean system, the majority of triggered events using the buffer only control will be from the opto-electronic noise population which is critical to identify for downstream fluorescence limit of detection. This population is distinct from sheath and sample debris in that it has a sharp increase in event rate once reached and can be triggered on any detector. Optimal fluorescent detector settings and limit of detection are next found by performing DSI analysis using fluorescent multi-peak beads and using the light scatter trigger on the opto-electronic noise previously identified (Methods S2). Finally, the sensitivity of the light scatter detector is quantitated by acquiring NIST-traceable beads and performing light scatter calibration (Methods S3). Protocols used to validate the SPOT pipeline derived settings using recombinant extracellular vesicles (rEVs) can be found for light scatter detector settings (Methods S4), fluorescence detector settings and sensitivity (Methods S5), light scatter sensitivity (Methods S6), and antibody staining (Methods S7). All resources can be found in the supplemental information and online.[16]

Flow cytometry

FCM fluorescence and light scattering settings were optimized and calibrated utilizing FCM_{PASS} (v4.2, https://nano.ccr.cancer.gov/fcmpass). To demonstrate the consistency of optimal gain derivation on the CytoFLEX platform that showed less of a clear improvement than the Aurora platform, the optimal gain characteristics were derived fives and plotted, Figure 1. Calibration reference materials, acquisition files, settings, QC plots, MIFlowCyt and MIFlowCyt-EV reports can be found in https://genboree.org/nano-ui/manuscript/1753349353.[12,17–19] All Aurora (Cytek Bioscience) instruments had an enhanced small particle detection (ESP) module on the 405 nm laser. Three Aurora's were configured with 50 mW 355, 405, 488, 561, and 640 nm, One Aurora was configured with 50 mW 405 and 640 nm lasers, 150 mW 488 and 561 nm lasers, and a quantiFlash LED pulser (APE) and electronically integrated FSC LED pulser trigger.[20] All CytoFLEX S (Beckman Coulter) platforms were equipment with 405, 488, 561, 640 nm lasers. Flow cytometers were calibrated using QbSure beads cross-calibrated to molecules of equivalent soluble fluorophore (MESF) units using FCM_{PASS} on the NIH CytoFLEX and Aurora flow cytometers. The assigned bead values were used across all other cytometers of the same platform. Aurora and CytoFLEX cross-calibrations can be found in Figure 4.

Cross-calibration of ERF to MESF

Currently, commercially available fluorescence calibration reference materials are available in MESF and equivalent reference fluorophore (ERF) units. An understanding of the differences between MESF and ERF units is critical when attempting to make comparisons across platforms and assays. In the context of FCM applications, a detector scale calibrated to MESF units is quantifying a signal from a fluorophore whose spectroscopic properties match the fluorophore being quantified e.g., a detector's intensity scale is calibrated into molecules of phycoerythrin (PE) and the particles being phenotyped are labeled with a PE-conjugated antibody. A detector scale calibrated to ERF units is quantifying a signal whose spectroscopic properties do not match the fluorophore being quantified. Figure S5 demonstrates how ERF and MESF units relate and can be made interoperable by factoring in spectroscopic

data. Figure S5A shows a detector's intensity scale calibrated into molecules of fluorescein Isothiocyanate (FITC) and particles fluorescently tagged with enhanced green fluorescent protein (EGFP). When EGFP-tagged rEVs are detected between two platforms with different fluorescent detection filters, the intensity of the rEV population detected by the CytoFLEX is approximately 23% higher than the Aurora, with median FITC ERF intensities of 505 and 412. Currently, FITC reference materials are readily available, those for EGFP are not.

When observing the spectra of FITC and EGFP, Figure S15B, it is evident that the emission properties of the two fluorophores differ in their region of excitation maximum, with EGFP at ~508 nm and FITC at ~525 nm. Furthermore, the CytoFLEX S collection filter has a larger bandwidth (525/40) than the Aurora (525.5/17). The result of differing collection bandwidths is a difference in brightness collected with the CytoFLEX collecting ~2x more photons than the Aurora for FITC and ~2.5x more photons from EGFP. The difference in collection bandwidth not only affects the quantity of light collected but also the ratio of FITC molecules to GFP molecules, Figure S5C. One molecule of FITC on the CytoFLEX S is equivalent to ~0.57 molecules of EGFP, whereas on the Aurora one molecule of FITC is equivalent to ~0.47 molecules of EGFP, Figure S5D. By accounting for fluorophore brightness and collection filter bandwidths, it is possible to approximately convert FITC ERF to EGFP MESF by dividing the ERF values by 0.57 on the CytoFLEX S and 0.47 on the Aurora platforms, Figure S5E. This conversion to MESF allows for direct comparisons of fluorescent information between the two platforms with the 23% difference in FITC ERF units reduced to 1% difference in EGFP MESF units. The median rEV intensities now being 882 GFP MESF for the CytoFLEX and 875 GFP MESF for the Aurora.

The ability to make MESF comparisons is therefore not limited to the availability of fluorescence reference materials but can be approximated by accounting for differences in fluorophore spectroscopic properties using readily available fluorophore spectroscopic information and the collection filters used within a flow cytometer. GFP MESF throughout this work utilize this normalization method.

Reference material preparation

Commercial (rEVs) (Millipore Sigma, Cat. SAE0193) were resuspended from lyophilization according to manufacturer recommendations in 100 μL deionized water (Sigma Aldrich, Cat. 270733) and reverse pipetted. Serial dilutions of resuspended rEVs in DPBS, Figure S6, were used to confirm the concentration of ~5 x10^6 particles mL^{-1} resulted in single particle detection that was used for all downstream analyses.

rEV immunophenotyping

Three identical clones (TAPA-1) of CD81 antibody conjugated to three different fluorophores were tested: pacific blue, phycoerythrin (PE), and allophycocyanin (APC). Antibodies were incubated from 6.25x10^{-2} to 2 μg mL^{-1} and compared at 30 min and overnight incubations (~12 h). Buffer + antibody controls, unstained rEVs, and antibody-labelled rEVs were acquired. A stain index, calculated using qFCM EGFP-positive rEVs data incorporating the buffer with antibody control in lieu of a negative rEV population, was used to compared labeling efficacy while accounting for the fluorescence contribution from unbound antibodies, along with ERF and MESF unit calibrations, Figures S2 and S3.

QUANTIFICATION AND STATISTICAL ANALYSIS

Statistical analysis

Data analyses were performed in MATLAB (Mathworks Inc, v2022b). All manuscript data and MATLAB data analysis and figure generation scripts can be accessed at the repository link: https://doi.org/10.6084/m9.figshare.22335835.v1. Unless otherwise stated all stated fluorescent intensities are based on the median statistic due to the non-parametric nature of the fluorescent intensity. Unless otherwise stated the lower LoD was obtained using the 99th percentile of the background noise (DPBS) population. Normality testing of noise population utilized a MATLAB's default Kruskal-Wallis, skewness and kurtosis functions. Stain indices were calculated using the following formula:

$$\frac{(rEV\ median - buffer{+}antibody\ median)}{95^{th} percentile\ of\ buffer{+}antibody}.$$

Cell Reports Methods, Volume *3*

Supplemental information

Quantitative flow cytometry enables end-to-end optimization of cross-platform extracellular vesicle studies

Sean Cook, Vera A. Tang, Joanne Lannigan, Jennifer C. Jones, and Joshua A. Welsh

Supporting Information for

Quantitative flow cytometry (qFCM) enables end-to-end optimization of cross-platform extracellular vesicle studies.

Sean Cook, Vera A. Tang, Joanne Lannigan, Jennifer C. Jones, Joshua A. Welsh

Lead author: Joshua A. Welsh.
E-mail: joadwe@outlook.com

This PDF file includes:

Figs. S1 to S6
Table S1

Contents

1 Flow cytometer noise characterization methods **3**

2 CytoFLEX light scatter optimal gain derivation laser power and rEV detection **4**

3 Recombinant EV immunophentyping **5**

4 Rainbow bead cross-calibration **7**

5 FITC ERF to GFP MESF **8**

6 Recombinant EV serial dilution **9**

7 Supplementary Methods **10**

A Methods S1: Flow cytometer light Scatter detector setting incrementation, related to Figure 1 10
B Methods S2: Flow cytometer fluorescence detector setting incrementation, related to Figure 1 18
C Methods S3: Flow cytometer light scatter calibration, related to Figure 1 26
D Methods S4: Flow cytometer rEV serial dilution, related to Figure 1 34
E Methods S5: Flow cytometer rEV light scatter detector setting incrementation, related to Figure 1 43
F Methods S6: Flow cytometer rEV fluorescence detector setting incrementation, related to Figure 1 51
G Methods S7: Flow cytometer rEV immunophenotyping, related to Figure 1 58

1. Flow cytometer noise characterization methods

Name	Lower LoD (FITC ERF)	Normality (p-value)	Skewness	Kurtosis
QbSure	421	<0.001	0.1036	2.8962
LED Pulser	212	<0.001	0.1578	3.2118
100 nm	58	<0.001	0.4456	3.6074
81 nm	47	<0.001	0.4292	3.5076
DPBS	27	<0.001	0.6572	4.2718

Table S1. Comparison of reference materials available for assessing fluorescence lower limits of detection, derived from Figure 3B.

2. CytoFLEX light scatter optimal gain derivation laser power and rEV detection

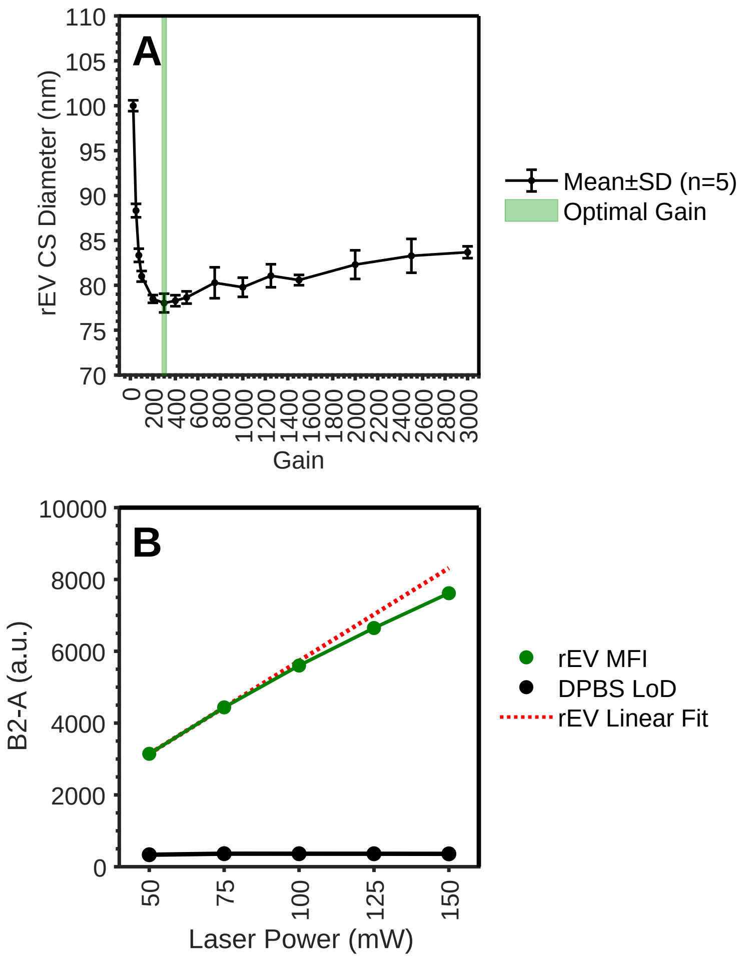

Fig. S1. Stability of SPOT pipeline, as outlined in Figure 3. A) CytoFLEX LX light scatter detector setting incrementation showing the mean and standard deviation of the derived limit of detection from the SPOT pipeline. Optimal gain derived by the SPOT pipeline is highlighted in green. B) Median fluorescent intensity (MFI) of recombinant EVs (green) and 95th percentile of DPBS, which is the limit of detection (LoD).

3. Recombinant EV immunophentyping

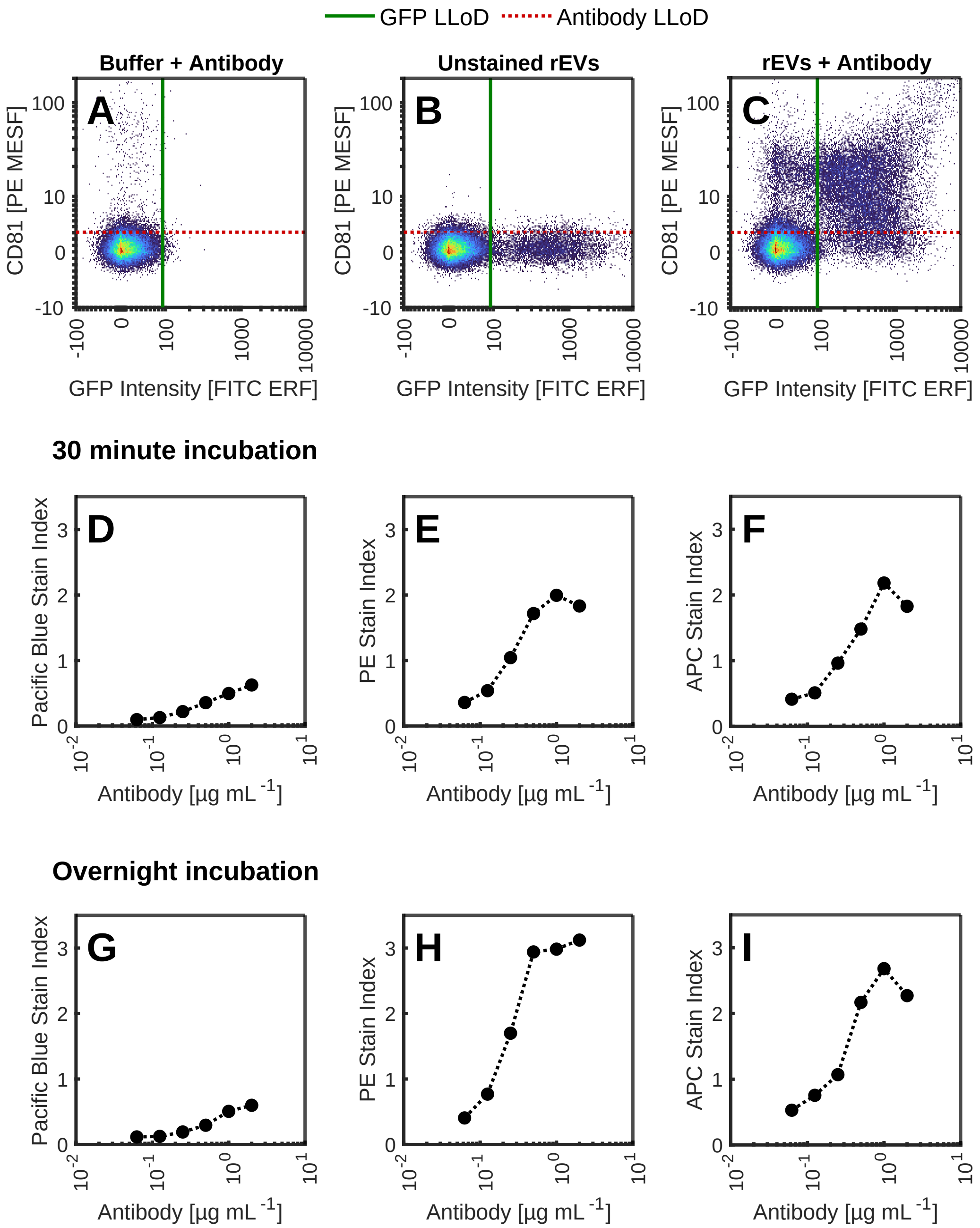

Fig. S2. Ability to phenotype at optimized settings, as derived from Figure 3. Optimization of antibody staining with stain index. rEV were stained with anti-CD81-PE antibody with buffer + antibody control (A), unstained rEV population (B), and 2 µg mL-1 anti-CD81-PE-stained rEV population incubated overnight at 2 µg mL-1 (C), before a 200-fold dilution for acquisition. Stain index of stained rEVs with anti-CD81-Pacific Blue (D, G), PE (E, H), and APC (F, I) at 30 minutes and overnight, respectively.

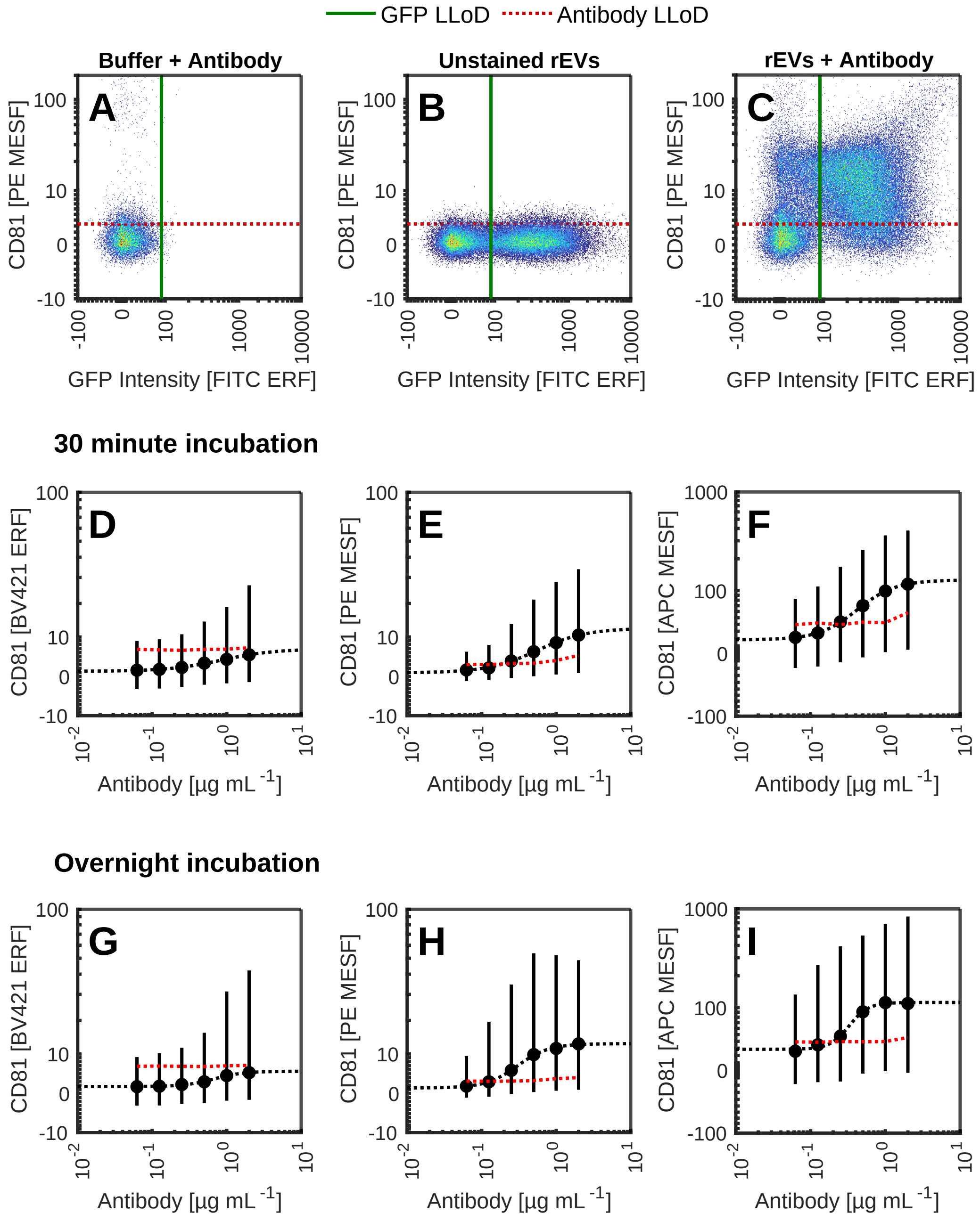

Fig. S3. Ability to phenotype at optimized settings, as derived from Figure 3. Optimization of antibody staining with calibrated units. Acquisition of buffer with antibody incubated overnight at 2 µg mL-1 anti-CD81-PE before a 200-fold dilution for acquisition (A), unstained rEV population (B), and 2 µg mL-1 anti-CD81-PE-stained rEV population incubated overnight (C). Calibrated intensity of stained rEVs with anti-CD81-Pacific Blue (D, G), PE (E, H), and APC (F, I) at 30 minutes and overnight, respectively. The limit of detection (95th percentile) using the buffer with antibody control is denoted by the dotted red line.

4. Rainbow bead cross-calibration

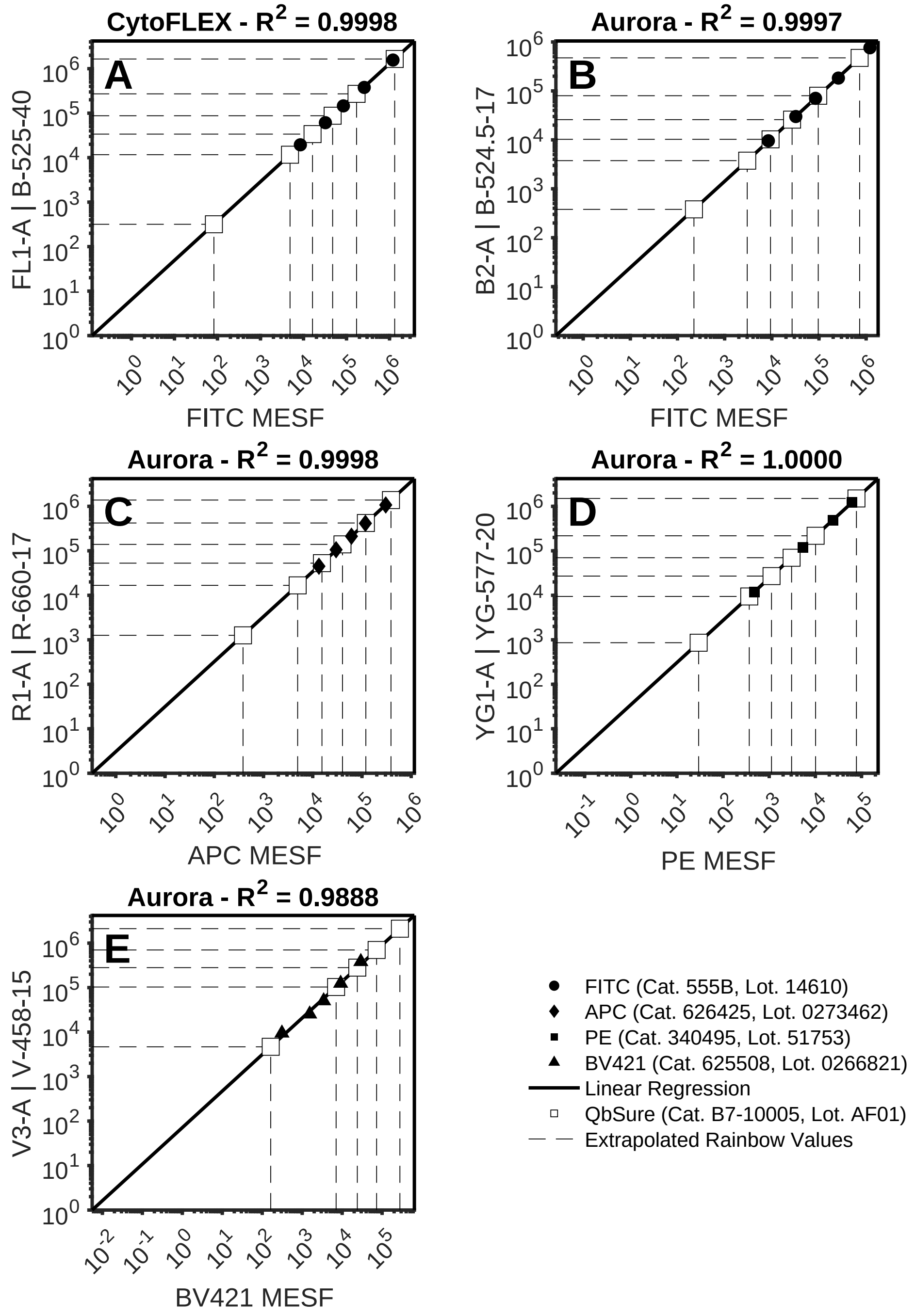

Fig. S4. Cross-calibration of QbSure beads to ERF units using MESF beads on the CytoFLEX and Aurora flow cytometers, as used in Figures 2-7

5. FITC ERF to GFP MESF

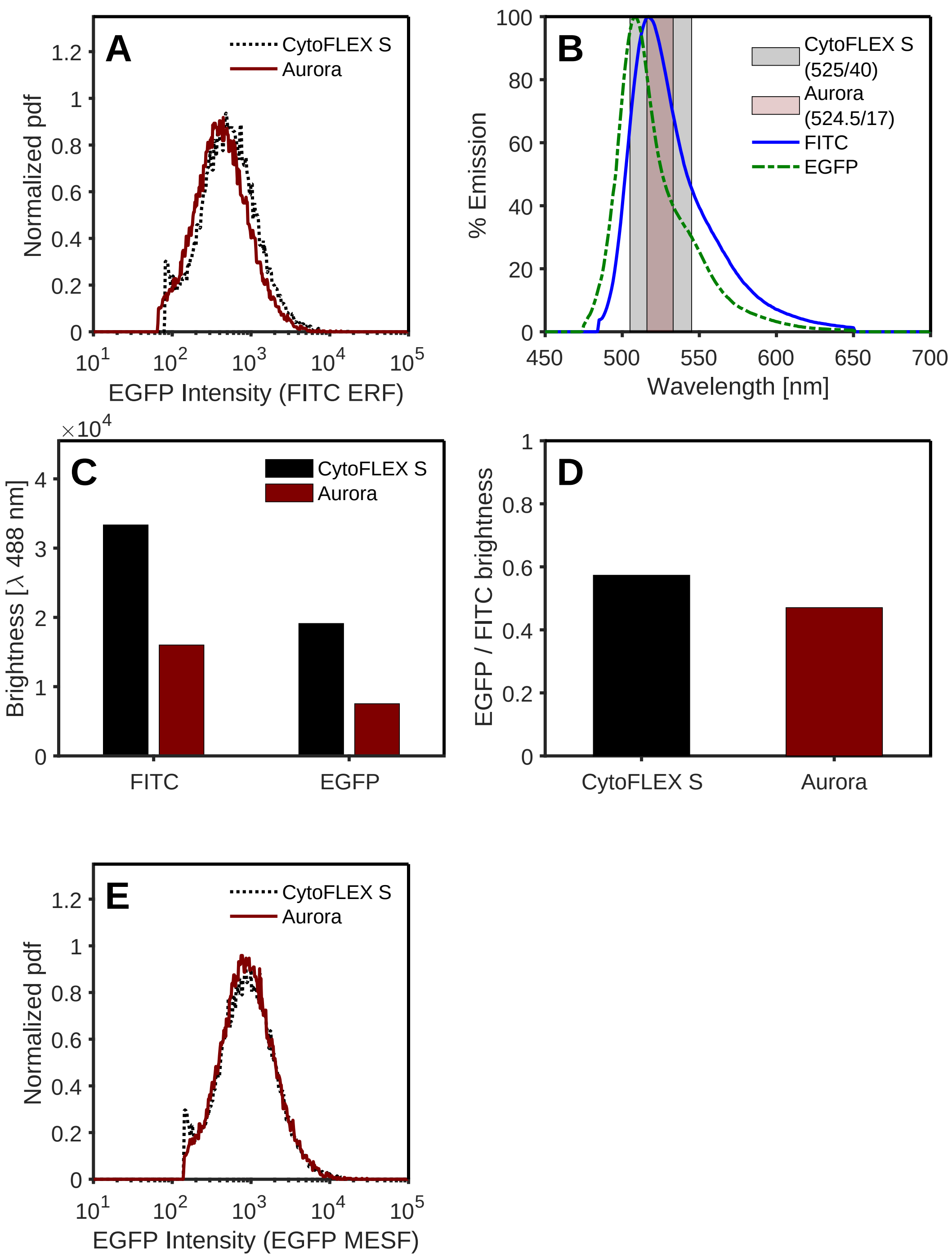

Fig. S5. Utilization of cross-calibration, as used in Figures 2-7. A) rEV population calibrated to FITC ERF units on the CytoFLEX S (dashed blacked line) and Aurora (solid red line) platforms. B) Normalized emission spectra for fluorescein isothiocyanate (FITC) (solid blue line) and enhanced green fluorescent protein (EGFP) (dashed dotted green line). The collection bandwidths for the CytoFLEX S (grey) and Aurora (red) as overlaid on the emission spectra. C) Collected light from FITC and EGFP fluorophores on the CytoFLEX S (black) with a bandpass of 525/40 nm and the Aurora (red) with a bandpass of 524.5/17 nm. Brightness of FITC assumes extinction coefficient of 75000 M-1 cm-1, quantum yield of 0.92, and excitation of 77.33% at 488 nm. Brightness of EGFP assumes extinction coefficient of 55900M-1 cm-1, quantum yield of 0.6, and excitation of 99.87% at 488 nm. D) Ratio of EGFP to FITC brightness between CytoFLEX (red) and Aurora (black) account for relative brightness and collection bandwidth. E) rEV population calibrated to EGFP MESF units on the CytoFLEX S (dashed blacked line) and Aurora (solid red line).

6. Recombinant EV serial dilution

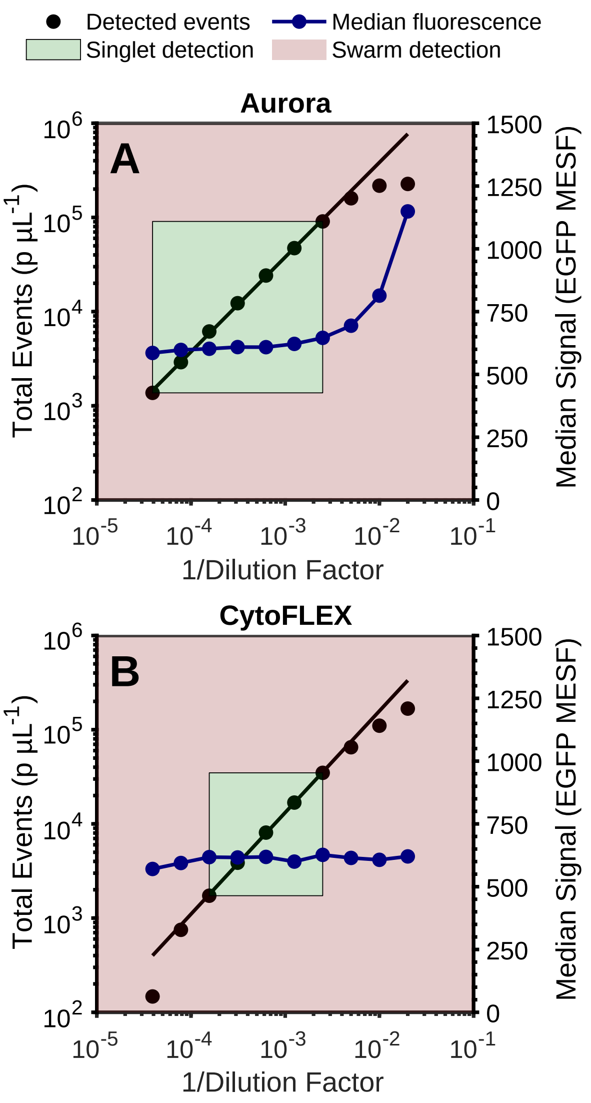

Fig. S6. rEV serial dilution on Cytek Aurora (A) and Beckman Coulter CytoFLEX S (B), as used in Figures 2, 4-7. Representative serial dilution of rEV stock on Aurora platform. Measured (black dots) and robust regression (solid black line) concentration of rEVs plotted on left y axis. Measured median fluorescent intensity in calibrated units shown on right y axis. (blue dots). Green region represents reliable single particle detection where there is a linear decrease in particle number and stable median fluorescent intensity. Red region represents coincidence detection.

7. Supplementary Methods

A. Methods S1: Flow cytometer light Scatter detector setting incrementation, related to Figure 1.

Resource 1: Scatter Detector Setting Incrementation for FCMPASS

Forked from Flow Cytometer Fluorescence Voltration for FCMPASS

In 1 collection

Sean M Cook[1], Jennifer Jones[1], Joshua A Welsh[1]

[1]Translataional Nanobiology Section, Laboratory of Pathology, Center for Cancer Research, National Cancer Institute, National Institutes of Health

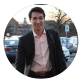

Sean M Cook

Protocol Info: Sean M Cook, Jennifer Jones, Joshua A Welsh . Resource 1: Scatter Detector Setting Incrementation for FCMPASS. **protocols.io** https://protocols.io/view/resource-1-scatter-detector-setting-incrementation-cnjcvciw

Created: Jan 30, 2023

Last Modified: Mar 25, 2023

PROTOCOL integer ID: 76100

DISCLAIMER

This protocol summarizes key steps for a specific type of method, which is one of a collection of methods and assays used for EV analysis in the NCI Translational Nanobiology Section at the time of submission of this protocol. Appropriate use of this protocol requires careful, cohesive integration with other methods for EV production, isolation, and characterization.

ABSTRACT

Protocol to perform flow cytometer voltration to identify optimal detector settings for small particle analysis. Data acquired from this protocol are compatible with semi-automated analysis tools built into FCMPASS software.

MATERIALS

FluoSpheres Carnboxylate **Thermo Fisher Scientific Catalog #F8803** , referred to as 'FluoSpheres'

5 mL Round-bottom tube **Corning Catalog #352052** , referred to as 'FACS tubes'

DPBS **Thermo Fisher Scientific Catalog #14190144** , referred to as 'DPBS'

QbSure **Cytek Catalog #B7-10005** , referred to as 'QbSure'

Sample preparation

10s

1 Vortex FluoSpheres bottle on a high setting for 00:00:05 . 5s

Note

This stock bottle is 2% solids containing 110 nm beads, equal to ~2.73E13 / mL

Note

This method in principle is compatible with any fluorescent nanosphere that is bright enough to be fully resolved when using a fluorescent trigger.

2 Create 3 mL of 5E6 p/mL solution of FluoSpheres in a FACS tube.

Note

An observation from our protocol development is that using low protein binding tubes for this step may negatively impact the efficacy of this protocol as it can result in excess unbound fluorophore from the beads increasing background noise leading to excessive event rate.

2.1 Pipette 2998.90 µL of DPBS into a FACS tube. Label this tube '1E10 Intermediate'.

2.2 Pipette 1.10 µL stock FluoSpheres into the tube labelled '1E10 Intermediate' . Reverse pipette the tube to mix.

2.3 Pipette 990 µL of DPBS into a FACS tube. Label this tube '1E8 Intermediate'. Pipette 10 µL "1E10 Intermediate" into the tube. Reverse pipette the tube to mix.

2.4 Pipette 2850 µL of DPBS into a FACS tube. Label this tube 'Beads. Pipette 150 µL "1E8 Intermediate" into the tube. Reverse pipette the tube to mix. This should result in a concentration of ~5E6 p/mL of FluoSpheres, and this tube will be used for acquisition.

3 Pipette 500 µL of DPBS into a FACS tube. Label this tube 'DPBS'.

Cytometer Setup

4 Ensure cytometer is clean and that -Height and -Area statistics are set to be collected on all parameters and that all parameters are on.

5 On the Cytek Aurora, set window extension to 0. On the CytoFLEX platform turn on 'High Acquisition Mode'.

Note

To set the window extension to 0 on the Cytek Aurora, navigate to the 'Lasers' tab under the sample acquisition settings and set the window extension to 0.

To change the acquisition mode on the CytoFLEX S, click on the 'Advanced' menu on the top-of-the-screen ribbon. Next click on 'Event Rate Settings' menu, change the acquisition mode to 'High', and press 'Ok'.

6 Create a histogram plot with the FITC height parameter (CytoFLEX: FL1-H | B-525-40, Aurora: B2 | B-524.5-17) on the X-Axis and make sure it is plotted on a log-scale.

7 Create a histogram plot with (405 nm) violet SSC-A (CytoFLEX: SSC_1-A, Aurora: SSC-A) on the X-Axis and make sure it is plotted on a log-scale.

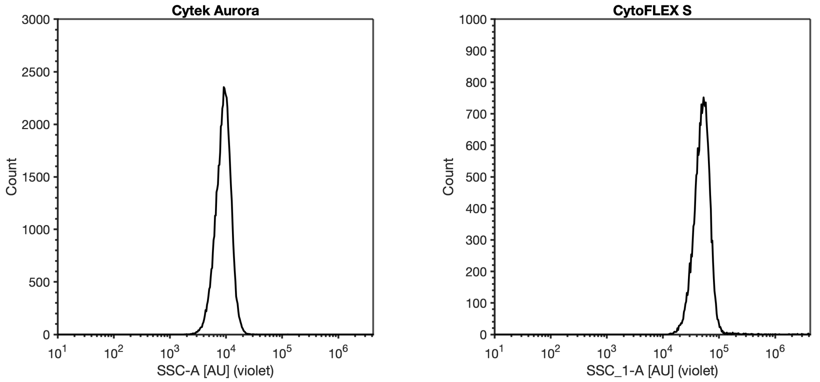

Example of FluoSpheres acquired on Cytek Aurora and CytoFLEX S. Data has been gated to remove the noise population.

8 Set the cytometer triggering threshold to the FITC parameter (CytoFLEX: FL1 | B-525-40, Aurora: B2 | B-524.5-17) . **All samples should be acquired with the lowest flow rate, typically**

~10-15 µL min^{-1}.

Note

Cytometer Voltage/Gain and threshold settings are subjective due to their dependency on alignment, and the scatter filters in place, amongst other variables. The following are guide values to start with and may need adjustment for optimal acquisition.

Beckman Coulter, CytoFLEX [405 nm OD0 filter, 488 nm OD2 filter]

- Threshold FL1 | B-525-40-H = 550;
- FL1 | B-525-40 Gain = 500

Cytek Bioscience, Aurora [405 nm OD0 filter, 488 nm OD2 filter]

- Threshold B2 | B-524.5-17-H = 500;
- B2 | B-524.5-17 Gain = 1750

9 Acquire the 'DPBS' tube while viewing the FITC histogram plot from go to step #6. Adjust the detector gain or trigger threshold until the instrument noise is being **acquired at ~1000 events/sec.**

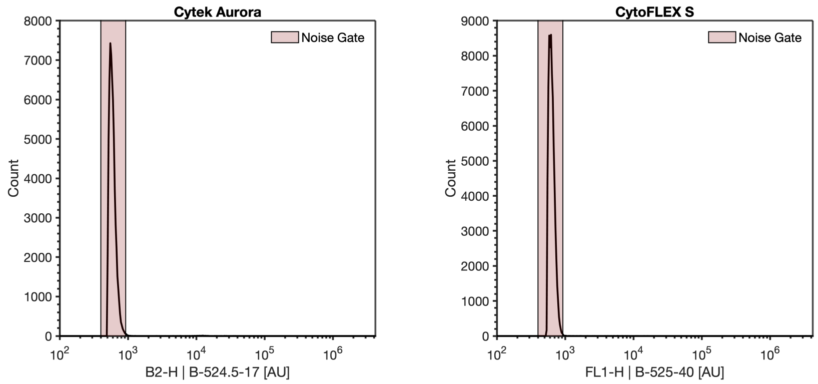

Acquisition of DPBS on the Cytek Aurora and CytoFLEX S, with the Aurora triggering on instrument noise on the B2-H channel at gain 1750 and threshold 500 and the CytoFLEX triggering on instrument noise on the FL1-H channel at gain 500 and threshold 550.

Note

The instrument noise floor is distinct from detected background events in sheath as it has a sharp increase. In a system with debris there may be a tail that elongates out of this this sharp peak.

9.1 Recording this noise is not necessary as this step is identifying optimal settings.

10 Acquire the "Beads" tube from go to step #2 . Using the plot from go to step #6 , ensure the FluoSpheres are visible on the FITC and violet SSC parameter. Use the FITC trigger settings identified in go to step #9

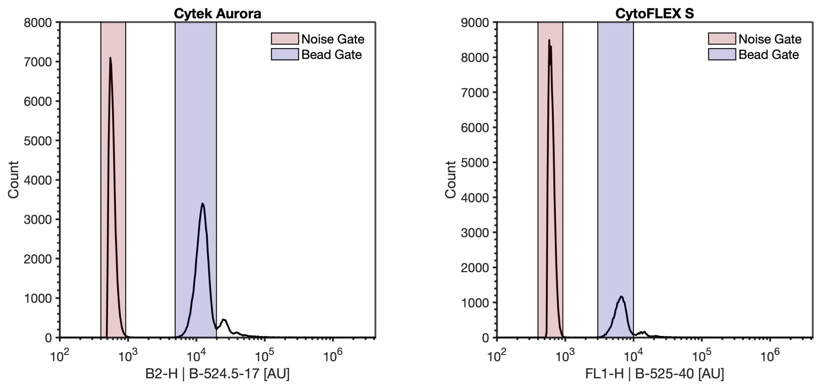

Acquisition of 110 nm FluoSpheres in DPBS on the Cytek Aurora and CytoFLEX S, with the Aurora triggering on instrument noise on the B2-H channel at gain 1750 and threshold 500 and the CytoFLEX triggering on instrument noise on the FL1-H channel at gain 500 and threshold 550.

Note

The total event rate when acquiring the FluoSpheres should not exceed 6000 events/sec. If the event rate is higher than 6000 events/sec, further dilution of the FluoSpheres is necessary prior to acquisition.

11 Draw a gate around the FluoSpheres on the FITC parameter. Label this gate 'Bead Gate'

12 Adjust the stopping criteria of the instrument to record until at least 2,000 events are acquired on 'Bead Gate' drawn in go to step #11 .

Performing Voltration

13 Voltration can now be performed by recording the 'Beads' tube at multiple light scatter detector gains, leaving the trigger threshold and fluorescent gains consistent. It is recommended that a recording of at least 10 light scatter detector settings is taken. Including more increments within a voltration will result in being more confident of the subsequent optimal detector settings.

Acquisition Template.xlsx

Note

For flow cytometers with avalanche photodiodes its is recommended that the detector settings have more incrementation at lower gains than higher gains while for instruments with photomultiplier tubes they should be spaced evenly. See template for example of settings for each tube analyses

Example Gain Voltration for CytoFLEX & Aurora

1. 50
2. 100
3. 200
4. 300
5. 400
6. 500
7. 750
8. 1000
9. 1250
10. 1500
11. 2000
12. 2500
13. 3000

13.1 To ensure accurate data analysis, the FluoSpheres must separate from the noise population on the light scatter parameter on at least two of the selected gains. If they do not, repeat this protocol using a 200 nm FluoSphere population.

B. Methods S2: Flow cytometer fluorescence detector setting incrementation, related to Figure 1.

Resource 2: Fluorescence Detector Setting Incrementation for FCMPASS

Forked from Flow Cytometer Fluorescence Voltration for FCMPASS

In 1 collection

Joshua A Welsh[1], Sean M Cook[1], Jennifer Jones[1]

[1]Translataional Nanobiology Section, Laboratory of Pathology, Center for Cancer Research, National Cancer Institute, National Institutes of Health

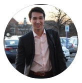

Sean M Cook

Protocol Info: Joshua A Welsh, Sean M Cook, Jennifer Jones . Resource 2: Fluorescence Detector Setting Incrementation for FCMPASS. **protocols.io** https://protocols.io/view/resource-2-fluorescence-detector-setting-increment-cnjjvckn

Created: Jan 30, 2023

Last Modified: Mar 15, 2023

PROTOCOL integer ID: 76107

DISCLAIMER

This protocol summarizes key steps for a specific type of method, which is one of a collection of methods and assays used for EV analysis in the NCI Translational Nanobiology Section at the time of submission of this protocol. Appropriate use of this protocol requires careful, cohesive integration with other methods for EV production, isolation, and characterization.

ABSTRACT

Protocol to perform flow cytometer voltration to identify optimal detector settings for small particle analysis. Data acquired from this protocol are compatible with semi-automated analysis tools built into FCMPASS software.

MATERIALS

- QbSure **Cytek Catalog #B7-10005** , referred to as 'QbSure'
- DPBS **Thermo Fisher Scientific Catalog #14190144** , referred to as 'DPBS'
- 5 mL Round-bottom tube **Corning Catalog #352052** , referred to as 'FACS tube'
- FITC-5 MESF Beads **Bangs Laboratories Catalog #555B** , referred to as 'FITC MESF beads'

Sample preparation

10s

1 Vortex QbSure bottle on a high setting for 00:00:05 .

5s

Note

This protocol in principle is compatible with all multi-peak rainbow beads. Validation has only been tested on QbSure and Spheretech 8-peak beads. QbSure beads have been highlighted due to having consistently cleaner negative populations in our testing.

2 Pipette 500 µL of DPBS to two FACS tubes. Label one tube 'DPBS" and the second tube 'Beads'.

Note

An observation from our protocol development is that it is important not to use a low protein binding tube for this step, as it can result in excess unbound fluor from the beads creating background noise increases leading to excessive event rate.

3 Add 3 drops of QbSure beads to the 'Beads' tube and vortex for 00:00:05 . 5s

Cytometer Setup

4 Ensure cytometer is clean and that -Height and -Area statistics are set to be collected on all parameters and that all parameters are on.

5 On the Cytek Aurora, set window extension to 0. On the CytoFLEX platform turn on 'High Acquisition Mode'.

Note

To set the window extension to 0 on the Cytek Aurora, navigate to the 'Lasers' tab under the sample acquisition settings and set the window extension to 0.

To change the acquisition mode on the CytoFLEX S, click on the 'Advanced' menu on the top-of-the-screen ribbon. Next click on 'Event Rate Settings' menu, change the acquisition mode to 'High', and press 'Ok'.

6 Create a pseudocolor plot with FSC-H on the X-Axis and (488 nm) B-SSC-H on the Y-Axis and

make sure both parameters are being plotted on a linear-scale.

7 Create a histogram plot with (405 nm) V-SSC-H on the X-Axis and make sure it is plotted on a log-scale.

8 Set the cytometer triggering threshold to (405 nm) V-SSC-H. **All samples should be acquired with the lowest flow rate, typically ~10-15 µL min^{-1}.**

Note

Cytometer Voltage/Gain and threshold settings are subjective due to their dependency on alignment, and the scatter filters in place, amongst other variables. The following are guide values to start with and may need adjustment for optimal acquisition.

Beckman Coulter, CytoFLEX [405 nm OD0 filter, 488 nm OD2 filter]
- Threshold V-SSC-H = 1000;
- V-SSC Gain = 200
- FSC Gain = 100
- B-SSC Gain = 100

Cytek Bioscience, Aurora [405 nm OD0 filter, 488 nm OD2 filter]
- Threshold V-SSC-H = 1000;
- V-SSC Gain = 2500
- FSC Gain = 150
- B-SSC Gain = 40

9 Acquire the DPBS tube while viewing the histogram plot from go to step #7. Adjust the detector gain or trigger threshold until the instrument noise is being **acquired at ~1000 events/sec.** The instrument noise floor is distinct from detected background events in sheath as it has a sharp increase. In a system with debris there may be a tail that elongates out of this this sharp peak.

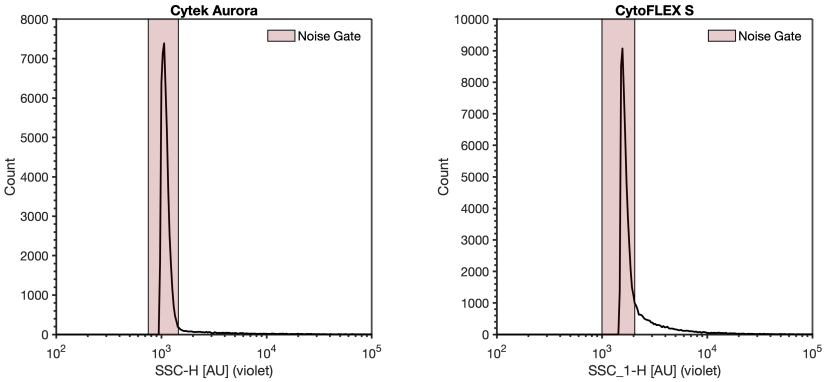

Example of threshold on the instrument noise floor on the Cytek Aurora and CytoFLEX S. Cytek Aurora triggered on SSC-H channel at gain 2500 and threshold 975. CytoFLEX S triggered on SSC_1-H channel at gain 250 and threshold 1200.

9.1 Recording this noise is not necessary as this step is identifying optimal settings.

10 Acquire the 'Beads' tube from go to step #3 . Using the plot from go to step #6 adjust the FSC and B-SSC gain until the single bead population is clearly visible and can be easily gated from the doublet population to the top right of it. Use the Violet SSC trigger settings identified in go to step #9

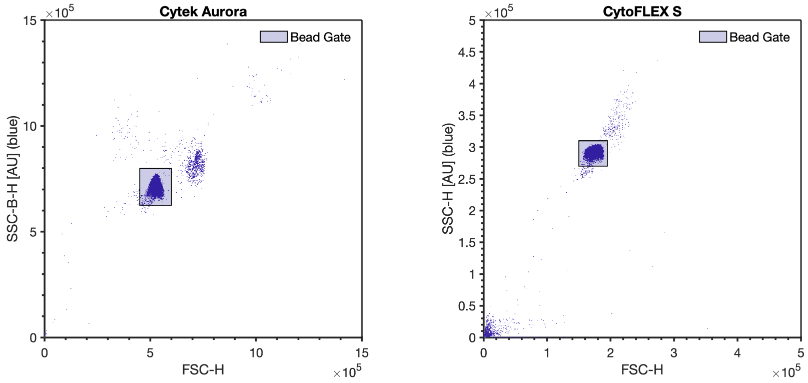

Example of clearly resolved singlet QbSure bead population on the Cytek Aurora and CytoFLEX S. Cytek Aurora FSC and SSC-B gains set to 150 and 40 respectively. CytoFLEX S FSC and SSC gains both set to 100.

11 Creating a gate around the single bead population named 'Bead Gate';

12 Adjust the stopping criteria of the instrument to record until 10,000 events are acquired on 'Bead Gate' drawn in go to step #11 .

Performing Voltration

13 Voltration can now be performed by recording the 'Beads' tube at multiple fluorescent detector gains, leaving the trigger threshold and light scatter gains consistent. It is recommended that a recording of at least 10 fluorescent detector settings is taken. Including more increments within a voltration will result in being more confident of the subsequent optimal detector settings.

Acquisition Template.xlsx

Note

For flow cytometers with avalanche photodiodes its is recommended that the fluorescent detector settings have more incrementation at lower gains than higher gains while for instruments with photomultiplier tubes they should be spaced evenly. See template for example of settings for each tube analyses

Example Gain Voltration for CytoFLEX & Aurora

1. 100
2. 200
3. 300
4. 400
5. 500
6. 750
7. 1000
8. 1250
9. 1500
10. 2000
11. 2500
12. 3000

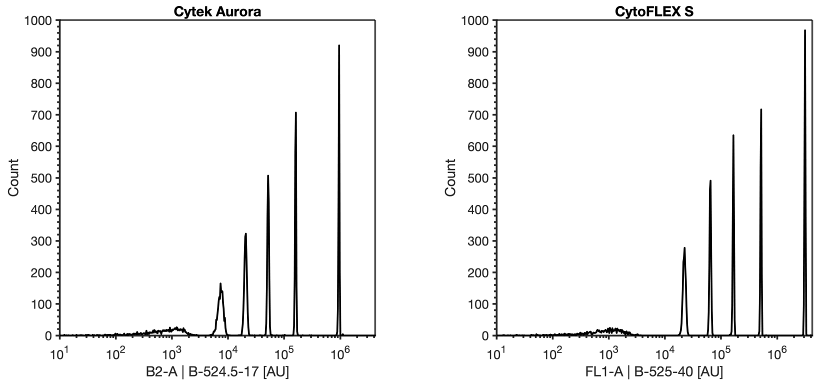

Example of QbSure beads acquired on Cytek Aurora and CytoFLEX S on the FITC parameter. Data has been gated to remove the noise population.

13.1 To ensure accurate data analysis, the brightest bead must be visible on at least two of the selected gains.

14 Fluorescent channels can be cross calibrated to determine lower limit of detection for the channel in calibrated units in the FCM_{PASS} software. A cross calibration between the desired MESF bead and QbSure beads should be acquired at a fluorescent detector gain where all MESF bead populations are **on-scale and fully resolved** from the noise.

Note

Cross calibration on Cytek Aurora and CytoFLEX were run with FITC MESF beads on the B2 | B-524.5-17 and FL1 | B-525-40 channels respectively. The QbSure beads were acquired at the same settings as the FITC MESF beads.

C. Methods S3: Flow cytometer light scatter calibration, related to Figure 1.

Resource 3: SSC Collection Optics and Calibration

Forked from FCMPASS - Acquisition and gating of light scatter reference materials

In 1 collection

Joshua A Welsh[1], Jennifer Jones[1]

[1]Translational Nanobiology Section, Laboratory of Pathology, Center for Cancer Research, National Cancer Institute, National Institutes of Health

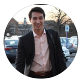

Sean M Cook

Protocol Info: Joshua A Welsh, Jennifer Jones . Resource 3: SSC Collection Optics and Calibration. **protocols.io** https://protocols.io/view/resource-3-ssc-collection-optics-and-calibration-cnmcvc2w

MANUSCRIPT CITATION:
Welsh J A, Jones J C,Small Particle Fluorescence and Light Scatter Calibration Using FCMPASSSoftware,Current Protocols in Cytometry, 94, e79. doi: 10.1002/cpcy.79

Created: Jan 31, 2023

Last Modified: Mar 25, 2023

PROTOCOL integer ID: 76164

Keywords: flow cytometry, calibration, extracellular vesicles

DISCLAIMER

This protocol summarizes key steps for a specific type of method, which is one of a collection of methods and assays used for EV analysis in the NCI Translational Nanobiology Section at the time of submission of this protocol. Appropriate use of this protocol requires careful, cohesive integration with other methods for EV production, isolation, and characterization.

Terms & Conditions of use for FCMPASS software.

Definitions: The term “SOFTWARE” throughout this agreement means the machine readable, binary, object code form, and the related documentation for FCMPASS, a software package that is designed to allow flow cytometer calibration for small particles. The term “RECIPIENT” means the party that downloads the software. The term “PROVIDER” means the National Cancer Institute (NCI), a participating institute of the National Institutes of Health (NIH), and an agency of the United States Government.By downloading or otherwise receiving the SOFTWARE, RECIPIENT may use the SOFTWARE subject to RECIPIENT’s agreement to the following terms:

1. THE SOFTWARE SHALL NOT BE USED IN THE TREATMENT OR DIAGNOSIS OF HUMAN SUBJECTS. RECIPIENT is responsible for compliance with all laws and regulations applicable to the use of the SOFTWARE.
2. RECIPIENT shall not distribute the SOFTWARE, in whole or in part without express advance written approval of PROVIDER.
3. The SOFTWARE may be used for research, academic, and educational purposes only. The SOFTWARE may not be used for commercial purposes. RECIPIENT will not license or sell or use the SOFTWARE for commercial purposes or applications.
4. The SOFTWARE that is distributed pursuant to this Agreement has been created by United States Government employees. In accordance with Title 17 of the United States Code, section 105, the SOFTWARE isnot subject to copyright protection in the United States. Other than copyright, all rights,

title and interest in the SOFTWARE shall remain with the PROVIDER.

5. RECIPIENT shall not modify, extend, decompile, make derivatives of, merge, publish, reverse engineer or distribute the SOFTWAREwithout written permission from PROVIDER.
6. RECIPIENT may publish or otherwise publicly disclose the results of using the SOFTWARE. RECIPIENT agrees to acknowledge PROVIDER’s contribution of the SOFTWARE in all written publications containing any data or information regarding or resulting from use of the SOFTWARE.
7. THE SOFTWARE IS PROVIDED "AS IS" AND ANY EXPRESS OR IMPLIED WARRANTIES, INCLUDING, BUT NOT LIMITED TO, THE IMPLIED WARRANTIES OF MERCHANTABILITY, FITNESS FOR A PARTICULAR PURPOSE AND NONINFRINGEMENT ARE DISCLAIMED. IN NO EVENT SHALL THE PROVIDER OR THE INDIVIDUAL DEVELOPERS BE LIABLE FOR ANY DIRECT, INDIRECT, INCIDENTAL, SPECIAL, EXEMPLARY, OR CONSEQUENTIAL DAMAGES (INCLUDING, BUT NOT LIMITED TO, PROCUREMENT OF SUBSTITUTE GOODS OR SERVICES; LOSS OF USE, DATA, OR PROFITS; OR BUSINESS INTERRUPTION) HOWEVER CAUSED AND ON ANY THEORY OF LIABILITY, WHETHER IN CONTRACT, STRICT LIABILITY, OR TORT (INCLUDING NEGLIGENCE OR OTHERWISE) ARISING IN ANY WAY OUT OF THE USE OF THIS SOFTWARE, EVEN IF ADVISED OF THE POSSIBILITY OF SUCH DAMAGE. PROVIDER makes no representations that the use of SOFTWARE will not infringe any patent or proprietary rights of third parties.
8. RECIPIENT may, on an as-needed basis, send to PROVIDER reports regarding the application of the SOFTWARE and the effectiveness and problems encountered in using the SOFTWARE, without disclosing RECIPIENT’s confidential information. Information from general reports may be used by the PROVIDER to enhance the capabilities of the SOFTWARE. Reports can be forwarded to the PROVIDER at one of the following addresses: joshua.welsh@nih.gov or jennifer.jones2@nih.gov
9. No indemnification for any loss, claim, damage, or liability is intended or provided by either Party under this Agreement. Each Party shall be liable for any loss, claim, damage, or liability that said Party incurs as a result of said Party's activities under this Agreement, except that Provider, as an agency of the United States, assumes liability only to the extent as provided under the Federal Tort Claims Act (28 U.S.C. Chapter 171 Sections 2671-2680).
10. RECIPIENT agrees not to claim, infer, or imply endorsement by the United States Government, or any of its organizational units, contractors or employees. RECIPIENT agrees not to use any trademarks, service marks, trade names, logos or product names of NCI or NIH to endorse or promote products derived from the SOFTWARE without specific, prior and written permission.
11. Title in the SOFTWARE shall remain with the PROVIDER. It is understood that nothing herein will be deemed to constitute, by implication or otherwise, the grant to either party by the other of any license or other rights under any patent, patent application or other intellectual property

right or interest. PROVIDER reserves the right to distribute the SOFTWARE to others and to use it for PROVIDER's own purposes. The United States Government explicitly retains all rights to use the SOFTWARE for any purpose, to have it used on the Government's behalf or to allow others to use it.

ABSTRACT

This protocol outlines the steps required to collect data from light scatter reference materials for use with the FCMPASS software. This is one of a number of protocols in the pipeline for performing small particle calibration using the fcmpass software package.

MATERIALS

MATERIALS

- Flow Cytometer **Contributed by users**
- Vortex **Contributed by users**
- Low Protein Binding Collection Tubes (1.5 mL) **Thermo Fisher Scientific Catalog #90410**
- DPBS **Invitrogen - Thermo Fisher Catalog #14190**
- NIST-Traceable Polystyrene Beads (3000 Series) **Thermo Fisher Scientific**
- Falcon® 5 mL Round Bottom Polystyrene Test Tube with Snap Cap Sterile Individually Wrapped **Corning Catalog #352003**

1 Calculate the stock traceable size calibration reference bead particle concentration using percent solids value and particle density provided by the manufacturer and the following formula, whereis the concentration (particles mL^{-1}),, is the percent solids,is the particle density (g mL^{-1}), andis the average diameter (μm).

$$N_P=\frac{(W_V\%,\times 6\times10^{12})}{(\pi\rho_\rho D^3)}$$

Note

For example, 100 nm polystyrene beads at 1% with 1.05 g mL^{-1}would be calculated using:

$$1.82\times10^{13}=\frac{(0.01\times 6\times10^{12})}{(\pi\times1.05\times 0.1^3)}$$

A online calculator can also be found at this link.

2 Thoroughly vortex the traceable size calibration reference bead stock bottles to homogenize the mixtures before dispensing 1 drop (~50 μL) into separate 500 μL low-protein binding Eppendorf.

3 Using the working stock from step 2, make up 500 µL solution at $1x10^7$ particles mL^{-1}.

Note

It is recommended that serial dilutions are used and volumes of no less than 10 µL to avoid pipetting errors. The optimal particle concentration at which to run the reference materials will vary depending on several factors, including the flow rate, beam height, and electronic sampling rate. If running for the first time, it is recommended that serial dilutions are performed to determine the optimal concentration for preparation of the beads.

4 On the flow cytometer, set the triggering threshold to the most sensitive light scatter detector and ensure the parameter is using log-scaling (not linear or biexponential).

5 Running DPBS, lower the triggering threshold until the noise floor of the instrument becomes visible. This is most clearly when using a histogram.

5.1 Plotting the trigger-channel height parameter against time and monitoring while running DPBS is a good indication for determining whether an instrument is clean. If the spread of noise (and event rate) decreases over time, it is indicative that the instrument was dirty and is becoming cleaner.

Note

There are a number of cleaning solutions. Some shared resource lab managers have a preference. These include bleach, contrad 70, micro 90, surfanol

5.2 The extent to which the opto-electronic noise of an instrument can be sampled will vary between instruments. Legacy flow cytometers will tolerate a couple of 1000-2000 events/second whilst allowing room to sample desired events, while high-speed jet-in-air sorters are capable of sample 10,000+ events per second.

5.3 Triggering using a light scatter parameter on the opto-electronic noise of the instrument has benefits in determining and tracking the lower limit of detection, as well as being informative for buffer + reagent controls where background fluorescence will show clear shifts due to many events being triggered from sampling the noise. The use of this method comes at the cost of having high event rates and therefore larger files. Before utilizing this method the instrument should be validated to determine: 1) its ability to detect and accurately process

particles, 2) the event rate at which single small particles are detected, and 3) the degree to which the opto-electronic noise can be sampled without creating artefacts or reducing the ability to detect genuine events.

5.4 On some instruments that utilize peristaltic pumps there can appear to be an increase and decrease of the baseline corresponding to the turnover of the pump. This is a result of the threshold being set close to (but above) the electronic noise, resulting in the increase and decrease in trigger events in light scatter. This can be overcome by lowering the threshold so that the noise is being sampled regardless of the peristaltic pump turnover or increasing the threshold and therefore decreasing the instrument's limit of sensitivity.

6 Analyze each bead sample at the same acquisition settings until >5000 bead events are recorded.

6.1 It is preferable to analyze and store bead populations individually. This will minimize population overlap, aggregates, background noise, and artifacts.

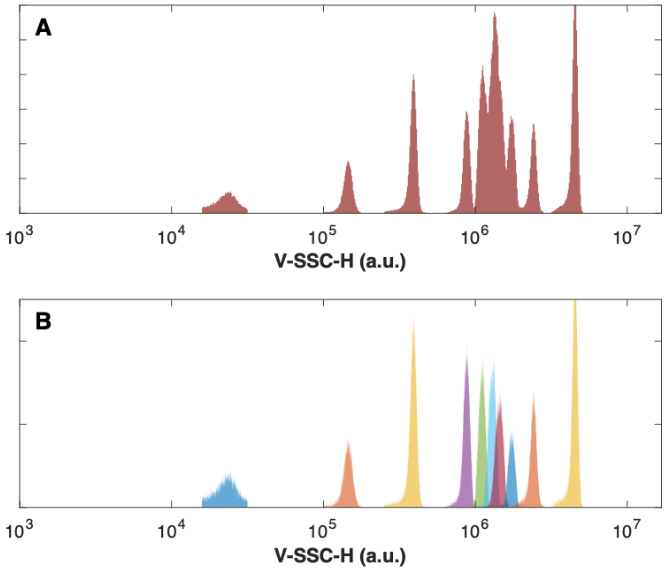

Panel A demonstrates the cumulative distribution of the gated populations when mixed together. While some populations are clearly distinguished some are not. The areas where bunching of populations occurs is dependent upon the cytometer and is useful in determining the collection angle. Panel B illustrates overlaid and colored gated bead population from Oanel A.

7 Gate each bead population using the parameter Height vs. Area in a dot-plot to remove doublets/aggregates and then use a histogram on the light scatter parameter (Height) to obtain statistics for each population. The light scatter parameter should use log scaling.

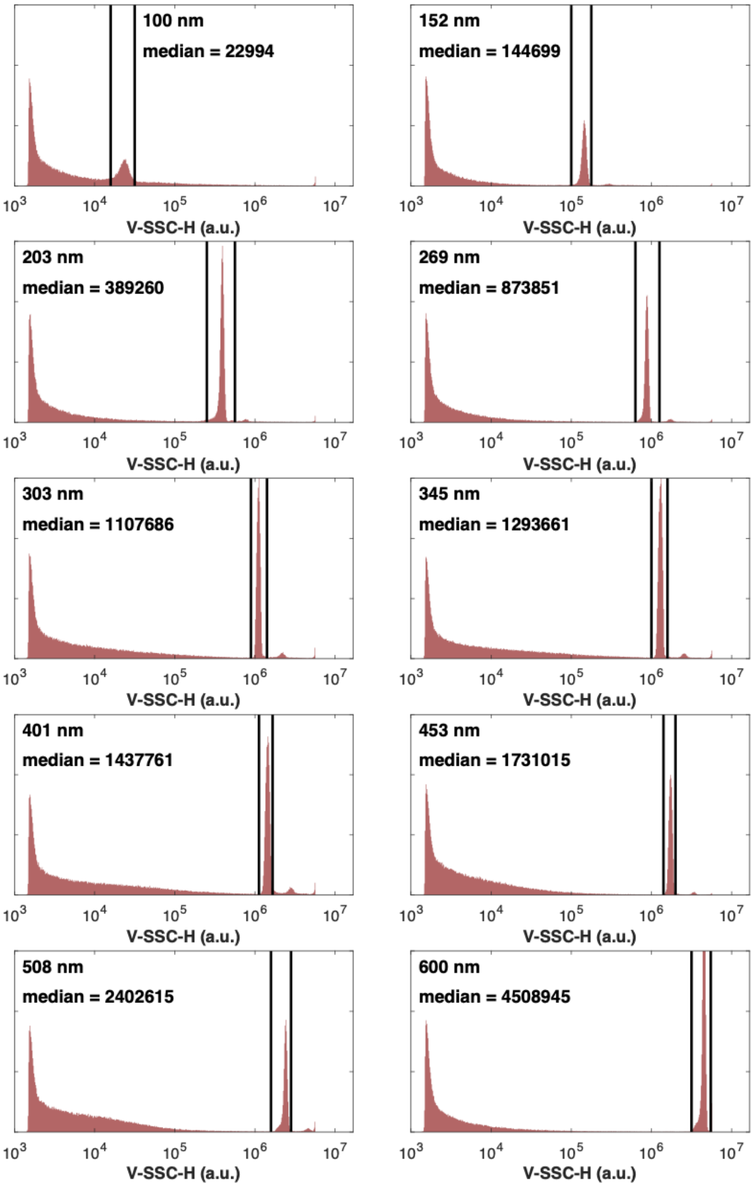

Gating light scatter reference beads. Each panel shows the gating of polystyrene NIST-traceable reference beads ranging in mean diameter from 100 to 600 nm. The median light scatter statistic of the gated population is given in each panel

8 Obtain the median statistic for each of the bead populations.

8.1 By default, flow cytometers trigger the acquisition of an event using the pulse height parameter. In cases where a trigger threshold is being defined (e.g. SSC), it is recommended that the pulse-height is used so that the limit of detection can be defined in calibrated units. There is no consensus within the small particle community over the use of pulse height vs. area. We recommend that, in general, if the parameter being calibrated was not used as a trigger channel the pulse area statistic should be used due to the tendency for low signal intensities to be linear and therefore a more reliable method for extrapolation.

D. Methods S4: Flow cytometer rEV serial dilution, related to Figure 1.

Resource 4: rEV Serial Dilution

Forked from rEV Acquisition

In 1 collection

Sean M Cook[1], Vera A. Tang[2], Joanne Lannigan[3], Jennifer Jones[1], Joshua A Welsh[4]

[1]Laboratory of Pathology, Translational Nanobiology Section, Centre for Cancer Research, National Institute of Health, National Institutes of Health; [2]University of Ottawa; [3]Flow Cytometry Support Services; [4]Translataional Nanobiology Section, Laboratory of Pathology, Center for Cancer Research, National Cancer Institute, National Institutes of Health

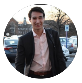

Sean M Cook

Protocol Info: Sean M Cook, Vera A. Tang, Joanne Lannigan, Jennifer Jones, Joshua A Welsh . Resource 4: rEV Serial Dilution. **protocols.io** https://protocols.io/view/resource-4-rev-serial-dilution-cnjkvckw

Created: Jan 30, 2023

Last Modified: Mar 25, 2023

PROTOCOL integer ID: 76108

DISCLAIMER

This protocol summarizes key steps for a specific type of method, which is one of a collection of methods and assays used for EV analysis in the NCI Translational Nanobiology Section at the time of submission of this protocol. Appropriate use of this protocol requires careful, cohesive integration with other methods for EV production, isolation, and characterization.

MATERIALS

Exosome standards fluorescent **Merck MilliporeSigma (Sigma-Aldrich) Catalog #SAE0193**
, referred to as 'rEVs'

DPBS **Thermo Fisher Scientific Catalog #14190144** , referred to as 'DPBS'

Low Protein Binding Collection Tubes (1.5 mL) **Thermo Fisher Scientific Catalog #90410**
, referred to as 'low-binding Eppendorf tubes'

QbSure **Cytek Catalog #B7-10005** , referred to as 'QbSure'

100 nm polystyrene NIST bead **Thermo Fisher Scientific Catalog #3100A**
, referred to as '100 nm NIST bead'

rEV Reconstitution

1 Briefly centrifuge 100 x g, 4°C, 00:05:00 rEVs before opening. 5m

2 Add 100 µL of 4 °C deionized water.

3 Pipet up and down to mix.

Note

Do not vortex

4 Aliquot 20 µL into low-binding Eppendorf tubes.

Equipment

**Low Protein Binding Microcentrifuge Tubes**	NAME
Microcentrifuge Tubes	TYPE
Thermo Scientific	BRAND
90410	SKU
https://www.thermofisher.com/order/catalog/product/90410	LINK

5 Store at 2-4 °C for up to one day (up to 2 weeks at -80 °C).

rEV Dilutions

6 Label 10 low-binding Eppendorf tubes:

A	B
Label	Dilution
1	50
2	100

A	B
3	200
4	400
5	800
6	1600
7	3200
8	6400
9	12800
10	25600

7 Add 980 µL of DPBS (preferably 0.1 µm filtered) to the tube 1 from go to step #6.

8 Add 500 µL of DPBS (preferably 0.1 µm filtered) to the remaining tubes.

9 Add 20 µL of the stock reconstituted rEVs to the tube labeled 1 (go to step #6), pipet up and down to mix.

Note

Do not vortex

10 Pipette 500 µL of the 1:50 dilution in tube 1 into the 1:100 dilution in tube 2, pipet up and down to mix.

Note

Do not vortex

11 Repeat the above step for each of the remaining dilution tubes until the serial dilution has been completed for all dilutions.

12 Keep on ice during acquisition.

Flow Cytometer Acquisition

13 Use the instrument settings that were established from the previous gain incrementation and scatter calibration resources. On the Aurora, set the window extension to 0. On the CytoFLEX, use the high acquisition mode. Collect for at least 1 minute at a low flow rate with a 30 second recording delay if using plate mode.

> **Note**
>
> To set the window extension to 0 on the Cytek Aurora, navigate to the 'Lasers' tab under the sample acquisition settings and set the window extension to 0.
>
> To change the acquisition mode on the CytoFLEX S, click on the 'Advanced' menu on the top-of-the-screen ribbon. Next click on 'Event Rate Settings' menu, change the acquisition mode to 'High', and press 'Ok'.

13.1 Set your instrument to trigger on the most sensitive side scatter channel at the FCM$_{PASS}$ optimal gain output as determined by the FluoSpheres scatter voltration. Set the threshold in the noise such that the event rate is ~1000 events/sec when running DPBS.

> **Protocol**
>
> NAME
>
> **Resource 1: Scatter Detector Setting Incrementation for FCMPASS**
>
> CREATED BY
>
> Sean M Cook
>
> PREVIEW

13.2 Set the FITC parameter to the FCM$_{PASS}$ optimal gain output as determined by the QbSure fluorescent voltration.

Protocol

NAME

Resource 2: Fluorescence Detector Setting Incrementation for FCMPASS

CREATED BY

Sean M Cook

PREVIEW

14 Collect a DPBS sample in a FACS tube for one minute on low.

Note

This is to ensure the instrument is clean on the side scatter and fluorescent channels before acquiring the rEVs.

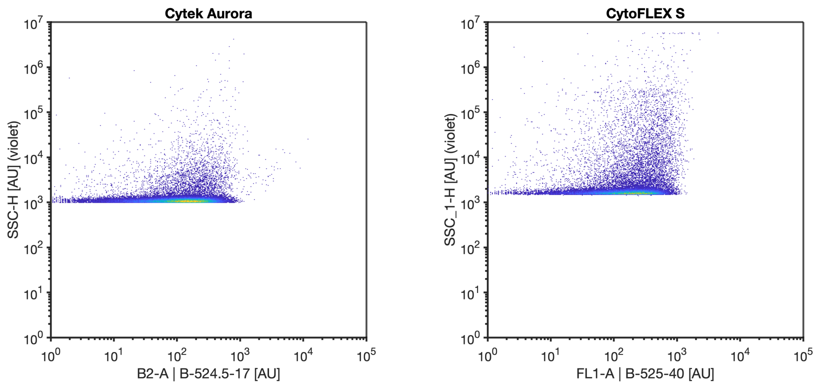

DPBS acquired on the Cytek Aurora and CytoFLEX S.

15 Collect all rEVs samples for one minute on low, starting with the highest dilution (least concentrated) and working down to lowest dilution (most concentrated). This will reduce the effect of carry over from high concentrations.

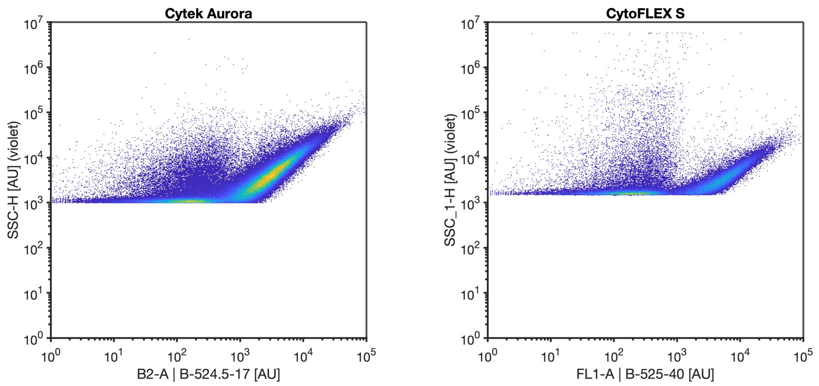

rEVs acquired on the Cytek Aurora and CytoFLEX S.

16 Run a DPBS sample in a FACS tube to make sure there are no carryover events; there should be no events in the B1 and B2 channel.

Note

If there is carry over clean instrument with bleach and flush with deionized water as appropriate.

17 Label as FACS tube as 'QbSure', and add 500 µL DPBS. Vortex the QbSure beads for 5 sec and add 3 drops QbSure beads into FACS tube. Run QbSure beads on low and collect 10,000 bead events.

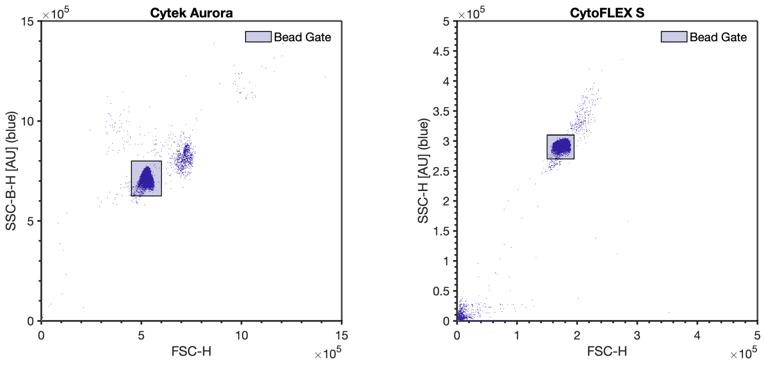

QbSure beads gated on blue SSC-H and FSC-H on the Cytek Aurora and CytoFLEX S. The violet SSC settings remain unchanged. In order to limit .fcs file size, a FSC scatter trigger may be used until 10000 QbSure bead events are acquired.

> **Note**
>
> You may need to adjust the B-SSC and FSC detector settings for the bead acquisition. Do not adjust fluorescence detector settings or high sensitivity SSC detector settings. Assuming the QbSure beads were cross calibrated in the previous protocols, this allows for the calibration of the rEV fluorescent data into calibrated units.

18 Run the 100 nm polystyrene NIST beads at the same settings as the rEVs (diluted in DPBS to a concentration of 5E6 p/mL) until at least 10000 bead events have been acquired.

> **Note**
>
> Acquiring the 100 nm polystyrene NIST bead allows for the calibration of the rEV side scatter data into calibrated units if the collection half-angle of the instrument has been determined in previous protocols.

19 If calibration of data into standard units is desired, the 100 nm polystyrene NIST bead and QbSure beads can be used to calibrate data by following the FCM_{PASS} experiment calibration protocols.

Protocol

NAME

FCMPASS Protocol Collection

CREATED BY

Joshua A Welsh

PREVIEW

E. Methods S5: Flow cytometer rEV light scatter detector setting incrementation, related to Figure 1.

Resource 5: rEV Scatter Detector Setting Incrementation

In 1 collection

Sean M Cook[1], Vera A. Tang[2], Joanne Lannigan[3], Jennifer Jones[1], Joshua A Welsh[1]

[1]Translataional Nanobiology Section, Laboratory of Pathology, Center for Cancer Research, National Cancer Institute, National Institutes of Health; [2]University of Ottawa; [3]Flow Cytometry Support Services

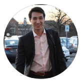

Sean M Cook

Protocol Info: Sean M Cook, Vera A. Tang, Joanne Lannigan, Jennifer Jones, Joshua A Welsh . Resource 5: rEV Scatter Detector Setting Incrementation. **protocols.io** https://protocols.io/view/resource-5-rev-scatter-detector-setting-incrementa-cnjpvcmn

Created: Jan 30, 2023

Last Modified: Mar 25, 2023

PROTOCOL integer ID: 76111

DISCLAIMER

This protocol summarizes key steps for a specific type of method, which is one of a collection of methods and assays used for EV analysis in the NCI Translational Nanobiology Section at the time of submission of this protocol. Appropriate use of this protocol requires careful, cohesive integration with other methods for EV production, isolation, and characterization.

MATERIALS

Exosome standards fluorescent **Merck MilliporeSigma (Sigma-Aldrich) Catalog #SAE0193**
, referred to as 'rEVs'

DPBS **Thermo Fisher Scientific Catalog #14190144** , referred to as 'DPBS'

5 mL Round-bottom tube **Corning Catalog #352052** , referred to as 'FACS tube'

Protein LoBind Tubes (5 mL) **Thermo Fisher Scientific Catalog #0030122356**
, referred to as 'low-binding Eppendorf tubes'

QbSure **Cytek Catalog #B7-10005** , referred to as 'QbSure'

100 nm polystyrene NIST bead **Thermo Fisher Scientific Catalog #3100A**
, referred to as '100 nm NIST bead'

Sample Prepreparation

5m

1 Briefly centrifuge rEVs at 100 x g, 4°C, 00:05:00 before opening

5m

2 Add 100 µL of 4 °C deionized water to rEVs vial. Pipette up and down to mix well.

Note

Do not vortex

3 Create 5 mL of a 1:2000 dilution of rEVs by pipetting 1 µL rEVs into 1999 µL DPBS in a low-binding Eppendorf tube. This gives a 5E6 p/mL solution.

4 Label a FACS tube as 'DPBS'. Pipette 500 µL DPBS into the tube.

5 Label as FACS tube as 'QbSure', and add 500 µL DPBS. Vortex the QbSure beads for 5 sec and add 3 drops QbSure beads into FACS tube.

Cytometer Setup

6 Ensure cytometer is clean and that -Height and -Area statistics are set to be collected on all parameters and that all parameters are on.

7 On the Cytek Aurora, set window extension to 0. On the CytoFLEX platform turn on 'High Acquisition Mode'.

Note

To set the window extension to 0 on the Cytek Aurora, navigate to the 'Lasers' tab under the sample acquisition settings and set the window extension to 0.

To change the acquisition mode on the CytoFLEX S, click on the 'Advanced' menu on the top-of-the-screen ribbon. Next click on 'Event Rate Settings' menu, change the acquisition mode to 'High', and press 'Ok'.

8 Create a histogram plot with the FITC parameter (CytoFLEX: FL1 | B-525-40, Aurora: B2 | B-524.5-17) on the X-Axis and make sure it is plotted on a log-scale.

9 Create a histogram plot with (405 nm) V-SSC-H on the X-Axis and make sure it is plotted on a log-scale.

10 Set the cytometer triggering threshold to the FITC parameter at the same settings used to acquire the bead scatter voltration . **All samples should be acquired with the lowest flow rate, typically ~10-15 µL min^{-1}.**

11 Acquire the 'DPBS' tube while viewing the FITC histogram plot from go to step #9 . Adjust the detector gain or trigger threshold until the instrument noise is being **acquired at ~1000 events/sec.** The instrument noise floor is distinct from detected background events in sheath as it has a sharp increase. In a system with debris there may be a tail that elongates out of this this sharp peak.

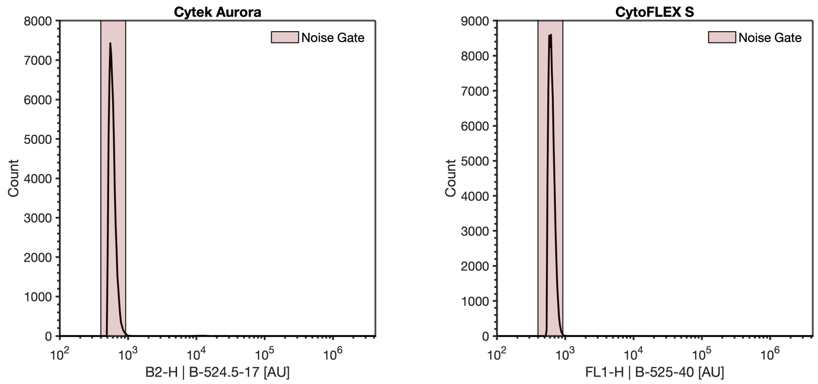

Acquisition of DPBS on the Cytek Aurora and CytoFLEX S, with the Aurora triggering on instrument noise on the B2-H channel at gain 1750 and threshold 500 and the CytoFLEX triggering on instrument noise on the FL1-H channel at gain 500 and threshold 550.

11.1 Recording this noise is not necessary as this step is for identifying optimal settings.

rEV Acquisition

12 Validation of FCM_{PASS} outputs can now be performed by acquiring the rEVs at the same voltration gains used when acquiring the FluoSpheres scatter voltration.

Note

For flow cytometers with avalanche photodiodes its is recommended that the detector settings have more incrementation at lower gains than higher gains while for instruments with photomultiplier tubes they should be spaced evenly. See template for example of settings for each tube analyses

Example Gain Voltration for CytoFLEX & Aurora

1. 50
2. 100
3. 200
4. 300
5. 400
6. 500
7. 750
8. 1000
9. 1250
10. 1500
11. 2000
12. 2500
13. 3000

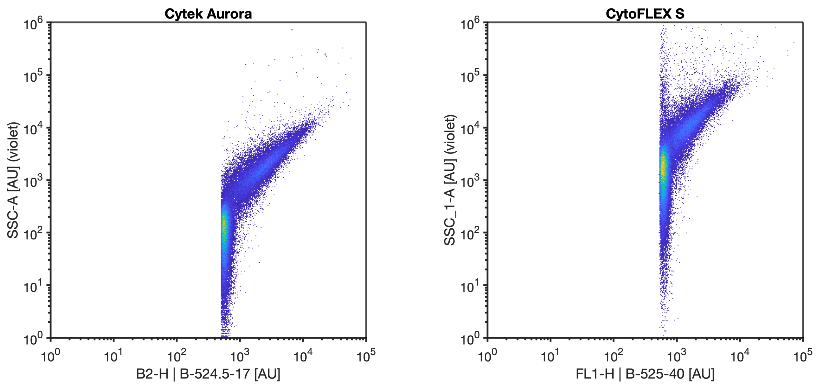

Example of rEVs acquired on the Cytek Aurora and CytoFLEX S. Cytek Aurora triggered on B2-H | B-524.5-17 at gain 1750 and threshold 500. CytoFLEX S triggered on violet FL1-H | B-525-40 at gain 500 and threshold 550.

Note

The total event rate when acquiring the rEVs should not exceed 6000 events/sec. If the event rate is higher than 6000 events/sec, further dilution of the rEVs is necessary prior to acquisition.

13 Pipette 200 µL from the 5E6 rEV solution from go to step #3 into a FACS tube. Acquire the FACS tube for 60 s on the lowest flow rate at one of the scatter gains. Repeat this for each gain.

Note

In order to prevent the crashing out of rEVs over the time course of the experiment, only pipette from 5E6 rEV solution into a FACS tube immediately prior to acquisition. Reverse pipette to mix in the FACS tube, do not vortex.

14 Run the 100 nm polystyrene NIST beads at the same settings as the rEVs (diluted in DPBS to a concentration of 5E6 p/mL) until at least 10000 bead events have been acquired for each gain.

Note

Acquiring the 100 nm polystyrene NIST bead allows for the calibration of the rEV side scatter data into calibrated units if the collection half-angle of the instrument has been determined in previous protocols.

15 Run QbSure beads on low at the same fluorescent settings as the rEVs and collect 10,000 bead events. QbSure beads only need to be acquired once at the fluorescent settings used in the experiment.

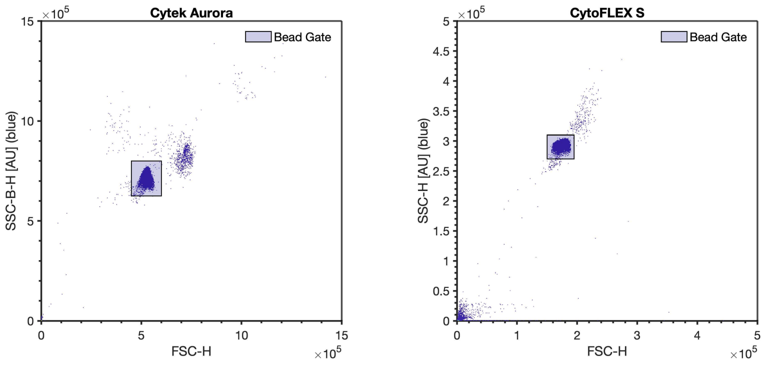

QbSure beads gated on blue SSC-H and FSC-H on the Cytek Aurora and CytoFLEX S. The violet SSC settings remain unchanged. In order to limit .fcs file size, a FSC scatter trigger may be used until 10000 QbSure bead events are acquired.

Note

You may need to adjust the B-SSC and FSC detector settings for the bead acquisition. Do not adjust fluorescence detector settings or high sensitivity SSC detector settings. Assuming the QbSure beads were cross calibrated in the previous protocols, this allows for the calibration of the rEV fluorescent data into calibrated units.

16 If calibration of data into standard units is desired, the 100 nm polystyrene NIST bead and QbSure beads can be used to calibrate data by following the FCM_{PASS} experiment calibration protocols.

Protocol

NAME

FCMPASS Protocol Collection

CREATED BY

Joshua A Welsh

PREVIEW

Note

Not all scatter gains will resolve the 100 nm NIST bead, but the data can still be calibrated using the 110 nm FluoSpheres that were collected in the FluoSpheres scatter voltration.

F. Methods S6: Flow cytometer rEV fluorescence detector setting incrementation, related to Figure 1.

Resource 6: rEV Fluorescent Detector Setting Incrementation

In 1 collection

Sean M Cook[1], Vera A. Tang[2], Joanne Lannigan[3], Jennifer Jones[1], Joshua A Welsh[1]

[1]Translataional Nanobiology Section, Laboratory of Pathology, Center for Cancer Research, National Cancer Institute, National Institutes of Health; [2]University of Ottawa; [3]Flow Cytometry Support Services

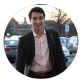

Sean M Cook

Protocol Info: Sean M Cook, Vera A. Tang, Joanne Lannigan, Jennifer Jones, Joshua A Welsh . Resource 6: rEV Fluorescent Detector Setting Incrementation. **protocols.io** https://protocols.io/view/resource-6-rev-fluorescent-detector-setting-increm-cnjivcke

Created: Jan 30, 2023

Last Modified: Mar 25, 2023

PROTOCOL integer ID: 76106

DISCLAIMER

This protocol summarizes key steps for a specific type of method, which is one of a collection of methods and assays used for EV analysis in the NCI Translational Nanobiology Section at the time of submission of this protocol. Appropriate use of this protocol requires careful, cohesive integration with other methods for EV production, isolation, and characterization.

MATERIALS

Exosome standards fluorescent **Merck MilliporeSigma (Sigma-Aldrich) Catalog #SAE0193**, referred to as 'rEVs'

DPBS **Thermo Fisher Scientific Catalog #14190144**, referred to as 'DPBS'

5 mL Round-bottom tube **Corning Catalog #352052**, referred to as 'FACS tube'

Protein LoBind Tubes (5 mL) **Thermo Fisher Scientific Catalog #0030122356**, referred to as 'low-binding Eppendorf tubes'

QbSure **Cytek Catalog #B7-10005**, referred to as 'QbSure'

100 nm polystyrene NIST bead **Thermo Fisher Scientific Catalog #3100A**, referred to as '100 nm NIST bead'

Sample Prepreparation

5m

1 Briefly centrifuge rEVs at 100 x g, 4°C, 00:05:00 before opening 5m

1

2 Add 100 µL of 4 °C deionized water to rEV vial. Pipette up and down to mix well.

Note

Do not vortex

3 Create 5 mL of a 1:2000 dilution of rEVs by pipetting 1 µL rEVs into 1999 µL DPBS in a low-binding Eppendorf tube. This gives a 5E6 p/mL solution.

4 Label a FACS tube as 'DPBS'. Pipette 500 µL DPBS into the tube.

5 Label as FACS tube as 'QbSure', and add 500 µL DPBS. Vortex the QbSure beads for 5 sec and add 3 drops QbSure beads into FACS tube.

Cytometer Setup

6 Ensure cytometer is clean and that -Height and -Area statistics are set to be collected on all parameters and that all parameters are on.

7 On the Cytek Aurora, set window extension to 0. On the CytoFLEX platform turn on 'High Acquisition Mode'.

Note

To set the window extension to 0 on the Cytek Aurora, navigate to the 'Lasers' tab under the sample acquisition settings and set the window extension to 0.

To change the acquisition mode on the CytoFLEX S, click on the 'Advanced' menu on the top-of-the-screen ribbon. Next click on 'Event Rate Settings' menu, change the acquisition mode to 'High', and press 'Ok'.

8 Create a histogram plot with the FITC parameter (CytoFLEX: FL1 | B-525-40, Aurora: B2 | B-524.5-17) on the X-Axis and make sure it is plotted on a log-scale.

9 Create a histogram plot with (405 nm) V-SSC-H on the X-Axis and make sure it is plotted on a log-scale.

10 Set the cytometer triggering threshold to the violet SSC parameter at the same settings used to acquire the bead fluorescent voltration. **All samples should be acquired with the lowest flow rate, typically ~10-15 µL min^{-1}.**

11 Acquire the 'DPBS' tube while viewing the SSC histogram plot from go to step #9. Adjust the detector gain or trigger threshold until the instrument noise is being **acquired at ~1000 events/sec.** The instrument noise floor is distinct from detected background events in sheath as it has a sharp increase. In a system with debris there may be a tail that elongates out of this this sharp peak.

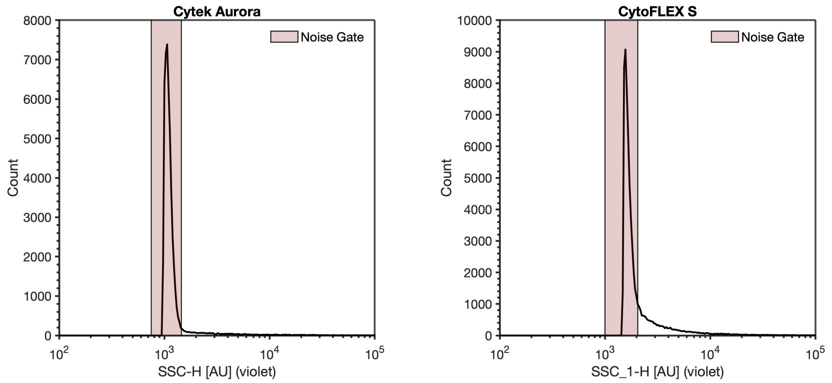

Example of threshold on the instrument noise floor on the Cytek Aurora and CytoFLEX S. Cytek Aurora triggered on SSC-H channel at gain 2500 and threshold 975. CytoFLEX S triggered on SSC_1-H channel at gain 250 and threshold 1200.

11.1 Recording this noise is not necessary as this step is for identifying optimal settings.

rEV Acquisition

12 Validation of FCM_{PASS} outputs can now be performed by acquiring the rEVs at the same voltration gains used when acquiring the QbSure fluorescence voltration.

Note

For flow cytometers with avalanche photodiodes its is recommended that the detector settings have more incrementation at lower gains than higher gains while for instruments with photomultiplier tubes they should be spaced evenly. See template for example of settings for each tube analyses

Example Gain Voltration for CytoFLEX & Aurora

1. 50
2. 100
3. 200
4. 300
5. 400
6. 500
7. 750
8. 1000
9. 1250
10. 1500
11. 2000
12. 2500
13. 3000

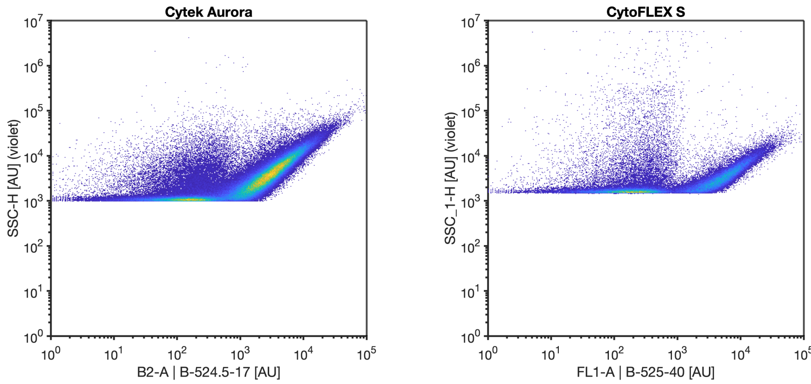

Example of rEVs acquired on the Cytek Aurora and CytoFLEX S. Cytek Aurora triggered on violet SSC-H at gain 2500 and threshold 975. CytoFLEX S triggered on violet SSC_1-H at gain 250 and threshold 1200.

Note

The total event rate when acquiring the rEVs should not exceed 6000 events/sec. If the event rate is higher than 6000 events/sec, further dilution of the rEVs is necessary prior to acquisition.

13 Pipette 200 µL from the 5E6 rEV solution from go to step #3 into a FACS tube. Acquire the FACS tube for 60 s on the lowest flow rate at one of the fluorescence gains. Repeat this for each gain.

Note

In order to prevent the crashing out of rEVs over the time course of the experiment, only pipette from 5E6 rEV solution into a FACS tube immediately prior to acquisition. Reverse pipette to mix in the FACS tube, do not vortex.

14 Run the 100 nm polystyrene NIST beads at the same scatter settings as the rEVs (diluted in DPBS to a concentration of 5E6 p/mL) until at least 10000 bead events have been acquired. The 100 nm bead only needs to be acquired once at the scatter settings used in the experiment.

Note

Acquiring the 100 nm polystyrene NIST bead allows for the calibration of the rEV side scatter data into calibrated units if the collection half-angle of the instrument has been determined in previous protocols.

15 Run QbSure beads on low at the same fluorescent settings and collect 10,000 bead events for each gain.

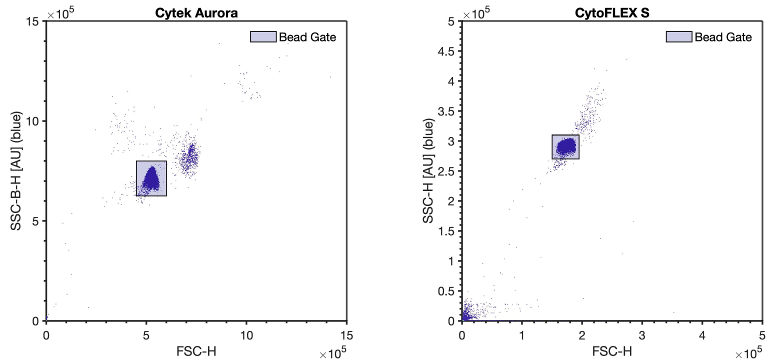

QbSure beads gated on blue SSC-H and FSC-H on the Cytek Aurora and CytoFLEX S. The violet SSC settings remain unchanged. In order to limit .fcs file size, a FSC scatter trigger may be used until 10000 QbSure bead events are acquired.

Note

You may need to adjust the B-SSC and FSC detector settings for the bead acquisition. Do not adjust fluorescence detector settings or high sensitivity SSC detector settings. Assuming the QbSure beads were cross calibrated in the previous protocols, this allows for the calibration of the rEV fluorescent data into calibrated units.

16 If calibration of data into standard units is desired, the 100 nm polystyrene NIST bead and QbSure beads can be used to calibrate data by following the FCM_{PASS} experiment calibration protocols.

Protocol

NAME

FCMPASS Protocol Collection

CREATED BY

Joshua A Welsh

PREVIEW

G. Methods S7: Flow cytometer rEV immunophenotyping, related to Figure 1.

Resource 7: rEV immunophenotyping

In 1 collection

Sean M Cook[1], Vera A. Tang[2], Joanne Lannigan[3], Jennifer Jones[1], Joshua A Welsh[4]

[1]Laboratory of Pathology, Translational Nanobiology Section, Centre for Cancer Research, National Institute of Health, National Institutes of Health; [2]University of Ottawa; [3]Flow Cytometry Support Services; [4]Translataional Nanobiology Section, Laboratory of Pathology, Center for Cancer Research, National Cancer Institute, National Institutes of Health

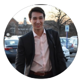

Sean M Cook

Protocol Info: Sean M Cook, Vera A. Tang, Joanne Lannigan, Jennifer Jones, Joshua A Welsh . Resource 7: rEV immunophenotyping. **protocols.io** https://protocols.io/view/resource-7-rev-immunophenotyping-cnjsvcne

Created: Jan 30, 2023

Last Modified: Mar 25, 2023

PROTOCOL integer ID: 76114

DISCLAIMER

This protocol summarizes key steps for a specific type of method, which is one of a collection of methods and assays used for EV analysis in the NCI Translational Nanobiology Section at the time of submission of this protocol. Appropriate use of this protocol requires careful, cohesive integration with other methods for EV production, isolation, and characterization.

MATERIALS

Exosome standards fluorescent **Merck MilliporeSigma (Sigma-Aldrich) Catalog #SAE0193**
, referred to as 'rEVs'

DPBS **Thermo Fisher Scientific Catalog #14190144** , referred to as 'DPBS'

5 mL Round-bottom tube **Corning Catalog #352052** , referred to as 'FACS tube'

Protein LoBind Tubes (5 mL) **Thermo Fisher Scientific Catalog #0030122356**
, referred to as 'low-binding Eppendorf tubes'

Low Protein Binding Microcentrifuge **Thermo Fisher Scientific Catalog #88379**

96-well V bottom plate **Contributed by users**

PE anti-human CD81 (TAPA-1) Antibody **BioLegend Catalog #349505** , referred to as 'anti-CD81-PE'

APC anti-human CD81 (TAPA-1) Antibody **BioLegend Catalog #349509** , referred to as 'anti-CD81-APC'

Pacific Blue™ anti-human CD81 (TAPA-1) Antibody **BioLegend Catalog #349515**
, referred to as anti-CD81-PB'

QbSure **Cytek Catalog #B7-10005** , referred to as 'QbSure'

100 nm polystyrene NIST bead **Thermo Fisher Scientific Catalog #3100A**
, referred to as '100 nm NIST bead'

Sample Preparation

35m

1 Briefly centrifuge the rEVs 100 x g, 4°C, 00:05:00 before opening 5m

2 Add 100 µL of 4 °C deionized water. Pipette up and down to mix.

Note

Do not vortex

3 Dilute the reconstituted rEVs 1 in 5 in PBS for staining* Take 50 µL of the rEV stock and add

2

200 µL of PBS.

Note

The sample concentration chosen for staining should take into consideration the need to dilute the sample further after staining to reduce the concentration of free antibodies, since the samples will be acquired without a washing step to remove these antibodies as in the case of cells.

4 Prepare the antibody dilutions for a 2x staining concentration of each of the antibodies to be used in the antibody titration. See the example below for sample calculations prepared for a staining concentration of 2 µg/ml.

A	B	C	D	E	F
	AB stock concentration (µg/ml)	2x staining conc (µg/ml)	**Stock volume for 2x staining conc (µL)**	Total volume for 2x staining conc (µL)	**Volume of PBS to be added (uL)**
anti-Human CD81-PE	120	4	**2**	60	**58**
anti-Human CD81-APC	200	4	**2**	100	**98**
anti-Human CD81-PB	300	4	**2**	150	**148**

Sample calculations prepared for a staining concentration of 2 µg/ml. All Antibodies clones are 5A6.

5 Samples will now be prepared using the following plate map.

A	B	C	D	E	F	G	H
	**CD81 PE**	**CD81 APC**	**CD81 Pacific Blue**	**CD81 PE**	**CD81 APC**	**CD81 Pacific Blue**	
**Sample**	rEV	rEV	rEV	PBS	PBS	PBS	
**0 µg/mL**	A1	A2	A3	A4	A5	A6	
**0.0625 µg/mL**	B1	B2	B3	B4	B5	B6	
**0.125 µg/mL**	C1	C2	C3	C4	C5	C6	
**0.25 µg/mL**	D1	D2	D3	D4	D5	D6	
**0.5 µg/mL**	E1	E2	E3	E4	E5	E6	
**1 µg/mL**	F1	F2	F3	F4	F5	F6	

	A	B	C	D	E	F	G	H
	**2 µg/mL**	G1	G2	G3	G4	G5	G6	**QbSure Beads**
		PBS	PBS	PBS	PBS	PBS	PBS	**100 nm PS NIST-Traceable Beads**

6 In a 96-well V-bottom plate, add 10 µL 1:5 rEV solution to wells A1-A3

7 Add 10 µL DPBS to wells A1-A3 and reverse pipet to mix. These wells will serve as the rEV controls

8 Add 20 µL DPBS to wells A4-A6. These wells will serve as buffer only controls

9 In the same 96-well V-bottom plate from go to step #6, add 10 µL 1:5 rEV solution to wells B1-G3. Add 10 µL DPBS to wells A1-G1. Add 10 µL from the PE working solution tubes to wells B1-G1, add 10 µL from the APC working solution tubes to wells B2-G2, add 10 µL from the PB working solution tubes to wells B3-G3.

10 In the same 96-well V-bottom plate from go to step #6, add 10 µL from the corresponding PE working solutions tube to wells B4-G4. Add 10 µL from the corresponding APC working solutions tube to wells B5-G5. Add 10 µL from the corresponding PB working solutions tube to wells B6-G6. These wells will serve as the DBPS+AB control wells.

11 Cover and incubate this plate for 00:30:00 at RT. 30m

12 Using a separate 96-well V-bottom plate, add 199 µL DPBS into wells A1-G6. Pipet 1 µL from the incubation plate into the new plate in the same wells and reverse pipette to mix.

4

Note

Steps 11 and 12 will be repeated for the ON incubations as well to bring rEVs down to running concentration that doesn't swarm the instrument detectors.

13 Add 200 µL DPBS into wells A7-G7. These wells will help reduce sample carryover after each rEV+AB combination is acquired.

14 Re-cover incubation plate and let incubate ON at RT to repeat measurements next day as directed in the following steps.

15 Label as FACS tube as 'QbSure', and add 500 µL DPBS. Vortex the QbSure beads for 5 sec and add 3 drops QbSure beads into FACS tube.

Cytometer Setup

16 Setup cytometer to acquire at FCM_{PASS} output gains for both light scatter and fluorescent detectors as determined by the FCM_{PASS} detector optimization module outputs. For the Aurora, an optimal gain template is returned to import into the Spectraflo software.

17 On the Cytek Aurora, set window extension to 0. On the CytoFLEX platform turn on 'High Acquisition Mode'.

Note

To set the window extension to 0 on the Cytek Aurora, navigate to the 'Lasers' tab under the sample acquisition settings and set the window extension to 0.

To change the acquisition mode on the CytoFLEX S, click on the 'Advanced' menu on the top-of-the-screen ribbon. Next click on 'Event Rate Settings' menu, change the acquisition mode to 'High', and press 'Ok'.

18 Set the cytometer triggering threshold to the violet SSC parameter and run a DPBS control first to ensure the cytometer is clean and thresholds/event rates have remained unchanged. The

background event rate should be ~1000 events/sec. **All samples should be acquired with the lowest flow rate, typically ~10-15 µL min^{-1}.**

19 Acquire all wells for at least 60 sec.

20 In an open well, run the 100 nm polystyrene NIST beads at the same settings as the rEVs (diluted in DPBS to a concentration of 5E6 p/mL) until at least 10000 bead events have been acquired.

21 In an open well, add 200 µL from the QbSure FACS tube and collect 10,000 bead events at the same settings as the rEVs.

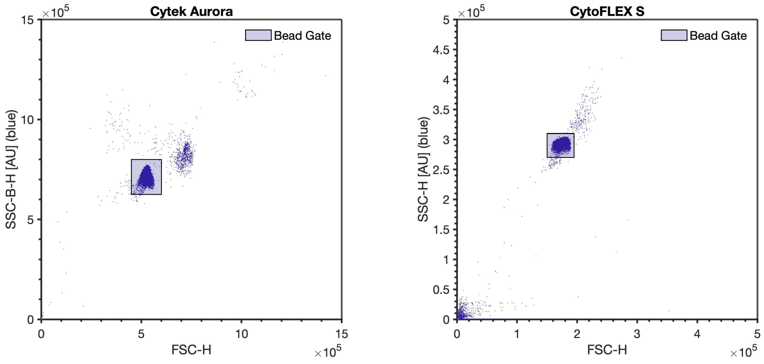

QbSure beads gated on blue SSC-H and FSC-H on the Cytek Aurora and CytoFLEX S. The violet SSC settings remain unchanged. In order to limit .fcs file size, a FSC scatter trigger may be used until 10000 QbSure bead events are acquired.

Note

You may need to adjust the B-SSC and FSC detector settings for the bead acquisition. Do not adjust fluorescence detector settings or high sensitivity SSC detector settings. Assuming the QbSure beads were cross calibrated in the previous protocols, this allows for the calibration of the rEV fluorescent data into calibrated units.

22 If calibration of data into standard units is desired, the 100 nm polystyrene NIST bead and QbSure beads can be used to calibrate data by following the FCM_{PASS} experiment calibration protocols.

Protocol

NAME

FCMPASS Protocol Collection

CREATED BY

Joshua A Welsh

PREVIEW

23 Don't forget to repeat the acquisition for the samples incubating ON.

References
